# Memory T-cell enriched haploidentical transplantation with NK cell addback results in promising long-term outcomes: a phase II trial

**DOI:** 10.1186/s13045-024-01567-0

**Published:** 2024-06-27

**Authors:** Swati Naik, Ying Li, Aimee C. Talleur, Subodh Selukar, Emily Ashcraft, Cheng Cheng, Renee M. Madden, Ewelina Mamcarz, Amr Qudeimat, Akshay Sharma, Ashok Srinivasan, Ali Y. Suliman, Rebecca Epperly, Esther A. Obeng, M. Paulina Velasquez, Deanna Langfitt, Sarah Schell, Jean-Yves Métais, Paula Y. Arnold, Diego R. Hijano, Gabriela Maron, Thomas E. Merchant, Salem Akel, Wing Leung, Stephen Gottschalk, Brandon M. Triplett

**Affiliations:** 1 Present Address: Department of Bone Marrow Transplantation & Cellular Therapy, St Jude Children’s Research Hospital, Memphis, TN USA; 2 Department of Biostatistics, St Jude Children’s Research Hospital, Memphis, TN USA; 3 Department of Pathology, St Jude Children’s Research Hospital, Memphis, TN USA; 4 Department of Infectious Diseases, St Jude Children’s Research Hospital, Memphis, TN USA; 5 Department of Radiation Oncology, St Jude Children’s Research Hospital, Memphis, TN USA; 6 Department of Pediatrics, University of Tennessee Health Science Center, Memphis, TN USA

**Keywords:** Pediatric hematological malignancies, Transplantation, Haploidentical, Memory T cell, T-cell depletion, CD45RA depletion, NK cell, Immune reconstitution, Non-TBI regimen, Graft-versus-leukemia

## Abstract

**Background:**

Relapse remains a challenge after transplantation in pediatric patients with hematological malignancies. Myeloablative regimens used for disease control are associated with acute and long-term adverse effects. We used a CD45RA-depleted haploidentical graft for adoptive transfer of memory T cells combined with NK-cell addback and hypothesized that maximizing the graft-versus-leukemia (GVL) effect might allow for reduction in intensity of conditioning regimen.

**Methods:**

In this phase II clinical trial (NCT01807611), 72 patients with hematological malignancies (complete remission (CR)1: 25, ≥ CR2: 28, refractory disease: 19) received haploidentical CD34 + enriched and CD45RA-depleted hematopoietic progenitor cell grafts followed by NK-cell infusion. Conditioning included fludarabine, thiotepa, melphalan, cyclophosphamide, total lymphoid irradiation, and graft-versus-host disease (GVHD) prophylaxis consisted of a short-course sirolimus or mycophenolate mofetil without serotherapy.

**Results:**

The 3-year overall survival (OS) and event-free-survival (EFS) for patients in CR1 were 92% (95% CI:72–98) and 88% (95% CI: 67–96); ≥ CR2 were 81% (95% CI: 61–92) and 68% (95% CI: 47–82) and refractory disease were 32% (95% CI: 11–54) and 20% (95% CI: 6–40). The 3-year EFS for all patients in morphological CR was 77% (95% CI: 64–87) with no difference amongst recipients with or without minimal residual disease (*P* = 0.2992). Immune reconstitution was rapid, with mean CD3 and CD4 T-cell counts of 410/μL and 140/μL at day + 30. Cumulative incidence of acute GVHD and chronic GVHD was 36% and 26% but most patients with acute GVHD recovered rapidly with therapy. Lower rates of grade III-IV acute GVHD were observed with NK-cell alloreactive donors (*P* = 0.004), and higher rates of moderate/severe chronic GVHD occurred with maternal donors (*P* = 0.035).

**Conclusion:**

The combination of a CD45RA-depleted graft and NK-cell addback led to robust immune reconstitution maximizing the GVL effect and allowed for use of a submyeloablative, TBI-free conditioning regimen that was associated with excellent EFS resulting in promising long-term outcomes in this high-risk population.

The trial is registered at ClinicalTrials.gov (NCT01807611).

**Supplementary Information:**

The online version contains supplementary material available at 10.1186/s13045-024-01567-0.

## Introduction

Allogeneic hematopoietic cell transplantation (HCT) can be curative for patients with high-risk hematological malignancies. This therapeutic benefit is mediated by the cytotoxic conditioning regimen and the graft-versus-leukemia (GVL) effect. Reducing the intensity of conditioning regimens and specifically avoiding total body irradiation (TBI), particularly in pediatric patients, can significantly lower the incidence of adverse effects, but is associated with higher relapse rates [1–5].

GVL is mediated by the various immune cells in the donor graft, predominantly T cells and natural killer (NK) cells. However, certain T-cell subsets can also cause severe graft-versus-host disease (GVHD) which is further amplified in the haploidentical donor setting. One approach to selectively lower the risk of GVHD is to deplete naïve, CD45RA-positive T cells that cause severe GVHD while retaining CD45RA-negative memory T-cell subsets that cause none or limited milder GVHD [6–10] but maintain immunity to pathogens and anti-leukemic properties [11–18]. NK cells also play a significant role in reducing relapse and can lower risk of infections and GVHD [12, 19–25]. Our group, along with others, have shown that using killer immunoglobulin-like receptors (KIR) typing to select NK-alloreactive donors for allogeneic HCT can enhance these effects [21–25]. Additionally, avoiding serotherapy can further optimize the GVL effect by promoting early immune reconstitution [26–29]. We hypothesized that maximizing the GVL effect by utilizing these immune effectors might allow for reduction in intensity of conditioning regimen.

We therefore developed a transplant protocol that consisted of a TBI-free, serotherapy-free, sub-myeloablative conditioning regimen followed by the sequential infusion of haploidentical CD34-enriched and CD45RA-depleted hematopoietic progenitor cell (HPC) grafts combined with an NK-cell addback derived from preferentially NK alloreactive donors. Here we report the outcomes of our prospective clinical trial using this approach in 72 pediatric patients with high-risk hematologic malignancies.

## Methods

### Patients and donors

Written informed consent was obtained in accordance with the Declaration of Helsinki. The study was approved by St. Jude’s Institutional Review Board and Investigational Device Exemption for the use of the CliniMACS Plus device (Miltenyi Biotec) was granted by the Food and Drug Administration. The trial is registered at ClinicalTrials.gov (NCT01807611).

Patients with high-risk hematologic malignancy for which HCT was indicated but who lacked a suitable Human Leukocyte Antigen (HLA) matched donor were eligible. Complete remission (CR) was defined as absence of morphological disease and included patients with evidence of minimal residual disease by flow cytometry (MRD: < 0.1% for AML, < 0.01% for ALL) and those with detectible disease (evidence of disease including positive cytogenetic, molecular or flow-cytometric testing but less than as defined above by flow MRD). Active disease was defined as > 5% blasts by morphology prior to transplant, or aplasia with the immediate prior bone marrow evaluation showing > 5% blasts by morphology. Detailed inclusion and exclusion criteria are listed in the provided protocol.

Haploidentical family donors were selected based on established criteria taking donor age, parity, Cytomegalovirus (CMV) status and blood type into account. Donor KIR profiling was also performed [30, 31]. Initially only patients with a KIR receptor-ligand mismatched haploidentical donor were eligible; subsequently this criterion was waived.

### Transplant regimen

**Fig. 1:**
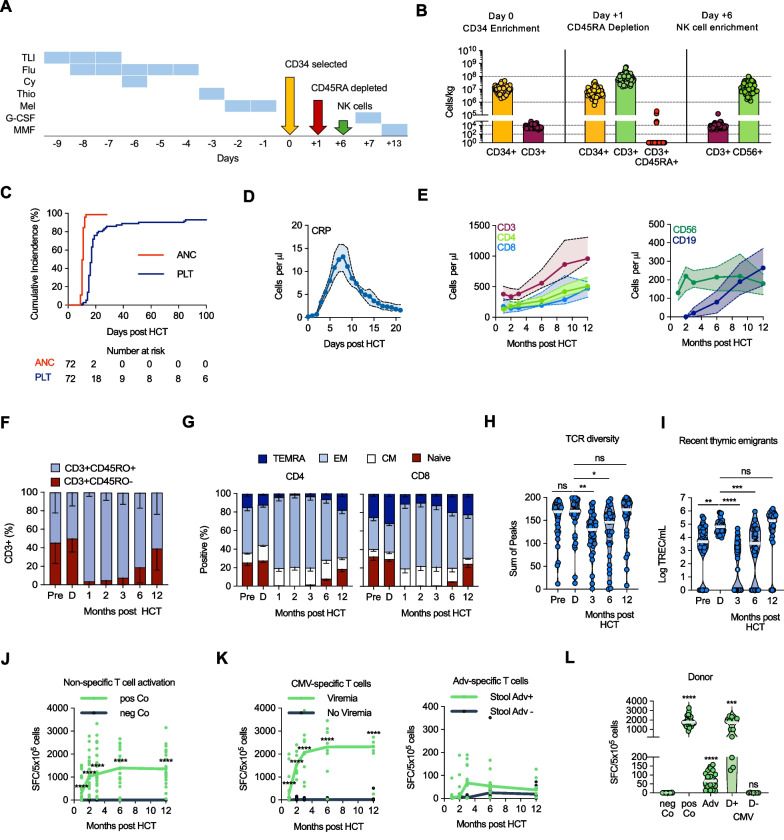
Transplant procedure, engraftment, and immune reconstitution. **A** Outline of transplant procedure. **B** Median cell doses of infused grafts. Values for CD3 + /CD45RA + cells that were ‘0’ were set to ‘1’ (n = 72). **C** Cumulative incidence of neutrophil and platelet engraftment (n = 72). **D** Median CRP levels during the first 21 days post-transplant, shaded area: CI 95% (n = 72). **E**–**K** Immune reconstitution in the first 12-months post-transplant. **E** Median T- (CD3, CD4, CD8), NK- (CD56), and B- (CD19) cell counts, shaded area: 95% CI (n = 69). **F**–**G** Percentage of (**F**) memory (CD3 + /CD45RO +) and naïve and TEMRA (CD3 + /CD45RO-) T-cell subsets (n = 64) and (**G**) naïve (CD45RO-/CCR7 +), CM (CD45RO + /CCR7 +), EM (CD45RO + /CCR7-), and TEMRA CD45RO-/CCR7-) T cells in CD4 and CD8 T-cell subsets in recipients pre- (n = 54–56) and post (n = 52–53) transplant and donors (n = 54–55). **H** TCR Vβ spectratyping (n = 38). **I** Recent thymic emigrants measured as T-cell receptor excision circles/mL (n = 38). Solid horizontal line is median in each violin plot in panels H, I. **J**–**K** Elispot assays post HCT, median and individual values are shown, data is plotted as SFC/5 × 10^5^. **J** Positive (SEB) and negative (DMSO) controls (n = 34). **K** Frequency of CMV-specific T cells with (n = 10) or without CMV viremia (n = 13) in CMV-seropositive/seropositive or -seropositive/ seronegative donor/recipient pairs, and Adv-specific T cells with (n = 13) or without (n = 13) Adv-positive stool samples. Statistical analysis for panels **H**–**K**: Two-way ANOVA, ns = not significant, *P < 0.05, **P < 0.01, ***P < 0.001, ****P < 0.0001. **L** Elispot assays of donors (n = 20), paired t-test (versus negative Co), ***P < 0.001, ****P < 0.0001. ANC, Absolute neutrophil count; PLT, Platelet; CRP, C-reactive protein; NK, natural killer; CM, central memory; EM, effector memory; TEMRA, Terminally differentiated effector memory; Pre, pre transplant recipients, D, donors, TREC, T-cell receptor excision circles; TCR, T-cell repertoire; CMV, cytomegalovirus; Co, control, pos, positive; neg, negative; SFC, spot-forming cells; SEB, Staphylococcal enterotoxin B; DSMO, dimethyl sulfoxide; D + , Donor positive; D −, Donor negative; Adv, Adenovirus

The preparative regimen consisted of 8 Gy total-lymphoid irradiation (TLI; four equal fractions), 30 mg/m^2^ fludarabine × 5 days, cyclophosphamide 60 mg/kg × 1 day, thiotepa 5 mg/kg × 2 on 1 day, and melphalan 70 mg/m^2^ × 2 days. The preparative regimen was based on previous institutional protocols using fludarabine, thiotepa and melphalan. As serotherapy was being omitted, cyclophosphamide was included for additional immune modulation and TLI was included to promote immunological tolerance and decrease risk of rejection. Patients received a G-CSF-mobilized CD34 + enriched HPC graft on day 0, and a second, CD45RA-depleted HPC graft on day 0/ + 1. On day + 6, they received a purified NK-cell infusion [32] from the same donor. G-CSF was started on day + 7. Sirolimus (n = 9) or mycophenolate mofetil (n = 63) was started 1 week following NK-cell infusion to stop before day + 60 (Fig. 1A).

### Correlative studies

Flow cytometry quantification of lymphocyte subsets, T-cell receptor excision circle (TREC), T-cell receptor (TCR) diversity by Vβ spectratyping, and enzyme-linked immunospot (Elispot) assays were performed on subsets of patients.

Details for transplant approaches and correlative studies are provided in Additional file 1: Supplementary Methods.

### Statistical analysis

This was a phase II clinical trial designed with Simon’s 2-stage optimum design [33] and an expansion cohort. The study included stopping rules for excessive rates of acute GVHD (aGVHD) and non-relapse mortality (NRM). The aGVHD stopping rule was triggered following accrual of 72 of the planned 75 subjects. This manuscript summarizes data as of April 2022 for the ‘full cohort’ and the subset of these patients in complete morphologic remission at the time of HCT as the ‘CR cohort’. The statistical analysis approach is described in Additional file 1: Supplementary Methods.

## Results

### Patient and donor characteristics

From June 6, 2013, to November 20, 2019, 81 pediatric patients with hematological malignancies were enrolled. The outcome of subsets of patients were previously reported [34–36]. Three patients did not proceed to transplant. Six patients received maraviroc for GVHD prophylaxis and were not included in the analysis (Additional file 1: Supplementary Methods, Supplementary Table 1). Seventy-two patients received protocol-specified therapy and are the focus of these analyses. Of the 72 evaluable patients (Table 1), 58% were males and the median age at transplant was 8 years (range: 0.6–20.8). Nineteen patients had active disease at time of transplantation and 53 patients were in morphologic CR (CR1: 25, ≥ CR2: 28). Amongst patients in morphologic CR, 20 (37.7%) had detectable disease, including 9 who were MRD-positive by flow cytometry. Of the donors, 38 were male with the majority being a parent. Most were mismatched at 4/8 HLA alleles, and 62 (86%) were NK alloreactive as defined by KIR receptor-ligand mismatch.

**Table 1:** Demographic, clinical, and donor characteristics of patients undergoing transplantation

Variable	Median (range) or n (%)	Total n
Patient age	8.1(0.6–20.8)	72
Patient gender		72
Male	42(58.3)	
Female	30(41.7)	
Race		72
White	52(72.2)	
Black	14(19.4)	
Other	6(8.4)	
Ethnicity		72
NOS Spanish, Hispanic, Latino	19(26.4)	
Non-Spanish speaking, non-Hispanic	53(73.6)	
Diagnosis		72
Myeloid	40(55.6)	40
AML	38(54.3)	
MDS	1(2.5)	
Myeloid Sarcoma	1(2.5)	
Lymphoid	30(41.7)	30
B ALL	24(80.0)	
T ALL	5(16.7)	
NHL	1(3.33)	
Biphenotypic	2(2.78)	2
Disease status		72
Active (> 5% blasts)	19(26.4)	19
PIF	10(52.6)	
Relapse	9(47.4)	
Complete Remission	53(73.6)	53
CR1	25(47.2)	
CR2	24(45.3)	
CR3 or more	4(7.55)	
MRD status (flow cytometry)		53
Positive	9(17.0)	
Negative	38(71.7)	
Unknown	6(11.3)	
Detectable disease (any)		53
Detectable (and known MRD)	20(37.7)	
Not detectable (and negative MRD)	27(50.9)	
Unkown MRD	6(11.3)	
CMV serostatus		72
CMV D-/R-	10(13.9)	
CMV D-/R +	12(16.7)	
CMV D + /R-	10(13.9)	
CMV D + /R +	40(55.6)	
HLA mismatch (n/8)		72
2	1(1.4)	
3	17(23.6)	
4	54(75.0)	
KIR Mismatch (rec-lig)		72
0	10(13.9)	
1	47(65.3)	
2	15(20.8)	
NK cell alloreactivity (rec-lig)		72
Yes	62(86.1)	
No	10(13.9)	
Donor KIR B score		71
0/1	53(74.6)	
2 or more	18(25.4)	
Donor age	37.0 (18–52)	72
Donor Gender		72
Male	38(52.8)	
Female	34(47.2)	
Donor relationship to patient		72
Father	35(48.6)	
Mother	31(43.1)	
Sibling	4(5.56)	
Other	2(2.78)	

NOS, not otherwise specified; AML, Acute Myeloid Leukemia; MDS, myelodysplastic syndrome; ALL, Acute lymphoblastic leukemia, NHL, Non-Hodgkin’s Lymphoma; PIF, Primary Induction Failure; CR, Complete Remission; MRD, Minimal Residual Disease; CMV, Cytomegalovirus; D, Donor; R, Recipient; HLA, Human Leukocyte Antigen; KIR, Killer Immunoglobulin Receptor; rec-lig, receptor-ligand; NK, Natural Killer

### Graft composition, engraftment and chimerism

After receiving protocol defined conditioning and both HPC grafts, 62 patients received NK-cell infusion on day + 6. Ten patients did not receive NK cells due to high-grade fevers and/or other complications. Figure 1B depicts HPC graft doses and Additional file 1: Supplementary Tables 2A, B summarize graft selection and depletion efficiencies. Seventy (97.2%) patients engrafted with a median time to neutrophil engraftment of 11 (9–13) days and platelet engraftment to > 50,000/μL of 17 (10–98) days (Fig. 1C). One patient experienced acute graft rejection, and another died prior to engraftment. Post-infusion, patients developed an inflammatory syndrome consisting of fevers and tachycardia which correlated with elevated serum C-reactive protein (CRP) levels that peaked at day + 8 (Fig. 1D). Thirty-two (44%) patients met CTCAE v3 criteria for engraftment/cytokine release/macrophage activation syndrome (Additional file 1: Supplementary Table 3). All engrafted patients had 100% donor chimerism at first chimerism evaluation performed soon after neutrophil engraftment, except 2 with 99% donor chimerism. Donor chimerism was 100% at day + 100 and at 1-year in all assessed patients, although several patients had intermittent mixed chimerism.

### Viral reactivation

Twenty-five patients developed CMV DNAemia with a cumulative incidence of 36.1% and 5 patients developed adenovirus (Adv) DNAemia with a cumulative incidence of 8.3% at 6 months. There were no cases of Epstein Barr Virus (EBV)-associated post-transplant lymphoproliferative disease (PTLD). Details of infections are listed in Additional file 1: Supplementary Table 3.

### Immune reconstitution

At 1-month post-transplant, the median CD3, CD4 and CD8 T-cell counts were 410/μL,140/μL and 200/μL (Fig. 1E). NK-and B-cell counts reached normal values at 1- and 3-months post-transplant (Fig. 1E). Of note, T-cell chimerism was not separately analyzed. For the first 6-months post-transplant, reconstituting T cells had a predominantly memory phenotype (Fig. 1F,G) and a diverse TCR repertoire (Fig. 1H), recapitulating graft content and highlighting the contribution of the infused memory T cells to the rapid immune reconstitution. Starting 3-months post-transplant, naïve T cells emerged, as confirmed by TREC analysis (Fig. 1I). Elispot assays revealed functional immune reconstitution as early as 1-month post-transplant (Fig. 1J) with detection of CMV-or Adv-specific T cells in patients with CMV reactivation or Adv-positive stool and/or blood samples (Fig. 1K). The presence of CMV- and Adv-specific T cells was confirmed in donors (Fig. 1L).

### Overall and event-free survival

**Fig. 2:**
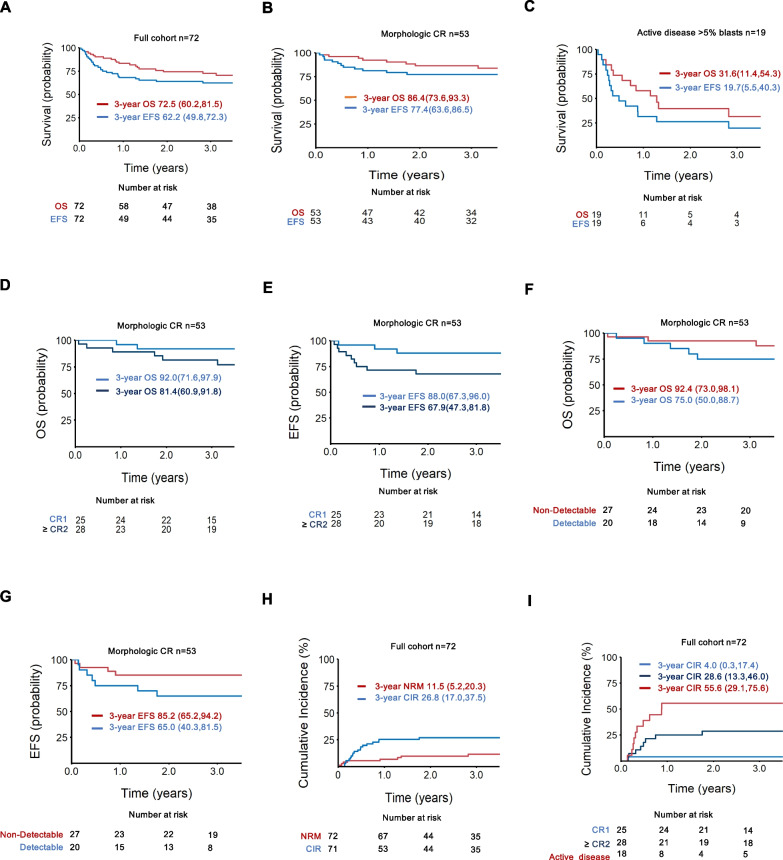
Relapse and survival. **A** Probability of OS and EFS for the full cohort. **B** Probability of OS and EFS for CR cohort. **C** Probability of OS and EFS for patients with active disease. **D** Probability of OS for patients in CR1, ≥ CR2. **E** Probability of EFS for patients in CR1, ≥ CR2. **F** Probability of OS for patients with non-detectible and detectible disease at time of transplant. **G** Probability of EFS for patients with non-detectible and detectible disease at time of transplant. (Fig F,G,- Six patients excluded due to unknown minimal residual disease (MRD) status) **H** Cumulative incidence of relapse and NRM for full cohort. **I** Cumulative incidence of relapse for patients in CR1, ≥ CR2, active disease (1 patient with active disease excluded due to death prior to engraftment). OS, overall survival; EFS, event-free survival; NRM, non-relapse mortality; CR, complete remission, CIR, cumulative incidence of relapse. 95% CI is listed in parenthesis

With a median follow-up (death or last contact) of 3.2 years among all evaluable patients, the 1- and 3-year OS was 83.2% (95% CI, 72.3–90.1) and 72.5% (95% CI, 60.2–81.5) with respective EFS (death or relapse) rates of 68.1% (95% CI, 56–77.5) and 62.2% (95% CI, 49.8–72.3) (Fig. 2A). Patients with Acute Myeloid Leukemia (AML) and Acute Lymphoblastic Leukemia (ALL) had comparable outcomes (*P* = 0.9949). The 3-year OS and EFS for the 53 patients in morphological CR were 86.4% (95% CI, 73.6–93.3) and 77.4% (95% CI, 63.6–86.5) (Fig. 2B), and 31.6% (11.4–54.3) and 19.7% (95% CI, 5.5–40.3) (Fig. 2C) respectively for patients with active disease. Among patients in morphological CR, the 3-year OS and EFS for patients in CR1 were 92.0% (95% CI, 71.6–97.9) and 88.0% (95% CI, 67.3–96.0) and for patients in ≥ CR2 were 81.4% (60.9–91.8) and 67.9% (95% CI, 47.3–81.8) (Fig. 2D,E). Amongst patients in CR1 and ≥ CR2 with no evidence of detectible disease (n = 27), the 3-year OS and EFS were 92.4% (95% CI, 73.0–98.1) and 85.2% (95% CI, 65.2–94.2), and for patients with evidence of any detectible disease (n = 20), including MRD positivity, 3-year OS and EFS were 75.0% (95% CI, 50.0–88.7) and 65.0% (95% CI, 40.3–81.5) (*P* = 0.1078, *P* = 0.0138) (Fig. 2F,G). There was however no difference in OS and EFS amongst patients transplanted with or without positive flow MRD (*P* = 0.4157 and *P* = 0.2992).

Univariate analyses for all patients (full cohort) and for patients in morphological CR (CR cohort) were performed. For the full cohort, disease status (CR) was associated with better OS (*P* < 0.0001) and higher graft CD3 T cell dose with worse OS (*P* = 0.046). In the CR cohort, cGVHD was associated with lower OS (*P* = 0.077) (Additional file 1: Supplementary Table 6). On univariate analysis for EFS in the full cohort, disease status (CR) and CMV serostatus (CMV-seronegative recipient) were associated with better EFS (*P* < 0.0001, *P* = 0.0514) and in the CR cohort, CMV serostatus (CMV-seronegative recipient) and increasing number of HLA mismatches (MM) were associated with better EFS (*P* = 0.0454, *P* = 0.0594) (Table 2). In multivariate analysis for OS and EFS, only disease status for the full cohort remained significant (*P* = 0.0004) (Table 3).

**Table 2:** Selected univariable analysis results*

Characteristic	Full Cohort (n = 72)					CR Cohort (n = 53)				
	N	Events	1-year OS (95% CI)	3-year OS (95% CI)	*P*	N	Events	1-year OS (95% CI)	3-year OS (95% CI)	*P*
**Overall Survival (OS)**										
Disease phase					** < .0001**					0.5634
CR1	25	4	96.0(74.8,99.4)	92.0(71.6,97.9)		25	4	96.0(74.8,99.4)	92.0(71.6,97.9)	
CR2 or more	28	6	89.1(70.0,96.4)	81.4(60.9,91.8)		28	6	89.1(70.0,96.4)	81.4(60.9,91.8)	
Active disease	19	12	57.9(33.2,76.3)	31.6(11.4,54.3)		0				
CD3 dose					**0.0462**					0.2943
16.08–45.0	24	4	91.7(70.6,97.8)	83.3(61.5,93.4)		20	2	100	90.0(65.6,97.4)	
45.34–91.1	24	6	79.2(57.0,90.8)	74.5(51.7,87.7)		17	3	88.2(60.6,96.9)	82.4(54.7,93.9)	
91.98–528.5	24	12	78.3(55.4,90.4)	59.1(35.8,76.4)		16	5	86.2(55.0,96.4)	86.2(55.0,96.4)	
Donor Gender					0.0953					0.5149
Male	38	8	92.0(77.1,97.3)	83.1(66.0,92.0)		33	5	97.0(80.4,99.6)	90.5(73.4,96.8)	
Female	34	14	73.5(55.3,85.3)	61.5(43.0,75.5)		20	5	85.0(60.4,94.9)	80.0(55.1,92.0)	

	N	Events	1-year EFS (95% CI)	3-year EFS (95% CI)	*P*	N	Events	1-year EFS (95% CI)	3-year EFS (95% CI)	*P*
**Event-Free Survival (EFS)**										
Disease phase					** < .0001**					0.1904
CR1	25	5	92.0(71.6,97.9)	88.0(67.3,96.0)		25	5	92.0(71.6,97.9)	88.0(67.3,96.0)	
CR2 or more	28	10	71.4(50.9,84.6)	67.9(47.3,81.8)		28	10	71.4(50.9,84.6)	67.9(47.3,81.8)	
Active disease	19	15	31.6(12.9,52.2)	19.7(5.5,40.3)		0				
CMV Serostatus					**0.0046**					0.1699
D-/R-	10	2	90.0(47.3,98.5)	90.0(47.3,98.5)		10	2	90.0(47.3,98.5)	90.0(47.3,98.5)	
D-/R +	12	9	33.3(10.3,58.8)	33.3(10.3,58.8)		6	3	66.7(19.5,90.4)	66.7(19.5,90.4)	
D + /R-	10	3	80.0(40.9,94.6)	68.6(30.5,88.7)		7	0	100	100	
D + /R +	40	16	70.0(53.3,81.7)	62.5(45.7,75.4)		30	10	76.7(57.2,88.1)	70.0(50.3,83.1)	
CMV Recipient status					0.0514					**0.0454**
Positive	52	25	61.5(47.0,73.2)	55.8(41.3,68.0)		36	13	75.0(57.5,86.1)	69.4(51.7,81.8)	
Negative	20	5	85.0(60.4,94.9)	79.7(54.5,91.9)		17	2	94.1(65.0,99.1)	94.1(65.0,99.1)	
HLA MM					0.3639					0.0594
2	1	1	0	0		1	1	0	0	
3	17	8	64.7(37.7,82.3)	58.8(32.5,77.8)		13	5	76.9(44.2,91.9)	69.2(37.3,87.2)	
4	54	21	70.4(56.3,80.7)	64.4(50.0,75.7)		39	9	84.6(68.9,92.8)	82.1(66.0,91.0)	

	N	Events	1-year NRM (95% CI)	3-year NRM (95% CI)	*P*	N	Events	1-year NRM (95% CI)	3-year NRM (95% CI)	*P*
**Cumulative Incidence of Non-Relapse Mortality (NRM)**										
Donor Gender					**0.0064**					0.0622
Male	38	1	0	0		33	1	0	0	
Female	34	9	14.7(5.3,28.7)	23.9(11.0,39.4)		20	4	10.0(1.6,27.8)	15.0(3.5,34.1)	
Donor relationship					**0.0273**					0.1831
Father	35	1	0	0		30	1	0	0	
Mother	31	9	16.1(5.7,31.2)	26.0(12.0,42.5)		18	4	11.1(1.7,30.4)	16.7(3.9,37.3)	
Sibling	4	0	0	0		3	0	0	0	
Other	2	0	0	0		2	0	0	0	
CD3 dose					**0.0326**					0.2173
16.08–45.0	24	0	0	0		20	0	0	0	
45.34–91.1	24	3	8.3(1.4,23.7)	12.5(3.0,29.1)		17	2	5.9(0.3,24.2)	11.8(1.8,31.9)	
91.98–528.5	24	7	12.5(3.0,29.1)	21.2(7.4,39.8)		16	3	6.3(0.3,25.8)	6.3(0.3,25.8)	

	N	Events	1-year CIR (95% CI)	3-year CIR (95% CI)	*P*	N	Events	1-year CIR (95% CI)	3-year CIR (95% CI)	*P*
Cumulative Incidence of Relapse (CIR)**										
Disease phase					**0.0012**					**0.0114**
CR1	25	1	4.0(0.3,17.4)	4.0(0.3,17.4)		25	1	4.0(0.3,17.4)	4.0(0.3,17.4)	
CR2 or more	28	9	25.0(10.8,42.2)	28.6(13.3,46.0)		28	9	25.0(10.8,42.2)	28.6(13.3,46.0)	
Active disease	18	10	55.6(29.1,75.6)	55.6(29.1,75.6)						
CMV Serostatus					**0.011**					0.1222
D-/R-	10	0	0	0		10	0	0	0	
D-/R +	12	7	58.3(24.4,81.4)	58.3(24.4,81.4)		6	2	33.3(3.2,70.4)	33.3(3.2,70.4)	
D + /R-	10	2	20.0(2.6,49.0)	20.0(2.6,49.0)		7	0	0	0	
D + /R +	39	11	23.1(11.3,37.3)	25.6(13.2,40.1)		30	8	20.0(7.9,36.0)	23.3(10.1,39.7)	
CMV Recipient status					**0.0357**					**0.0172**
Positive	51	18	31.4(19.2,44.3)	33.3(20.8,46.4)		36	10	22.2(10.3,37.0)	25.0(12.3,40.0)	
Negative	20	2	10.0(1.6,27.8)	10.0(1.6,27.8)		17	0	0	0	
HLA MM					0.09					**0.0236**
2	1	1	100	100		1	1	100	100	
3	17	7	35.3(13.8,57.8)	41.2(17.8,63.4)		13	4	23.1(5.1,48.5)	30.8(8.8,56.5)	
4	53	12	20.8(11.0,32.6)	20.8(11.0,32.6)		39	5	10.3(3.2,22.2)	10.3(3.2,22.2)	

	N	Events	1-year G 3/4 aGVHD (95% CI)	3-year G 3/4 aGVHD (95% CI)	*P*-value	N	Events	1-year G 3/4 aGVHD (95% CI)	3-year G 3/4 aGVHD (95% CI)	*P*-value
**Cumulative Incidence of Grade III-IV aGVHD**										
NK alloreactivity					**0.004**					**0.0066**
Yes	62	14	22.6(13.1,33.7)	22.6(13.1,33.7)		45	10	22.2(11.4,35.3)	22.2(11.4,35.3)	
No	10	7	70.0(28.2,90.4)	70.0(28.2,90.4)		8	6	75.0(23.2,94.5)	75.0(23.2,94.5)	
KIR MM (rec-lig)					**0.0051**					**0.0142**
0	10	7	70.0(28.2,90.4)	70.0(28.2,90.4)		8	6	75.0(23.2,94.5)	75.0(23.2,94.5)	
1	47	13	27.7(15.7,41.0)	27.7(15.7,41.0)		34	9	26.5(13.0,42.1)	26.5(13.0,42.1)	
2	15	1	6.7(0.4,26.9)	6.7(0.4,26.9)		11	1	9.1(0.4,34.7)	9.1(0.4,34.7)	
Donor KIR B score					**0.0257**					**0.006**
0	23	6	26.1(10.3,45.2)	26.1(10.3,45.2)		17	5	29.4(10.2,51.9)	29.4(10.2,51.9)	
1	30	5	16.7(5.9,32.1)	16.7(5.9,32.1)		18	1	5.6(0.3,23.1)	5.6(0.3,23.1)	
2	14	8	57.1(26.6,78.9)	57.1(26.6,78.9)		14	8	57.1(26.6,78.9)	57.1(26.6,78.9)	
3	4	2	50.0(2.3,88.1)	50.0(2.3,88.1)		3	2	66.7(0.2,97.3)	66.7(0.2,97.3)	

	N	Events	1-year MS cGVHD (95% CI)	3-year MS cGVHD (95% CI)	*P*	N	Events	1-year MS cGVHD (95% CI)	3-year MS cGVHD (95% CI)	*P*
**Cumulative Incidence of Moderate-Severe cGVHD**										
Donor Gender					0.067					**0.0166**
Male	38	3	8.2(2.1,20.0)	8.2(2.1,20.0)		33	3	9.5(2.4,22.8)	9.5(2.4,22.8)	
Female	34	8	20.6(8.9,35.6)	23.5(10.9,38.9)		20	7	35.0(15.1,55.8)	35.0(15.1,55.8)	
Donor relationship					0.1328					**0.0347**
Father	35	2	6.0(1.0,17.7)	6.0(1.0,17.7)		30	2	7.0(1.2,20.4)	7.0(1.2,20.4)	
Mother	31	8	22.6(9.7,38.7)	25.8(11.9,42.2)		18	7	38.9(16.8,60.7)	38.9(16.8,60.7)	
Sibling	4	1	25.0(0.3,71.4)	25.0(0.3,71.4)		3	1	33.3(0.1,83.2)	33.3(0.1,83.2)	
Other	2	0	0	0		2	0	0	0	

Bold values that show signficance with *p*-value < 0.01

CR, Complete Remission; aGVHD; acute graft versus host disease; cGVHD, chronic graft versus host disease; CMV, Cytomegalovirus; R, Recipient; D; Donor, NK, Natural Killer; HLA, Human Leukocyte Antigen; MM, mismatch; KIR, Killer Immunoglobulin Receptor; rec-lig, receptor-ligand

^*^Two-sided p-values are reported from the logrank (OS, EFS) and Gray’s (NRM, CIR, aGVHD, cGVHD) tests

^**^Excludes 1 patient with active disease at the time of HCT, as they died prior to engraftment and not at risk of relapse

**Table 3:** Multivariable model results*

Full Cohort (n = 72)			CR Cohort (n = 53)		
Covariate	Adjusted HR (95% CI)	*P*	Covariate	Adjusted HR (95% CI)	*P*
**Overall Survival (OS)**					
CR	0.25 (0.09, 0.67)	**0.0058**	CR2 or more	1.83 (0.48, 6.99)	0.3779
CD3 content in graft (tertile)	1.74 (0.97, 3.11)	0.0624	CD3 content in graft (tertile)	1.86 (0.84, 4.13)	0.1285
Mother donor	1.36 (0.48, 3.82)	0.5622			
aGVHD	1.17 (0.48, 2.87)	0.7306	aGVHD	2.39 (0.63, 9.14)	0.2026
cGVHD	1.02 (0.38, 2.74)	0.9722	cGVHD	1.75 (0.44, 6.88)	0.4250
CMV Rec Positive	1.72 (0.55, 5.42)	0.3554	CMV Rec Positive	3.49 (0.65, 18.64)	0.1431
**Event Free Survival (EFS)**					
CR	0.26 (0.12, 0.54)	**0.0004**	CR2 or more	2.04 (0.65, 6.38)	0.2191
CMV Rec Positive	1.79 (0.66, 4.84)	0.2527	CMV Rec Positive	3.52 (0.73, 17.04)	0.1180
aGVHD	0.87 (0.39, 1.97)	0.7393	aGVHD	1.37 (0.47, 4)	0.5689
CGVHD	0.81 (0.34, 1.94)	0.6403	cGVHD	1.08 (0.33, 3.48)	0.9012
NK Alloreactive	2.07 (0.45, 9.48)	0.3485			
			2 or 3 HLA MM	1.57 (0.52, 4.73)	0.4230
**Non-relapse mortality (NRM)**					
CD3 content in graft (tertile)	3.53 (1.41, 8.8)	**0.0068**			
Mother donor	11.46 (1.64, 80.27)	**0.0140**	Mother donor	7.69 (1.75, 33.76)	**0.0069**
aGVHD	1.29 (0.41, 4.06)	0.6700	aGVHD	3.2 (0.44, 23.36)	0.2500
cGVHD	1.25 (0.36, 4.37)	0.7300	cGVHD	3.57 (0.41, 31.28)	0.2500
Relapse**					
CR	0.35 (0.13, 0.92)	**0.0330**	CR2 or more	4.08 (0.87, 39.8)	0.0773
CMV Rec Positive	3.14 (0.8, 12.23)	0.1000	CMV Rec Positive	9.92 (1.14, 1299.27)	**0.0347**
cGVHD	0.33 (0.08, 1.49)	0.1500	cGVHD	0.61 (0.06, 2.98)	0.5762
aGVHD	0.62 (0.18, 2.1)	0.4400	aGVHD	1.24 (0.3, 4.24)	0.7424
NK Alloreactive	1.8 (0.32, 10.14)	0.5100	NK Alloreactive	1.68 (0.11, 243.6)	0.7375
			2 or 3 HLA MM	1.42 (0.41, 4.95)	0.5693
**Grade III-IV aGVHD**					
NK Alloreactive	0.34 (0.14, 0.83)	**0.0180**	NK Alloreactive	0.32 (0.13, 0.81)	**0.0160**
CD3 content in graft (tertile)	1.13 (0.67, 1.9)	0.6600	CD3 content in graft (tertile)	1.12 (0.64, 1.95)	0.7000
CD45RA content in graft (tertile)	1.39 (0.73, 2.66)	0.3200	CD45RA content in graft (tertile)	1.77 (0.84, 3.72)	0.1300
**Moderate-Severe cGVHD**					
			Mother donor	6.11 (1.77, 21.05)	**0.0041**
			CD3 content in graft (tertile)	1.46 (0.69, 3.1)	0.3200
			CD45RA content in graft (tertile)	1.4 (0.61, 3.23)	0.4300

Bold values that show signficance with *p*-value < 0.01

^*^Model specifications differ by cohort and by outcome (details in the Additional file 1: Supplementary Materials); two-sided *P*-values are reported

^**^Excludes 1 patient with active disease at the time of HCT, as they died prior to engraftment and not at risk of relapse

CR, Complete Remission; aGVHD; acute graft versus host disease; cGVHD, chronic graft vesus host disease; CMV, Cytomegalovirus; R, Recipient; NK, Natural Killer; HLA, Human Leukocyte Antigen; MM, mismatch; KIR, Killer Immunoglobulin Receptor; rec-lig, receptor-ligand

### Incidence and predictors of relapse

The 3-year cumulative incidence of relapse (CIR) for all patients was 26.8% (95% CI, 17.0–37.5) (Fig. 2H) with no differences between patients with ALL and AML. It varied based on disease status (CR1: 4.0% (0.3–17.4), ≥ CR2: 28.6% (95% CI, 13.3–46.0), active disease: 55.6% (95% CI, 29.1–75.6)) (Fig. 2I). Amongst patients with morphologic CR, the 3-year CIR was lower for patients without versus with detectable disease (7.4% (95% CI, 1.2–21.4) versus 30.0% (95% CI, 11.8–50.7).

Univariate analysis for the full cohort showed being in CR and being a CMV-seronegative recipient regardless of CMV-donor status were significantly associated with lower relapse risk (*P* = 0.0012, *P* = 0.0357). In the CR cohort, CR1, recipient CMV seronegativity, and increasing number of HLA mismatches were significant for lower risk of relapse (*P* = 0.0114, *P* = 0.0172, *P* = 0.0236) (Table 2). On multivariate analysis, only disease status remained significant (*P* = 0.0330) for the full cohort. In the CR cohort, recipients that were CMV-seronegative had the lowest rate of relapse (0/17) (*P* = 0.0347) but as ‘zero’ patients experienced relapse, additional statistical modeling needed to be performed (Additional file 1: Supplementary Methods) (Table 3).

### GVHD: Incidence and outcome

**Fig. 3:**
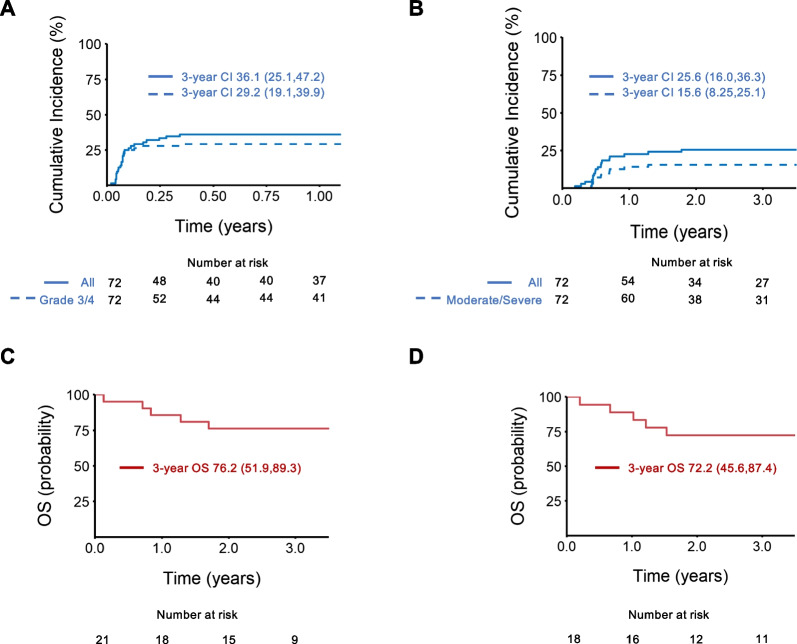
GVHD and survival. **A** Cumulative incidence of all aGVHD and grade III-IV aGVHD. **B** Cumulative incidence of all cGVHD and moderate-severe cGVHD. **C** Probability of OS from onset of grade III-IV aGVHD. **D** Probability of OS from onset of cGVHD. aGVHD, acute graft-versus-host disease; cGVHD, chronic graft-versus-host disease, OS, overall survival. 95% CI is listed in parenthesis

The cumulative incidence of any aGVHD was 33.3% (95% CI, 22.7–44.3) at day + 100 and 36.1% (95% CI, 25.1–47.2) at 1-year. The incidence of grade II-IV and III/IV aGVHD was 33.3% (95% CI, 22.7,44.3) and 29.2% (95% CI, 19.1,39.9) at 1-year (Fig. 3A). Twenty-six patients developed aGVHD with gut (73%), skin (54%), and/or liver (12%) involvement. aGVHD treatment included systemic steroids (23/26), infliximab (10/26), tacrolimus (10/26) or ruxolitinib (1/26). The median duration of systemic steroid use was 51.5 days (range 3–149 days) (Additional file 1: Supplementary Table 4A). aGVHD resolved in the majority (23/26) of patients, with 1 patient having ongoing aGVHD at time of death and 2 patients with ongoing aGVHD at time of relapse (Additional file 1: Supplementary Table 4B). On univariate analysis, presence of NK alloreactivity as determined by KIR mismatch, increasing number of KIR mismatches, and lower donor KIR B haplotype score were associated with lower risk of grade III/IV aGVHD for the full and CR cohorts (Table 2). NK alloreactivity remained significant (*P* = 0.0180) in multivariate analysis for the full and CR cohorts (Table 3).

The cumulative incidence of any cGVHD was 25.6% (95% CI, 16.0–36.3) and moderate-severe cGVHD was 15.6% (95% CI, 8.3–25.1) at 3-years (Fig. 3B). aGVHD preceded cGVHD in < 50% of cases (7/18). On univariable analysis (Table 2) for the full cohort, there were no factors associated with moderate/severe cGVHD. In the CR cohort, recipients of maternal donors had a higher risk of cGVHD on univariate (Table 2) and multivariate analyses (Table 3). OS for patients with grade III-IV aGVHD was 85.7% at 1-year from aGVHD onset (Fig. 3C). The 1- and 3-year OS for patients from onset of developing cGVHD was 88.9%. and 72.2% respectively (Fig. 3D).

### Cumulative incidence of Non-Relapse Mortality

The 3-year cumulative incidence of NRM for the entire cohort was 11.5% (95% CI, 5.2–20.3) (Fig. 2H, Additional file 1: Supplementary Table 5). The incidence of NRM was higher in patients with active disease (5/19) compared to patients in morphologic CR (5/53), and 6/10 deaths were in patients with AML. Four deaths occurred early (< 100 days) due to organ toxicities, and of the 6 deaths that occurred > 100 days, 4 were attributable to cGVHD and 1 patient had recently completed therapy for cGVHD. On univariate analysis, in the full cohort, having female donor, highest tertile CD3 count in the infused graft, and occurrence of cGVHD were associated with worse NRM; in the CR cohort, worse NRM was associated with having a female donor and presence of cGVHD (Table 2, Additional file 1: Supplementary Table 6). On multivariate analysis, high CD3 + dose in the graft and maternal donor remained significant for worse NRM in the full cohort. Only maternal donor was significant for worse NRM for the CR cohort (Table 3).

## Discussion

In this prospective clinical trial, pediatric patients with high-risk hematological malignancies received a sub-myeloablative, TBI- and serotherapy-free conditioning regimen followed by CD34-enriched and CD45RA-depleted haploidentical donor grafts and NK-cell addback. This regimen was associated with rapid, robust immune reconstitution and low rates of graft rejection, viral reactivation, and relapse. The EFS using our approach compares favorably to other transplant strategies using matched donors, despite the inclusion of patients with MRD and while using a sub-myeloablative, TBI free approach. While the incidence of GVHD was higher than observed with more stringent T-cell depleted grafts, most patients recovered rapidly with therapy, resulting in promising long-term outcomes in this high-risk population. Importantly, modifiable factors were identified to mitigate the risk of developing GVHD in subsequent trials. Finally, as these results were obtained using haploidentical donors for patients with no other suitable donor choices, our strategy could provide excellent outcomes for all pediatric patients in need of an HCT.

Despite using an ex-vivo T-cell depletion strategy, the avoidance of serotherapy along with the large number of infused CD34 + and CD45RA-depleted cells led to rapid immune reconstitution with mean CD3 and CD4 T-cell counts of 400 cells/μL and 140 cells/μL at day + 30. These T cells had a predominantly memory phenotype suggesting adoptive transfer from the infused graft. In contrast, other T-cell depletion approaches such as TCRαβ depletion, achieve comparable T-cell counts only at month 6 post-transplant [37]. The early reconstitution is clinically relevant as CD4 counts of ≥ 50 cells/μL at day + 100 are associated with improved survival [38, 39].

This robust immune reconstitution translated to low rates of viral infection. The cumulative incidence of CMV DNAemia (36.1%) compares favorably to other studies, including for recipients of T-replete grafts [40–42]. The cumulative incidence of Adv DNAemia (8.3%) was lower than in other pediatric studies that report incidence of 10–26% and a mortality rate of up to 50% [43–47]. Despite the use of a T-deplete graft the incidence of EBV-PTLD was 0% without the use of prophylactic Rituximab or CD19-depletion of the graft. This suggests that the infused donor memory T cells not only reconstituted rapidly but retained their ability to respond to viral infections, a conclusion corroborated by our Elispot results demonstrating functional virus-specific T cells as early as 1-month post-transplant.

The 3-year EFS for patients in morphologic CR including those with MRD positive and detectable disease was 77.4% and for those with flow MRD-negative CR was 85.2%. This compares favorably to other T-cell depletion approaches, including TCRαβ or CD45RA-depletion [37, 48, 49] despite the use of a sub-myeloablative TBI-free conditioning regimen in our cohort. In contrast, other studies including a large trial using TCRαβ depletion and the recent randomized FORUM trial, show that the use of TBI is required to maintain low relapse rates for patients with ALL [37, 48, 50]. Further, patients with or without pre-HCT MRD positivity in our cohort had no difference in EFS despite several prior studies showing negative impact of pre-HCT MRD on EFS [37, 51–53]. Our outcome suggests that robust reconstitution with multiple immune effectors provides a potent GVL effect and enables the use of sub-myeloablative, TBI-free conditioning regimens without compromising EFS in our high-risk population.

Active disease (> 5% blasts) at time of transplant and decreasing number of HLA mismatches were associated with higher rates of relapse, with the latter suggesting that the GVL effect could be enhanced by selecting a full haplotype mismatched donor. Intriguingly, CMV-seronegative recipients had low rates of relapse. Most previous studies have evaluated effect of CMV reactivation (not serostatus) on leukemic relapse [54–57] and whether or not there is a protective effect remains controversial [54, 55, 58]. However, one large study evaluated serostatus and showed higher relapse rates in patients transplanted with CMV-seropositive matched unrelated donors [41]. The mechanism of protection from relapse in CMV-seronegative recipients in our study remains elusive and warrants further exploration particularly in the context of early NK-cell addback.

Compared to more stringent T-cell depletion approaches, the rates of aGVHD and cGVHD were higher in our study. This could be due to the very high absolute number of T cells infused in the graft. In clinical trials, the median number of haploidentical T cells infused with the use of TCRαβ depletion is 10^4^ T cells/kg [48, 59] and with CD45RA-depletion in the matched related and unrelated donor setting, is 10^7^ T cells/kg [49]. We infused a median haploidentical T-cell dose of 6.08 × 10^7^/kg. The predominant memory T-cell content in the infused graft likely made large doses of infused haploidentical T cells tolerable as > 10^5^ unmanipulated haploidentical T cells/kg cause GVHD. As reported by others [49], aGVHD in our study was responsive to therapy and required systemic steroids for a median of < 2 months, confirming preclinical studies that have highlighted differences between aGVHD induced by naïve and memory T cells [60, 61]. In addition, we used a short course of sirolimus or mycophenolate mofetil as GVHD prophylaxis. However, other approaches could be explored in the future such as the use of abatacept, which has the added advantage of preserving NK-cell function [62–64].

The cumulative incidence of aGVHD was substantially higher in patients who received transplants from non-NK-cell alloreactive compared to NK-cell alloreactive donors (70% vs 22.6%). Intriguingly, the impact was differentially increased with increasing number of KIR mismatches. Altogether, we found that NK-alloreactive donor, increasing KIR mismatches and lower donor KIR B haplotype content, were each associated with significantly lower rates of grade III/IV aGVHD but not cGVHD. Pre-clinical and clinical studies have shown that high numbers of alloreactive NK cells can decrease the occurrence of aGVHD [11, 65–67] by depleting antigen-presenting cells [11, 68] or activated T cells [69, 70], and by controlling autologous T-cell responses [71, 72], potentially explaining our findings. Similar clinical findings have been reported by other groups that utilized more stringent T-cell depletion techniques [11]. However, the number of recipients of transplants from non- NK-cell alloreactive donors in our study was small, and future studies are needed to explore the relationship between NK-cell alloreactivity and aGVHD in larger cohorts receiving CD45RA-depleted transplants.

The use of maternal donors in our study was associated with development of cGVHD but not aGVHD. There have been conflicting reports with occurrence of cGVHD and use of maternal donors and while others have reported similar findings [42, 73, 74], the relationship between maternal donors and GVHD is dependent on transplant platform utilized [42] and in our context could be due to sensitization of maternal donors to unshared HLA during pregnancies with memory T cells retained in the infused graft. While not statistically significant, a higher CD3 cell dose in the graft was associated with higher rates of cGVHD.

NRM in our cohort, particularly for patients in CR was low (5.7%) and compares favorably with other pediatric transplant studies [42, 51, 75]. The majority of NRM occurred in patients with active disease, where high CD3 cell dose in the graft was associated with early NRM due to organ toxicities. In patients in CR, NRM occurred late and was associated with mother as donor, which was related to development of cGVHD.

There are some limitations to our approach. The cost and complexity of ex vivo graft manipulation in its current form may limit its implementation to centers. The apheresis procedures required in our study were more extensive than for standard transplantation but were well tolerated and feasible as majority of donors were parents or family members who were highly motivated. Modifications to simplify the process to one cell infusion of a CD45RA-depleted product [76], instead of two infusions will make our approach more accessible. Likewise, the use of fully automated, closed system selection devices hold the promise of simplifying the process. While other haploidentical transplant approaches such as use of T-replete grafts and post-transplant cyclophosphamide are easier and less expensive, only ex vivo graft manipulation enables the selective infusion of immune effector cell subsets to study their contribution to transplant outcomes. We believe that studies like ours are critical to develop approaches to enhance GVL effects in the setting of reduced toxicity conditioning regimens to achieve comparable outcomes to standard transplant platforms with the ultimate goal to separate GVL effects from GVHD with minimal exposure to toxic chemo-radiotherapies.

In summary, our study using multiple immune effectors derived from haploidentical donors following a sub-myeloablative, non-TBI based regimen was associated with excellent outcomes in pediatric patients with high-risk hematological malignancies. The use of NK-alloreactive donors, avoiding maternal donors, and lowering the infused CD3 dose has the potential to mitigate the risk of GVHD in future studies. In addition, understanding the biological mechanism of relapse protection in CMV-seronegative recipients could lead to further refinement of our approach.

### Supplementary Information

**Additional file 1**. Supplementary materials.

## Data Availability

The datasets used and/or analyzed during the current study are available from the corresponding author on reasonable request.

## Abbreviations

HCT: Hematopoietic cell transplantation
GVL: Graft-versus-leukemia
TBI: Total body irradiation
NK: Natural killer
GVHD: Graft-versus-host disease
KIR: Killer immunoglobulin-like receptors
HPC: Hematopoietic progenitor cell
HLA: Human Leukocyte Antigen
CR: Complete remission
MRD: Minimal residual disease
TLI: Total-lymphoid irradiation
TREC: T-cell receptor excision circle
TCR: T-cell receptor
Elispot: Enzyme-linked immunospot
aGVHD: Acute GVHD
cGVHD: Chronic GVHD
NRM: Non-relapse mortality
CRP: C-reactive protein
CMV: Cytomegalovirus
Adv: Adenovirus
EBV: Epstein Barr Virus
PTLD: Post-transplant lymphoproliferative disease
AML: Acute Myeloid Leukemia
ALL: Acute Lymphoblastic Leukemia
CIR: Cumulative incidence of relapse
