# Supplementary material for: Memory T-cell enriched haploidentical transplantation with NK cell addback results in promising long-term outcomes: a phase II trial
Source: J Hematol Oncol. 2024 Jun 27;17:50. doi: 10.1186/s13045-024-01567-0 (PMC11212178; doi:10.1186/s13045-024-01567-0)
Supplement: Supplementary file 1 — Additional file 1. Supplementary materials. [file 13045_2024_1567_MOESM1_ESM.pdf]

Supplementary materials

Supplemental methods

Supplemental tables

- Table 1: Outcome in maraviroc patients
- Table 2: Graft depletion and recovery
- Table 3: Common and notable adverse events
- Table 4: Acute GVHD meds used and outcomes
- Table 5: Cause of death
- Table 6: Complete univariate analysis

HAPNK1 protocol

SUPPLEMENTARY METHODS

KIR typing and selection of alloreactive donors

KIR genotyping was performed by sequence specific primers (SSP) (Olerup KIR, CareDx, Brisbane, CA). NK alloreactivity was determined by “missing self” or KIR receptor-ligand mismatch, i.e. inhibitory KIR gene(s) present in the donor for which there is no KIR ligand (class I HLA) in the patient[1]. KIR-HLA pairs assessed were: KIR2DL1- HLA C2 group alleles, KIR2DL2/2DL3- HLA C1 group alleles, and KIR3DL1- HLA Bw4+ alleles. The HLA-C groups are defined by amino acid 80 of the α1 helix; HLA C1 group comprising alleles expressing lysine, and HLA C2 group as HLA-C alleles expressing asparagine at this position. The HLA C2 group also includes B*46:01 and B*73:01 alleles. HLA alleles encoding the Bw4 serological epitope include many HLA-B and some HLA-A alleles. Bw4+ exceptions are B*13 and A*25, which do not efficiently bind or inhibit KIR3DL1-expressing NK cells[2].KIR ligand-ligand mismatch and donor KIR B haplotype content were calculated as previously described[3,4]

HPC collection and processing

HPCs were obtained via G-CSF mobilization of the haploidentical donor and collection by leukapheresis on day 5 and 6 of G-CSF. All processing was performed in the Human Application Laboratory of St. Jude Children’s Research Hospital. The first HPC product, collected on day 5, was T-cell depleted using the CliniMACS device and CD34 Microbead (Miltenyi Biotec, Auburn, CA, USA). Minimum cell dose required for the CD34+ enriched progenitor cell graft was $2x10^6$ CD34+ cells/kg. Maximum CD3+ dose allowed for the CD34+ enriched HPC was $0.1x10^6$ CD3+ cells/kg. The HPC product collected on day 6 was processed for CD45RA+ cell depletion using the CliniMACS device and its ‘Depletion 3.1’ software. There was no target CD34+ dose or CD3+ dose on the CD45RA+ depleted product; however, release criteria of the product included a ⩾2 log10 depletion of CD45RA+ cells, and a maximum CD3+CD45RA+ dose of $0.05x10^6$ cells/kg. The NK cell product was collected by leukapheresis 5 days after the second HPC collection as a non-mobilized product and was processed on the CliniMACS device as previously described[5]. All three cell products were infused fresh.

Supportive care

Patients were monitored and received standard supportive care post-transplant, including prophylaxis against *Pneumocystis jirovecii* infection with Trimethoprim/sulfamethoxazole and antifungal prophylaxis wtith micafungin/voriconazole per institutional SOPs. Antiviral prophylaxis was initially acyclovir (n=37) and was later changed to once daily valganciclovir (n=35) due to concerns that cytomegalovirus (CMV), and/or Human Herpes Virus 6 (HHV6) antigen load was partially driving pre-engraftment CRS.

Adverse events

All NCI grade III-V adverse events from the start of conditioning and throughout the first-year post HCT were recorded as per CTCAE version 3 regardless of their relationship to the treatment. Additionally, NCI Grade I-II adverse events considered clinically significant and related to the treatment were also recorded (e.g. VOD, GVHD, hemorrhagic cystitis).

Viremia testing

Recipients underwent CLIA-approved PCR testing at least weekly for the first 100 days post-HCT for CMV, Epstein-Barr Virus (EBV), and Adenovirus (Adv), regardless of clinical symptoms. Patients with diarrhea underwent PCR testing for Adv in the stool. Starting in 2018, asymptomatic screening for AdV in stool was done pre-transplant. Those with positive screening were followed with weekly AdV PCR in stool until 2 consecutive negative results. Patients received antiviral therapy if viral load was detected above >6 Log10 irrespective of viremia. Patients with hemorrhagic cystitis received PCR testing for BK virus in blood and urine. Patients with fever and maculopapular rash underwent PCR testing for HHV6 in blood.

Flow cytometry

Flow cytometry analysis was performed to quantify lymphocyte subsets on a BD LSR II flow cytometer with BD FACS DIVA software (BD Biosciences, Heidelberg, Germany). The following antibodies were used for phenotypic analysis: CD45-FITC, CD19/20-PE, CD14-Cy-7, CD25-FITC, CD127-PE, CD45RA-APC, CD3-APC Cy-7, CD8-FITC, CD4-PE, CD4-APC Cy7, CD8-APC Cy7, CD62L-FITC, CCR7-PE, CD27 FITC, CD62L-PE, (Beckton Dickinson, Franklin Lakes, NJ, USA), CD3-ECD, CD56-APC and CD45RO-ECD (Beckman Coulter, Miami, FL, USA

TCRβ chain CDR3 spectratyping

TCR Vβ spectratyping was performed by reverse transcriptase. Briefly, total mRNA was extracted from whole blood using Maxwell® RSC SimplyRNA Blood Kit according to manufacturer's recommendations (Promega, Madison WI). RNA was converted to cDNA using the SuperScript® VILO™. The TCRβ chain CDR3 diversity was determined by PCR with 25 forward TCR Vβ subfamily-specific primers and a FAM-labeled reverse Cβ primer as described previously[6] Denatured PCR products were electrophoresed and analyzed using 3500xl Genetic Analyzer (Applied Biosystems, Foster City, CA, USA). Spectratype complexity score was calculated as the summation of number of subfamilies with a maximum possible score of 200 (8 peaks for each family and 25 families, 8 X 25 = 200).

Quantification of single-joint TREC

Quantification of single-joint TREC was performed by real-time PCR using. Briefly, DNA from whole blood was extracted using the Maxwell® Blood DNA Kit according to the manufacturer's recommendations (Promega, Madison WI). Real-time PCR assay was performed on a QuantStudio™ 6 Flex (Applied Biosystems). Single-joint TREC plasmidic DNA used to make standard curve and primers were previously described[7]. Results were reported as Log_{10} TREC copies per milliliter blood. Values were compared to normal values[8].

Elispot assays

IFN-γ enzyme-linked immunospot (Elispot) assays, were conducted as previously described[9]. Briefly, PBMCs were plated in triplicate at a seeding density of $3x10^5$ cells/well. Cells were directly stimulated with peptide libraries (pepmixes; JPT Peptide Technology, Acton, MA) dissolved in

dimethyl sulfoxide (DMSO). These pepmixes comprised 15 amino acid long peptides with an 11 amino acid overlap, covering the coding regions of various proteins associated with distinct viruses. The proteins investigated included CMV pp65 (#PM-PP65-2) and IE-1 (#PM-IE-1); AdV serotype 5 penton protein (#PM-PM-HAdV5) and AdV serotype 3 hexon protein (#PM-HAdV3). Media with DMSO only served as a negative control, while Staphylococcus aureus Enterotoxin Type B (SEB) served as a positive control. Following 18–20 hours of incubation, plates underwent development, were dried at room temperature, and sent to Zellnet Consulting (New York, NY) for quantification. The frequency of T cells specific to each virus was normalized as spot-forming cells per 5×10^5 PBMCs.

Detailed statistical methodology

This was a phase II clinical trial with the primary objective to estimate the rate of successful ANC engraftment at 42 days post-HCT. The study was designed with Simon's 2-stage optimum design[10](one-sided type-1 error rate 10% and 80% power to test a null hypothesis of 80% 42-day engraftment rate against an alternative of 91%, resulting in planned sample size of 49 patients) and an expansion cohort. The study included stopping rules for excessive rates of aGVHD and therapy-related mortality, and the study was stopped due to triggering the aGVHD stopping rule after 72 of the planned 75 patients were enrolled. This article summarizes data as of April 2022.

This article analyzes the patients who received study HCT, excluding study patients treated with maraviroc. We summarize analyses as the 'full cohort' and the subset of these patients in complete remission at the time of HCT as the 'CR cohort'.

Because the rate of aGVHD grade III/IV was trending higher than the goal rate, and the aGVHD was noted to be predominantly GVHD of the gut, the drug maraviroc was added to the treatment regimen via Amendment 3.0 with the goal of reducing GI GVHD. However, it became apparent that patients receiving maraviroc had a higher instead of lower rate of aGVHD and the study was paused and amended to remove maraviroc and return to the original regimen. Because the rate of aGVHD was substantially and statistically different in patients receiving maraviroc versus those who did not, the IRB agreed that patients receiving maraviroc would not be considered evaluable for protocol endpoints and would be reported separately (**Supplementary Table 1**).

Per protocol, we report the primary endpoint with the proportion of patients with successful ANC engraftment at day +42 and its 95% Blyth-Still-Casella confidence interval.

All time-to-event endpoints were defined with HCT date as day 0. We defined overall survival (OS) as the time from HCT to death by any cause and event-free survival (EFS) as the time from HCT to death by any cause or relapse. We estimated OS and EFS probabilities with Kaplan-Meier (KM) methods. We separately estimated the cumulative incidence of relapse (CIR), grade III/IV aGVHD and moderate-severe cGVHD using the cumulative incidence function approach with the competing risk of death by any cause. We analyzed non-relapse mortality (NRM) similarly, using death without prior relapse as the event and relapse as the competing risk. Patients were

censored as of their last follow-up date. For the OS analysis, patients who received a second HCT were censored at the date of their second HCT, and with a sensitivity analysis, censoring for second HCT does not impact our conclusions (other analyses are unaffected by censoring due to second HCT because all patients with second HCT relapsed prior to second HCT and none experienced the outcomes of grade III/IV aGVHD or moderate-severe cGVHD). Additionally, we analyzed OS after incidence of grade III/IV aGVHD or any cGVHD by re-defining the day 0 of OS as date of grade III/IV aGVHD or any cGVHD, respectively. We report the KM estimates of 1-year post-aGVHD OS probability and both 1- and 3-year post-cGVHD OS probabilities.

One patient with active disease at the time of HCT is excluded from the CIR analyses, as they died without a post-transplant disease evaluation.

We conducted univariable analyses to explore the association between baseline/treatment characteristics and time-to-event endpoints with the logrank test for the OS and EFS endpoints and Gray's test for all others. To assess the sensitivity of these results to possible confounding, we specified confounder-adjusted multivariable Cox (EFS, OS) or Fine-Gray (all others) models and report the adjusted results (details in next subsection).

We report the univariable associations between occurrence of aGVHD or cGVHD and time-to-event endpoints by including them as time-varying covariates in Cox models (for the outcomes with competing risks, we use these cause-specific hazards models instead of Fine-Gray models)[11]. Firth penalties[12] were used in both full and CR cohort relapse models with cGVHD as a covariate and in the CR cohort NRM model with cGVHD as a covariate due to the fitting algorithm failing to converge in the respective unpenalized model.

Inferential results are intended to be exploratory, and there is no adjustment for the study's stopping rules or multiple testing. We report 95% two-sided confidence intervals (complementary log-log for univariable KM and cumulative incidence estimates, details for multivariable results below) and two-sided p-values. Baseline and treatment characteristics, along with exploratory endpoints of immune reconstitution and viral reactivation, are summarized descriptively. Analyses were performed using SAS 9.4 and R 4.1. The R survival, cmprsk and coxphf packages were used.

Statistical details of multivariable models
We fit multivariable models to assess the sensitivity of univariable analyses to possible confounding. **Table S1** summarizes the model specifications based on clinical judgment after univariable analyses. However, due to the limited sample size and numbers of events, the models could not all be fit as specified. The fitted models and results are in the main text **Table 3**.

Fitting relapse as an outcome in the CR cohort did not converge with the Fine-Gray model. Instead, we report this sensitivity analysis with a Cox cause-specific model with Firth penalty. We acknowledge this as a limitation, as changes to the model type will affect interpretations. For this

model, the reported confidence intervals and p-values are based on the profile penalized log likelihood. Otherwise, Wald-based confidence intervals and p-values are reported.

In these models, we included CD3 and CD45 tertiles as numeric variables, so e.g., the coefficient of CD3 is interpreted as a confounder-adjusted (log) hazard ratio comparing groups differing in one tertile of CD3. The covariates of donor gender and donor relationship were combined into a single binary covariate of “mother as donor” due to clinical interest and for parsimony.

As GVHD variables were not covariates of interest, incidences of aGVHD and cGVHD were coded as baseline covariates for these analyses to focus on sensitivity of univariable analyses specific to confounder adjustment and minimize other changes to the multivariable models.

Table S1. Covariates specified for confounder-adjusted multivariable models by full and CR cohorts*

Endpoint	Model	Number of events in full cohort	Full cohort	Number of events in CR cohort	CR cohort
OS	Cox PH	22	Disease phase, CD3, donor gender, aGVHD, cGVHD CMV	10	Disease phase, CD3, aGVHD, cGVHD CMV
NRM	Fine-Gray	10	CD3, donor gender, donor rel aGVHD, cGVHD	5	Donor gender, cGVHD aGVHD
EFS	Cox PH	30	Disease phase, CMV aGVHD cGVHD NK allo	15	CMV, HLA, aGVHD cGVHD Disease phase
Relapse	Fine-Gray	20	Disease phase, CMV, cGVHD aGVHD NK allo	10	Disease phase, CMV, HLA MM aGVHD cGVHD NK allo
aGVHD	Fine-Gray	21	NK Alloreactivity, KIR MM CD3 dose CD45RA dose	16	NK Alloreactivity, KIR MM CD3 dose CD45RA dose
cGVHD	Fine-Gray			10	Donor gender/rel CD3 dose CD45RA dose

*Underlined variables are covariates of interest, other variables are possible confounders.

References

1. Ruggeri, L., Aversa, F., Martelli, M.F. & Velardi, A. Allogeneic hematopoietic transplantation and natural killer cell recognition of missing self. *Immunol Rev* **214**, 202-218 (2006).
2. Gwozdowicz, S.*, et al.* KIR specificity and avidity of standard and unusual C1, C2, Bw4, Bw6 and A3/11 amino acid motifs at entire HLA:KIR interface between NK and target cells, the functional and evolutionary classification of HLA class I molecules. *Int J Immunogenet* **46**, 217-231 (2019).
3. Ruggeri, L.*, et al.* Effectiveness of donor natural killer cell alloreactivity in mismatched hematopoietic transplants. *Science* **295**, 2097-2100 (2002).
4. Leung, W. Infusions of allogeneic natural killer cells as cancer therapy. *Clin Cancer Res* **20**, 3390-3400 (2014).
5. Iyengar, R.*, et al.* Purification of human natural killer cells using a clinical-scale immunomagnetic method. *Cytotherapy* **5**, 479-484 (2003).
6. Maslanka, K., Piatek, T., Gorski, J., Yassai, M. & Gorski, J. Molecular analysis of T cell repertoires. Spectratypes generated by multiplex polymerase chain reaction and evaluated by radioactivity or fluorescence. *Hum Immunol* **44**, 28-34 (1995).
7. Hazenberg, M.D.*, et al.* Increased cell division but not thymic dysfunction rapidly affects the T-cell receptor excision circle content of the naive T cell population in HIV-1 infection. *Nat Med* **6**, 1036-1042 (2000).
8. Lorenzi, A.R.*, et al.* Determination of thymic function directly from peripheral blood: a validated modification to an established method. *J Immunol Methods* **339**, 185-194 (2008).
9. Kim-Hoehamer, Y.I.*, et al.* Development of a cGMP-compliant process to manufacture donor-derived, CD45RA-depleted memory CD19-CAR T cells. *Gene Ther* (2022).
10. Simon, R. Optimal two-stage designs for phase II clinical trials. *Control Clin Trials* **10**, 1-10 (1989).
11. Poguntke, I., Schumacher, M., Beyersmann, J. & Wolkewitz, M. Simulation shows undesirable results for competing risks analysis with time-dependent covariates for clinical outcomes. *BMC Med Res Methodol* **18**, 79 (2018).
12. Heinze, G. & Schemper, M. A solution to the problem of monotone likelihood in Cox regression. *Biometrics* **57**, 114-119 (2001).

Supplementary Table 1. Characteristics and outcome of patients who received Maraviroc

Pt #	Age at HCT	Diagnosis	Disease status	Day of neutrophil engraftment	Day of platelet engraftment	Day of Maraviroc final dose	Day of aGVHD Onset	aGVHD Max Grade	aGVHD Sites	cGVHD Max Grade	cGVHD Sites	Outcome
M1	1.5	Biphenotypic leukemia	CR1	10	12	24	25	4	Skin, Gut, Liver	Severe	Skin, Eye Oral	Alive, disease free cGVHD ongoing
M2	1.6	AML	CR1	12	13	16						Rejection, Re-grafted Alive, disease free
M3	1	Biphenotypic leukemia	CR2	9	55	27	27	3	Gut			Diffuse Alveolar Hemorrhage Expired D+108
M4	2.9	AML	CR1	10	15	30	39	3	Liver			Progressive disease Expired Month 17
M5	11.1	AML	PIF	11	16	30	33	4	Gut	Severe	Skin, Liver	Alive, disease free cGVHD resolved
M6	9.8	ALL	CR1	9	15	22	27	3	Skin, Gut, Liver	Mild	Skin, Liver, Eye, Oral	Alive, disease free cGVHD resolved

Abbreviations : AML, Acute myeloid leukemia; ALL, Acute Lymphoblastic leukemia; CR, Complete Remission; aGVHD; acute graft versus host disease; cGVHD, chronic graft versus host disease.

Supplementary Table 2A: Median cell counts before and after CD34 enrichment, CD45RA+ cell depletion and NK-cell enrichment

	Preprocessing	Postprocessing	Recovery %	Log depletion
**CD34 enriched product**				
TNC ($\times10^8$/kg)	12.3	0.099		
CD34+ ($\times10^6$/kg)	14.4	9.55	68.7	
CD3+ ($\times10^6$/kg)	401	0.005		4.81
**CD45RA-depleted product**				
TNC ($\times10^8$/kg)	11.9	4.65		
CD34+ ($\times10^6$/kg)	10.4	5.95	55.9	
CD3+ ($\times10^6$/kg)	303.3	58.95	22.5	0.7
CD45RA+ (×106/kg)	555	0.6		3.1
CD3+ CD45RA+ ($\times10^6$/kg)	105.6	<0.001		>5.02
CD19+ ($\times10^6$/kg)	68.4	0.2		2.4
CD3–CD56+ ($\times10^6$/kg)		0.4		
**NK-cell enriched product**				
TNC (×108/kg)	3.4	0.108		
CD3–CD56+ (×106/kg)	23.5	10.56	42.3	
CD3+ (×106/kg)	147	0.008		4.28
CD3+CD56–(×106/kg)	134.9	<0.001		>5.13
CD19+ (×106/kg)	32.6	0.031		2.99
Abbreviation: TNC= total nucleated cell. (n= 72) median values.				

Supplementary Table 2B: Total infused cell doses and individual graft compositions (CD34+, CD45RA-, NK)

	**Median (Range) n=72**
**CD34 enriched product**	
CD34+ ($\times10^6$/kg)	9.765 (1.96-44.64)
CD3+ ($\times10^6$/kg)	0.006 (0.001-0.088)
**CD45RA-depleted product**	
CD34+ ($\times10^6$/kg)	5.82 (0.578-39.43)
CD3+ ($\times10^6$/kg)	60.08 (16.08-528.4)
CD45RA+ ($\times10^6$/kg)	0.586 (0.009-4.382)
CD3+CD45RA+ ($\times10^6$/kg)	0 (0-0.2)
**NK-cell enriched product**	
CD56+ ($\times10^6$/kg)	10.88 (1.26-99.19)
CD3+ ($\times10^6$/kg)	0.008 (0.001-0.123)
CD3+CD56–($\times10^6$/kg)	0 (0-0.014)
**Total Infused cell doses**	
CD34+ ($\times10^6$/kg)	16.43 (2.948-67.55)
CD3+ ($\times10^6$/kg)	60.09 (16.08-528.6)
CD56+ ($\times10^6$/kg)	10.88 (1.26-99.19)

Supplemental Table 3: Common and Notable Adverse Events

Adverse Events	Patient Number
Acute Infusion Reaction	2
Acute Kidney Injury/renal failure	11
Bronchiolitis obliterans organizing pneumonia	1
CRS/Engraftment syndrome/Macrophage Activation Syndrome	33
Encephalopathy	2*
Fever	72
Graft Rejection	1
Hemorrhage, Gastrointestinal	5
Hemorrhage, Pulmonary	4
Hemorrhagic Cystitis	10
Hypertension	17
Mucositis	48
Seizure	6
Veno-occlusive Disease	4
**Infections**	
**Viral infection#**	
Adenovirus viremia	6
BK viremia	4
Cytomegalovirus viremia	25
Epstein Barr Virus viremia	9
Human Herpes Virus-6 viremia	28
**Bacterial infection**	
Bacteremia	34
Clostridium difficile infection	30
**Fungal/other infection**	
Thrush	25
Other	9

*both patients with encephalopathy had HHV-6 viremia, but HHV-6 was not detected by PCR in CSF

#PCR testing for Adenovirus, cytomegalovirus, Epstein Barr Virus was performed weekly in the first 100 days regardless of symptoms, BK virus and Human Herpes Virus-6 testing was only as clinically indicated (cystitis and fever/rash, respectively)

Supplementary Table 4a. Acute GVHD (aGvHD) Treatment (n=26)

aGVHD Treatment	# Patient
Methylprednisolone/Prednisone	23
Infliximab	10
Ruxolitinib	1
Tacrolimus	10
Sirolimus	7
Mycophenolate mofetil	4
Dexamethasone (Decadron)	2
Desonide/Betamethasone (topical)	7
Budesonide/Beclomethosone	10

Supplementary Table 4b. Acute GVHD (aGVHD) Medications and Outcomes

Pat #	Sites	Grade	aGVHD medications	Duration of systemic steroids (days)	aGVHD outcome
1	Liver, skin	4	Methylprednisolone, Tacrolimus, Infliximab, Sirolimus	3	aGVHD liver improving when relapsed
9	Gut	3	Methylprednisolone	28	resolved
10	Gut, skin	3	Methylprednisolone, Infliximab, Sirolimus, Mycophenolate mofetil, Prednisone, Dexamethasone	65	resolved
11	Liver, skin	3	Methylprednisolone, Sirolimus, Mycophenolate mofetil, Dexamethasone, Desonide	31	resolved
13	Gut, skin	3	Sirolimus, Mycophenolate mofetil, Desonide, Budesonide		resolved
20	Gut, skin	2	Mycophenolate mofetil, Prednisone Desonide	61	resolved
27	Skin	2	Methylprednisolone, Prednisone	11	resolved
33*	Gut	4	Methylprednisolone, Tacrolimus, Infliximab, Prednisone	149	resolved
38*	Skin	1	Tacrolimus, Prednisone	35	resolved
41	Liver	4	Methylprednisolone, Infliximab, Sirolimus Prednisone	58	resolved
42	Gut	3	Methylprednisolone, Tacrolimus, Infliximab, Sirolimus, Budesonide, Beclomethasone	53	resolved
43	Gut	3	Methylprednisolone, Prednisone, Budesonide, Beclomethasone	80	not resolved at death
45	Gut, skin	4	Methylprednisolone, Tacrolimus, Infliximab, Prednisone, Budesonide	80	resolved
49	Skin	2	Methylprednisolone, Prednisone, Desonide	36	resolved
50	Gut, skin	3	Methylprednisolone, Tacrolimus, Sirolimus, Prednisone, Desonide, Budesonide	8	aGVHD skin ongoing when relapsed
51	Gut	3	Budesonide, Beclomethasone		resolved
53	Gut	3	Methylprednisolone, Tacrolimus, Prednisone, Beclomethasone	80	resolved
54	Gut, skin	3	Methylprednisolone, Prednisone, Desonide, Budesonide, Beclomethasone, Betamethasone	26	resolved
57	Skin	1	Desonide, Betamethasone		resolved
65	Gut	3	Methylprednisolone, Tacrolimus, Infliximab, Budesonide, Beclomethasone, Ruxolitinib	73	resolved
67	Gut	3	Methylprednisolone, Prednisone	50	resolved
68	Gut, skin	4	Methylprednisolone, Infliximab, Prednisone	26	resolved
69	Gut	3	Methylprednisolone, Infliximab, Prednisone, Budesonide, Beclomethasone	29	resolved
70	Gut	3	No treatment		resolved
71	Gut, skin	3	Tacrolimus, Methylprednisolone	28	resolved
72	Gut	3	Tacrolimus, Methylprednisolone, Prednisone	59	resolved

*patients were weaned to alternate day low dose steroid and only duration of daily steroids was measured

Supplementary Table 5: Cause of death for NRM

Pt #	Diagnosis at Enrollment	Pre-HCT Disease Status	Detectable disease at HCT	Day of death	Cause of death
11	Infant AML	CR1	None	1478	Severe cGVHD of the skin and joints. Multi-drug resistant pseudomonas superinfection of the skin; progressive organ dysfunction.
2	Pre-B ALL	Relapse1	73% blasts	13	Acute onset cardiac failure, arrythmia, cardiac arrest
9	AML	PIF	71% blasts	74	Idiopathic pneumonia syndrome, coincided with CMV viremia clearance; bilateral pneumothorax, bronchopleural fistulae, respiratory failure.
17	Pre-B ALL	CR3	None	26	Acute encephalopathy, viral PCRs negative in CSF, VOD, multiorgan failure
3	Therapy-related AML	CR1, MRD+	0.01% MRD	498	Strenotrophomonas maltophlia bacteremia, multiorgan failure. History of cGVHD, off therapy at time of death
7	Infant ALL	PIF	63% blasts	469	Sudden infant death syndrome. Streptococcus pneumoniae bacteremia found on autopsy
22	AML	Relapse1	10%	41	Acute hepatic failure, HHV6 viremia, progressive encephalopathy
43	MDS-AML	CR1	Not detectable	331	cGVHD of lung, human metapneumovirus infection, respiratory failure
37	MDS-AML	PIF	9% blasts	1029	cGVHD of the lungs, multiple infections, respiratory failure
20	Biphenotypic Leukemia	CR1	0.003% MRD	1926	cGVHD of lung, respiratory failure

Abbreviations: AML, Acute myeloid leukemia; ALL, Acute lymphoblastic leukemia; MDS, myelodysplastic syndrome; CR, Complete Remission; aGVHD; acute graft versus host disease; cGVHD, chronic graft versus host disease; MRD, Minimal Residual Disease; PIF, Primary Induction Failure; CSF, Cerebrospinal Fluid; VOD, Veno-occlusive disease; HHV6 – human herpes virus 6

Supplementary Table 6: Complete univariable analysis results*

Characteristic	Full Cohort (n=72)					CR Cohort (n=53)				
Overall Survival (OS)										
	N	Events	1-year OS(95% CI)	3-year OS (95% CI)	P	N	Events	1-year OS(95% CI)	3-year OS (95% CI)	P
Patient gender					0.7659					0.5131
Male	42	13	80.7(65.0,89.8)	69.8(52.9,81.7)		32	5	93.8(77.3,98.4)	87.1(69.0,94.9)	
Female	30	9	86.7(68.3,94.8)	76.4(56.7,88.0)		21	5	90.5(67.0,97.5)	85.7(62.0,95.2)	
Patient age					0.2571					0.4766
<3	13	3	91.7(53.9,98.8)	82.5(46.1,95.3)		9	1	100	100	
3 to <10	26	11	76.7(55.3,88.8)	55.9(34.5,72.8)		19	5	83.9(57.9,94.5)	72.7(46.3,87.6)	
>=10	33	8	84.8(67.4,93.4)	81.7(63.7,91.3)		25	4	96.0(74.8,99.4)	92.0(71.6,97.9)	
Donor Gender					0.0953					0.5149
Male	38	8	92.0(77.1,97.3)	83.1(66.0,92.0)		33	5	97.0(80.4,99.6)	90.5(73.4,96.8)	
Female	34	14	73.5(55.3,85.3)	61.5(43.0,75.5)		20	5	85.0(60.4,94.9)	80.0(55.1,92.0)	
Donor age					0.2609					0.3101
<40	47	17	83.0(68.8,91.1)	69.2(53.5,80.5)		33	8	90.9(74.4,97.0)	84.8(67.4,93.4)	
>=40	25	5	83.8(62.4,93.6)	79.4(57.3,90.9)		20	2	95.0(69.5,99.3)	89.4(63.8,97.3)	
Leukemia type					0.5336					0.726
ALL	29	8	86.1(67.0,94.5)	75.3(55.1,87.4)		25	5	92.0(71.6,97.9)	83.6(62.0,93.5)	
AML	38	13	78.9(62.1,88.8)	66.6(48.4,79.6)		25	4	91.8(71.1,97.9)	87.7(66.4,95.8)	
Disease phase					**<.0001**					0.5634
CR1	25	4	96.0(74.8,99.4)	92.0(71.6,97.9)		25	4	96.0(74.8,99.4)	92.0(71.6,97.9)	
CR2 or more	28	6	89.1(70.0,96.4)	81.4(60.9,91.8)		28	6	89.1(70.0,96.4)	81.4(60.9,91.8)	
Active disease	19	12	57.9(33.2,76.3)	31.6(11.4,54.3)						
MRD at HCT					0.4157					0.4157
Negative	38	8	89.3(74.0,95.9)	81.2(64.6,90.6)		38	8	89.3(74.0,95.9)	81.2(64.6,90.6)	
Positive	9	1	100	100		9	1	100	100	
CMV Serostatus					0.1995					0.5062
D-/R-	10	2	90.0(47.3,98.5)	90.0(47.3,98.5)		10	2	90.0(47.3,98.5)	90.0(47.3,98.5)	
D-/R+	12	6	57.1(25.4,79.6)	57.1(25.4,79.6)		6	1	100	100	
D+/R-	10	2	90.0(47.3,98.5)	77.1(34.5,93.9)		7	0	100	100	

D+/R+	40	12	87.4(72.4,94.6)	71.6(54.6,83.2)		30	7	89.9(71.8,96.6)	79.5(59.9,90.2)	
CD34					0.434					0.9479
2.95-11.4	24	6	83.3(61.5,93.4)	79.2(57.0,90.8)		18	3	94.4(66.6,99.2)	88.9(62.4,97.1)	
11.5-21.0	24	10	79.2(57.0,90.8)	61.4(38.8,77.8)		14	3	92.9(59.1,99.0)	85.7(53.9,96.2)	
21.5-67.6	24	6	86.9(64.6,95.6)	77.2(53.5,89.9)		21	4	89.9(65.3,97.4)	84.7(59.5,94.8)	
CD45RA					0.5192					0.4183
0.009-0.337	24	9	66.7(44.3,81.7)	62.5(40.3,78.4)		14	2	92.9(59.1,99.0)	85.7(53.9,96.2)	
0.343-0.859	24	5	91.7(70.6,97.8)	82.7(60.2,93.2)		20	3	90.0(65.6,97.4)	90.0(65.6,97.4)	
0.884-4.382	24	8	91.5(70.0,97.8)	72.2(47.9,86.6)		19	5	94.4(66.6,99.2)	83.3(56.8,94.3)	
CD3					**0.0462**					0.2943
16.08-45.0	24	4	91.7(70.6,97.8)	83.3(61.5,93.4)		20	2	100	90.0(65.6,97.4)	
45.34-91.1	24	6	79.2(57.0,90.8)	74.5(51.7,87.7)		17	3	88.2(60.6,96.9)	82.4(54.7,93.9)	
91.98-528.5	24	12	78.3(55.4,90.4)	59.1(35.8,76.4)		16	5	86.2(55.0,96.4)	86.2(55.0,96.4)	
NK alloreactivity					0.4241					0.181
Yes	62	20	80.4(68.1,88.4)	71.8(58.5,81.4)		45	10	90.9(77.6,96.5)	84.0(69.2,92.0)	
No	10	2	100	75.0(29.8,93.4)		8	0	100	100	
HLA MM					0.6752					0.9177
2	1	0	100	100		1	0	100	100	
3	17	4	94.1(65.0,99.1)	87.4(58.1,96.7)		13	3	100	92.3(56.6,98.9)	
4	54	18	79.5(66.0,88.1)	67.7(53.3,78.6)		39	7	89.6(74.6,96.0)	84.3(68.4,92.6)	
Donor relationship					0.1203					0.5846
Father	35	8	91.2(75.2,97.1)	81.5(63.2,91.2)		30	5	96.7(78.6,99.5)	89.5(70.9,96.5)	
Mother	31	14	71.0(51.6,83.7)	57.9(38.7,73.0)		18	5	83.3(56.8,94.3)	77.8(51.1,91.0)	
Sibling	4	0	100	100		3	0	100	100	
Other	2	0	100	100		2	0	100	100	
KIR MM (rec-lig)					0.6335					0.3615
0	10	2	100	75.0(29.8,93.4)		8	0	100	100	
1	47	16	80.6(66.1,89.4)	71.7(56.3,82.5)		34	8	91.0(74.6,97.0)	84.9(67.5,93.4)	
2	15	4	80.0(50.0,93.1)	72.0(41.1,88.6)		11	2	90.9(50.8,98.7)	80.8(42.3,94.9)	
KIR MM (lig-lig)					0.2321					0.0962
0	37	8	89.1(73.5,95.8)	76.4(57.9,87.5)		26	2	96.2(75.7,99.4)	92.1(72.1,98.0)	

1	30	12	76.5(57.0,88.1)	66.1(46.1,80.1)		23	7	86.7(64.3,95.5)	77.6(54.3,90.0)	
2	5	2	80.0(20.4,96.9)	80.0(20.4,96.9)		4	1	100	100	
Donor KIR B score					0.2653					0.6675
0	23	9	82.6(60.1,93.1)	68.6(45.0,83.7)		17	5	94.1(65.0,99.1)	82.4(54.7,93.9)	
1	30	11	75.9(55.9,87.7)	64.6(44.1,79.2)		18	3	88.1(60.2,96.9)	88.1(60.2,96.9)	
2	14	2	92.9(59.1,99.0)	85.7(53.9,96.2)		14	2	92.9(59.1,99.0)	85.7(53.9,96.2)	
3	4	0	100	100		3	0	100	100	
Occurrence of aGVHD**					0.454					0.102
Occurrence of cGVHD**					0.141					0.077
**Event-Free Survival (EFS)**										
	**N**	**Events**	**1-year EFS (95% CI)**	**3-year EFS (95% CI)**	**P**	**N**	**Events**	**1-year EFS (95% CI)**	**3-year EFS (95% CI)**	**P**
Patient gender					0.566					0.8653
Male	42	18	64.3(47.9,76.7)	59.1(42.6,72.3)		32	9	78.1(59.5,88.9)	75.0(56.2,86.6)	
Female	30	12	73.3(53.7,85.7)	66.7(46.9,80.5)		21	6	85.7(62.0,95.2)	81.0(56.9,92.4)	
Patient age					0.399					0.5294
<3	13	6	69.2(37.3,87.2)	61.5(30.8,81.8)		9	2	88.9(43.3,98.4)	88.9(43.3,98.4)	
3 to <10	26	13	57.7(36.8,73.9)	49.7(29.5,67.0)		19	7	68.4(42.8,84.4)	63.2(37.9,80.4)	
>=10	33	11	75.8(57.3,87.1)	72.7(54.1,84.8)		25	6	88.0(67.3,96.0)	84.0(62.8,93.7)	
Donor Gender					0.2205					0.5291
Male	38	13	71.1(53.9,82.8)	68.4(51.1,80.7)		33	8	81.8(63.9,91.4)	78.8(60.6,89.3)	
Female	34	17	64.7(46.3,78.2)	55.6(37.4,70.4)		20	7	80.0(55.1,92.0)	75.0(50.0,88.7)	
Donor age					0.9926					0.3102
<40	47	20	66.0(50.6,77.6)	61.3(45.7,73.6)		33	8	81.8(63.9,91.4)	81.8(63.9,91.4)	
>=40	25	10	72.0(50.1,85.5)	64.0(42.2,79.4)		20	7	80.0(55.1,92.0)	70.0(45.1,85.3)	
Leukemia type					0.9949					0.4095
ALL	29	12	65.5(45.4,79.7)	62.1(42.1,76.9)		25	8	72.0(50.1,85.5)	72.0(50.1,85.5)	
AML	38	17	65.8(48.5,78.5)	57.3(40.0,71.4)		25	6	88.0(67.3,96.0)	80.0(58.4,91.1)	
Disease phase					**<.0001**					0.1904

CR1	25	5	92.0(71.6,97.9)	88.0(67.3,96.0)		25	5	92.0(71.6,97.9)	88.0(67.3,96.0)	
CR2 or more	28	10	71.4(50.9,84.6)	67.9(47.3,81.8)		28	10	71.4(50.9,84.6)	67.9(47.3,81.8)	
Active disease	19	15	31.6(12.9,52.2)	19.7(5.5,40.3)						
MRD at HCT					0.2992					0.2992
Negative	38	9	78.9(62.3,88.9)	76.3(59.4,86.9)		38	9	78.9(62.3,88.9)	76.3(59.4,86.9)	
Positive	9	4	88.9(43.3,98.4)	77.8(36.5,93.9)		9	4	88.9(43.3,98.4)	77.8(36.5,93.9)	
CMV Serostatus					**0.0046**					0.1699
D-/R-	10	2	90.0(47.3,98.5)	90.0(47.3,98.5)		10	2	90.0(47.3,98.5)	90.0(47.3,98.5)	
D-/R+	12	9	33.3(10.3,58.8)	33.3(10.3,58.8)		6	3	66.7(19.5,90.4)	66.7(19.5,90.4)	
D+/R-	10	3	80.0(40.9,94.6)	68.6(30.5,88.7)		7	0	100	100	
D+/R+	40	16	70.0(53.3,81.7)	62.5(45.7,75.4)		30	10	76.7(57.2,88.1)	70.0(50.3,83.1)	
CMV Recipient status					0.0514					**0.0454**
Positive	52	25	61.5(47.0,73.2)	55.8(41.3,68.0)		36	13	75.0(57.5,86.1)	69.4(51.7,81.8)	
Negative	20	5	85.0(60.4,94.9)	79.7(54.5,91.9)		17	2	94.1(65.0,99.1)	94.1(65.0,99.1)	
CD34					0.6398					0.5779
2.95-11.4	24	8	75.0(52.6,87.9)	70.8(48.4,84.9)		18	5	83.3(56.8,94.3)	77.8(51.1,91.0)	
11.5-21.0	24	12	58.3(36.4,75.0)	53.8(32.3,71.2)		14	3	85.7(53.9,96.2)	85.7(53.9,96.2)	
21.5-67.6	24	10	70.8(48.4,84.9)	62.5(40.3,78.4)		21	7	76.2(51.9,89.3)	71.4(47.2,86.0)	
CD45RA					0.9856					0.5595
0.009-0.337	24	10	62.5(40.3,78.4)	58.3(36.4,75.0)		14	3	85.7(53.9,96.2)	78.6(47.2,92.5)	
0.343-0.859	24	10	75.0(52.6,87.9)	66.7(44.3,81.7)		20	6	85.0(60.4,94.9)	80.0(55.1,92.0)	
0.884-4.382	24	10	66.7(44.3,81.7)	61.5(38.9,77.9)		19	6	73.7(47.9,88.1)	73.7(47.9,88.1)	
CD3					0.0861					0.2032
16.08-45.0	24	6	79.2(57.0,90.8)	79.2(57.0,90.8)		20	4	85.0(60.4,94.9)	85.0(60.4,94.9)	
45.34-91.1	24	9	66.7(44.3,81.7)	62.5(40.3,78.4)		17	3	88.2(60.6,96.9)	82.4(54.7,93.9)	
91.98-528.5	24	15	58.3(36.4,75.0)	45.5(25.1,63.7)		16	8	68.8(40.5,85.6)	62.5(34.9,81.1)	
NK alloreactivity					0.1685					0.0958
Yes	62	28	64.5(51.3,75.0)	59.7(46.4,70.7)		45	15	77.8(62.6,87.4)	73.3(57.8,83.9)	
No	10	2	90.0(47.3,98.5)	75.0(29.8,93.4)		8	0	100	100	
HLA MM					0.3639					0.0594

2	1	1	0	0		1	1	0	0	
3	17	8	64.7(37.7,82.3)	58.8(32.5,77.8)		13	5	76.9(44.2,91.9)	69.2(37.3,87.2)	
4	54	21	70.4(56.3,80.7)	64.4(50.0,75.7)		39	9	84.6(68.9,92.8)	82.1(66.0,91.0)	
Donor relationship					0.1291					0.4043
Father	35	13	68.6(50.5,81.2)	65.7(47.6,78.9)		30	8	80.0(60.8,90.5)	76.7(57.2,88.1)	
Mother	31	17	61.3(42.0,75.8)	51.4(32.8,67.2)		18	7	77.8(51.1,91.0)	72.2(45.6,87.4)	
Sibling	4	0	100	100		3	0	100	100	
Other	2	0	100	100		2	0	100	100	
KIR MM (rec-lig)					0.3835					0.2468
0	10	2	90.0(47.3,98.5)	75.0(29.8,93.4)		8	0	100	100	
1	47	21	66.0(50.6,77.6)	61.7(46.3,73.9)		34	11	79.4(61.6,89.6)	76.5(58.4,87.5)	
2	15	7	60.0(31.8,79.7)	53.3(26.3,74.4)		11	4	72.7(37.1,90.3)	63.6(29.7,84.5)	
KIR MM (lig-lig)					0.6058					0.3617
0	37	14	73.0(55.6,84.4)	64.2(46.3,77.5)		26	5	88.5(68.4,96.1)	84.6(64.0,93.9)	
1	30	13	63.3(43.6,77.8)	60.0(40.5,75.0)		23	8	73.9(50.9,87.3)	69.6(46.6,84.2)	
2	5	3	60.0(12.6,88.2)	60.0(12.6,88.2)		4	2	75.0(12.8,96.1)	75.0(12.8,96.1)	
Donor KIR B score					0.519					0.6997
0	23	10	73.9(50.9,87.3)	64.2(40.8,80.4)		17	6	82.4(54.7,93.9)	76.5(48.8,90.4)	
1	30	15	53.3(34.3,69.1)	50.0(31.3,66.1)		18	5	72.2(45.6,87.4)	72.2(45.6,87.4)	
2	14	4	85.7(53.9,96.2)	78.6(47.2,92.5)		14	4	85.7(53.9,96.2)	78.6(47.2,92.5)	
3	4	1	75.0(12.8,96.1)	75.0(12.8,96.1)		3	0	100	100	
Occurrence of aGVHD**					0.659					0.660
Occurrence of cGVHD**					0.764					0.461
**Cumulative Incidence of Non-Relapse Mortality (NRM)**										
	**N**	**Events**	**1-year NRM (95% CI)**	**3-year NRM (95% CI)**	**P**	**N**	**Events**	**1-year NRM (95% CI)**	**3-year NRM (95% CI)**	**P**
Patient gender					0.7597					0.3793
Male	42	6	9.5(3.0,20.7)	14.7(5.8,27.5)		32	2	3.1(0.2,14.0)	6.3(1.1,18.4)	
Female	30	4	3.3(0.2,14.9)	6.7(1.1,19.5)		21	3	4.8(0.3,20.3)	4.8(0.3,20.3)	

Patient age					0.9185					0.7617
<3	13	2	0	7.7(0.4,30.8)		9	1	0	0	
3 to <10	26	4	11.5(2.8,27.2)	15.7(4.7,32.5)		19	1	5.3(0.3,22.2)	5.3(0.3,22.2)	
>=10	33	4	6.1(1.0,17.9)	9.1(2.3,21.9)		25	3	4.0(0.3,17.4)	8.0(1.3,22.9)	
Donor Gender					**0.0064**					0.0622
Male	38	1	0	0		33	1	0	0	
Female	34	9	14.7(5.3,28.7)	23.9(11.0,39.4)		20	4	10.0(1.6,27.8)	15.0(3.5,34.1)	
Donor age					0.6259					0.7807
<40	47	6	4.3(0.8,13.0)	8.9(2.8,19.7)		33	3	3.0(0.2,13.6)	3.0(0.2,13.6)	
>=40	25	4	12.0(2.9,28.0)	16.0(4.8,33.0)		20	2	5.0(0.3,21.1)	10.0(1.6,27.8)	
Leukemia type					0.5839					0.3196
ALL	29	3	6.9(1.2,20.0)	10.3(2.5,24.6)		25	1	4.0(0.3,17.4)	4.0(0.3,17.4)	
AML	38	6	7.9(2.0,19.3)	13.7(4.9,27.1)		25	3	4.0(0.3,17.4)	8.0(1.3,22.9)	
Disease phase					0.1198					0.1026
CR1	25	4	4.0(0.3,17.4)	8.0(1.3,22.9)		25	4	4.0(0.3,17.4)	8.0(1.3,22.9)	
CR2 or more	28	1	3.6(0.2,15.7)	3.6(0.2,15.7)		28	1	3.6(0.2,15.7)	3.6(0.2,15.7)	
Active disease	19	5	15.8(3.7,35.6)	27.6(9.0,50.4)						
MRD at HCT					0.8925					0.8925
Negative	38	3	5.3(0.9,15.7)	7.9(2.0,19.3)		38	3	5.3(0.9,15.7)	7.9(2.0,19.3)	
Positive	9	1	0	0		9	1	0	0	
CMV Serostatus					0.9386					0.5948
D-/R-	10	2	10.0(0.5,37.4)	10.0(0.5,37.4)		10	2	10.0(0.5,37.4)	10.0(0.5,37.4)	
D-/R+	12	2	8.3(0.4,32.3)	8.3(0.4,32.3)		6	1	0	0	
D+/R-	10	1	0	11.4(0.4,42.0)		7	0	0	0	
D+/R+	40	5	7.5(1.9,18.4)	12.5(4.5,24.8)		30	2	3.3(0.2,14.8)	6.7(1.1,19.5)	
CD34					0.5957					0.7206
2.95-11.4	24	2	4.2(0.3,18.0)	8.3(1.4,23.8)		18	2	5.6(0.3,23.1)	11.1(1.7,30.4)	
11.5-21.0	24	5	12.5(3.0,29.1)	17.0(5.1,34.8)		14	1	0	0	
21.5-67.6	24	3	4.2(0.3,18.1)	8.3(1.3,23.8)		21	2	4.8(0.3,20.3)	4.8(0.3,20.3)	
CD45RA					0.5826					0.6373
0.009-0.337	24	5	16.7(5.0,34.1)	20.8(7.3,39.0)		14	2	7.1(0.4,28.5)	14.3(2.1,37.6)	

0.343-0.859	24	2	0	4.2(0.3,18.1)		20	1	0	0	
0.884-4.382	24	3	4.2(0.3,18.2)	9.3(1.4,26.4)		19	2	5.3(0.3,22.2)	5.3(0.3,22.2)	
CD3					**0.0326**					0.2173
16.08-45.0	24	0	0	0		20	0	0	0	
45.34-91.1	24	3	8.3(1.4,23.7)	12.5(3.0,29.1)		17	2	5.9(0.3,24.2)	11.8(1.8,31.9)	
91.98-528.5	24	7	12.5(3.0,29.1)	21.2(7.4,39.8)		16	3	6.3(0.3,25.8)	6.3(0.3,25.8)	
NK alloreactivity					0.7863					0.3839
Yes	62	9	8.1(2.9,16.6)	11.3(4.9,20.6)		45	5	4.4(0.8,13.5)	6.7(1.7,16.5)	
No	10	1	0	15.0(0.5,51.1)		8	0	0	0	
HLA MM					0.5301					0.9755
2	1	0	0	0		1	0	0	0	
3	17	1	0	0		13	1	0	0	
4	54	9	9.3(3.4,18.8)	15.2(7.0,26.3)		39	4	5.1(0.9,15.4)	7.7(1.9,18.9)	
Donor relationship					**0.0273**					0.1831
Father	35	1	0	0		30	1	0	0	
Mother	31	9	16.1(5.7,31.2)	26.0(12.0,42.5)		18	4	11.1(1.7,30.4)	16.7(3.9,37.3)	
Sibling	4	0	0	0		3	0	0	0	
Other	2	0	0	0		2	0	0	0	
KIR MM (rec-lig)					0.9054					0.5718
0	10	1	0	15.0(0.5,51.1)		8	0	0	0	
1	47	7	8.5(2.7,18.7)	10.6(3.8,21.4)		34	3	2.9(0.2,13.3)	2.9(0.2,13.3)	
2	15	2	6.7(0.4,26.9)	13.3(1.9,35.7)		11	2	9.1(0.4,34.7)	18.2(2.4,45.7)	
KIR MM (lig-lig)					0.3148					0.0769
0	37	3	2.7(0.2,12.3)	8.8(2.1,21.4)		26	0	0	0	
1	30	6	13.3(4.1,28.1)	16.7(5.9,32.1)		23	4	8.7(1.4,24.7)	13.0(3.1,30.2)	
2	5	1	0	0		4	1	0	0	
Donor KIR B score					0.1429					0.424
0	23	6	8.7(1.4,24.6)	18.4(5.4,37.4)		17	3	0	5.9(0.3,24.4)	
1	30	4	10.0(2.5,23.9)	13.3(4.1,28.2)		18	2	11.1(1.7,30.5)	11.1(1.7,30.5)	
2	14	0	0	0		14	0	0	0	
3	4	0	0	0		3	0	0	0	

Occurrence of aGVHD**					0.475					0.202
Occurrence of cGVHD**					**0.033**					**0.015**
**Cumulative Incidence of Relapse (CIR)*** **										
	**N**	**Events**	**1-year CIR (95% CI)**	**3-year CIR (95% CI)**	**P**	**N**	**Events**	**1-year CIR (95% CI)**	**3-year CIR (95% CI)**	**P**
Patient gender					0.7846					0.4659
Male	41	12	26.8(14.3,41.0)	26.8(14.3,41.0)		32	7	18.8(7.5,34.0)	18.8(7.5,34.0)	
Female	30	8	23.3(10.1,39.7)	26.7(12.4,43.3)		21	3	9.5(1.5,26.6)	14.3(3.4,32.6)	
Patient age					0.4391					0.2044
<3	13	4	30.8(8.8,56.5)	30.8(8.8,56.5)		9	1	11.1(0.5,40.6)	11.1(0.5,40.6)	
3 to <10	25	9	32.0(14.9,50.6)	36.0(17.8,54.7)		19	6	26.3(9.2,47.4)	31.6(12.4,52.9)	
>=10	33	7	18.2(7.2,33.1)	18.2(7.2,33.1)		25	3	8.0(1.3,22.9)	8.0(1.3,22.9)	
Donor Gender					0.5043					0.5064
Male	38	12	28.9(15.5,43.9)	31.6(17.5,46.6)		33	7	18.2(7.2,33.1)	21.2(9.2,36.5)	
Female	33	8	21.2(9.2,36.6)	21.2(9.2,36.6)		20	3	10.0(1.6,27.8)	10.0(1.6,27.8)	
Donor age					0.6606					0.3792
<40	47	14	29.8(17.4,43.2)	29.8(17.4,43.2)		33	5	15.2(5.4,29.5)	15.2(5.4,29.5)	
>=40	24	6	16.7(5.0,34.2)	20.8(7.3,39.0)		20	5	15.0(3.5,34.1)	20.0(6.0,39.9)	
Leukemia type					0.6893					0.1366
ALL	28	9	28.6(13.3,46.0)	28.6(13.3,46.0)		25	7	24.0(9.5,42.1)	24.0(9.5,42.1)	
AML	38	11	26.3(13.5,41.1)	28.9(15.5,43.9)		25	3	8.0(1.3,22.9)	12.0(2.9,28.1)	
Disease phase					**0.0012**					**0.0114**
CR1	25	1	4.0(0.3,17.4)	4.0(0.3,17.4)		25	1	4.0(0.3,17.4)	4.0(0.3,17.4)	
CR2 or more	28	9	25.0(10.8,42.2)	28.6(13.3,46.0)		28	9	25.0(10.8,42.2)	28.6(13.3,46.0)	
Active disease	18	10	55.6(29.1,75.6)	55.6(29.1,75.6)						
MRD at HCT					0.2636					0.2636
Negative	38	6	15.8(6.3,29.2)	15.8(6.3,29.2)		38	6	15.8(6.3,29.2)	15.8(6.3,29.2)	
Positive	9	3	11.1(0.5,40.6)	22.2(2.8,53.1)		9	3	11.1(0.5,40.6)	22.2(2.8,53.1)	
CMV Serostatus					**0.011**					0.1222
D-/R-	10	0	0	0		10	0	0	0	

D-/R+	12	7	58.3(24.4,81.4)	58.3(24.4,81.4)		6	2	33.3(3.2,70.4)	33.3(3.2,70.4)	
D+/R-	10	2	20.0(2.6,49.0)	20.0(2.6,49.0)		7	0	0	0	
D+/R+	39	11	23.1(11.3,37.3)	25.6(13.2,40.1)		30	8	20.0(7.9,36.0)	23.3(10.1,39.7)	
CMV Recipient status					**0.0357**					**0.0172**
Positive	51	18	31.4(19.2,44.3)	33.3(20.8,46.4)		36	10	22.2(10.3,37.0)	25.0(12.3,40.0)	
Negative	20	2	10.0(1.6,27.8)	10.0(1.6,27.8)		17	0	0	0	
CD34					0.9371					0.7037
2.95-11.4	24	6	20.8(7.3,39.0)	20.8(7.3,39.0)		18	3	11.1(1.7,30.4)	11.1(1.7,30.4)	
11.5-21.0	23	7	30.4(13.1,49.8)	30.4(13.1,49.8)		14	2	14.3(2.1,37.5)	14.3(2.1,37.5)	
21.5-67.6	24	7	25.0(9.9,43.6)	29.2(12.6,48.0)		21	5	19.0(5.7,38.3)	23.8(8.3,43.6)	
CD45RA					0.7209					0.354
0.009-0.337	23	5	21.7(7.6,40.5)	21.7(7.6,40.5)		14	1	7.1(0.4,28.6)	7.1(0.4,28.6)	
0.343-0.859	24	8	25.0(9.9,43.6)	29.2(12.6,48.0)		20	5	15.0(3.6,34.0)	20.0(6.0,39.9)	
0.884-4.382	24	7	29.2(12.6,48.0)	29.2(12.6,48.0)		19	4	21.1(6.3,41.6)	21.1(6.3,41.6)	
CD3					0.8027					0.2581
16.08-45.0	24	6	20.8(7.4,39.0)	20.8(7.4,39.0)		20	4	15.0(3.6,34.0)	15.0(3.6,34.0)	
45.34-91.1	24	6	25.0(9.9,43.6)	25.0(9.9,43.6)		17	1	5.9(0.3,24.3)	5.9(0.3,24.3)	
91.98-528.5	23	8	30.4(13.1,49.8)	34.8(16.2,54.2)		16	5	25.0(7.3,48.0)	31.3(10.8,54.5)	
NK alloreactivity					0.1736					0.1633
Yes	61	19	27.9(17.2,39.5)	29.5(18.6,41.3)		45	10	17.8(8.2,30.3)	20.0(9.8,32.8)	
No	10	1	10.0(0.5,37.4)	10.0(0.5,37.4)		8	0	0	0	
HLA MM					0.09					**0.0236**
2	1	1	100	100		1	1	100	100	
3	17	7	35.3(13.8,57.8)	41.2(17.8,63.4)		13	4	23.1(5.1,48.5)	30.8(8.8,56.5)	
4	53	12	20.8(11.0,32.6)	20.8(11.0,32.6)		39	5	10.3(3.2,22.2)	10.3(3.2,22.2)	
Donor relationship					0.3936					0.5909
Father	35	12	31.4(16.9,47.1)	34.3(19.1,50.1)		30	7	20.0(8.0,35.9)	23.3(10.1,39.7)	
Mother	30	8	23.3(10.1,39.7)	23.3(10.1,39.7)		18	3	11.1(1.7,30.4)	11.1(1.7,30.4)	
Sibling	4	0	0	0		3	0	0	0	
Other	2	0	0	0		2	0	0	0	

KIR MM (rec-lig)					0.3806					0.347
0	10	1	10.0(0.5,37.4)	10.0(0.5,37.4)		8	0	0	0	
1	46	14	26.1(14.4,39.4)	28.3(16.1,41.8)		34	8	17.6(7.0,32.2)	20.6(8.9,35.6)	
2	15	5	33.3(11.4,57.4)	33.3(11.4,57.4)		11	2	18.2(2.4,45.7)	18.2(2.4,45.7)	
KIR MM (lig-lig)					0.7419					0.9672
0	37	11	24.3(11.9,39.1)	27.0(13.9,42.0)		26	5	11.5(2.8,27.1)	15.4(4.7,31.8)	
1	29	7	24.1(10.4,40.9)	24.1(10.4,40.9)		23	4	17.4(5.2,35.4)	17.4(5.2,35.4)	
2	5	2	40.0(3.1,78.6)	40.0(3.1,78.6)		4	1	25.0(0.3,71.4)	25.0(0.3,71.4)	
Donor KIR B score					0.6185					0.687
0	22	4	18.2(5.5,36.8)	18.2(5.5,36.8)		17	3	17.6(4.1,39.0)	17.6(4.1,39.0)	
1	30	11	36.7(19.8,53.7)	36.7(19.8,53.7)		18	3	16.7(3.9,37.2)	16.7(3.9,37.2)	
2	14	4	14.3(2.1,37.5)	21.4(4.8,45.8)		14	4	14.3(2.1,37.5)	21.4(4.8,45.8)	
3	4	1	25.0(0.3,71.4)	25.0(0.3,71.4)		3	0	0	0	
Occurrence of aGVHD**					0.322					0.705
Occurrence of cGVHD**					0.080					0.217
**Cumulative Incidence of Grade III-IV aGVHD**										
**Characteristic**	**N**	**Events**	**1-year G 3/4 aGVHD (95% CI)**	**3-year G 3/4 aGVHD (95% CI)**	**P-value**	**N**	**Events**	**1-year G 3/4 aGVHD (95% CI)**	**3-year G 3/4 aGVHD (95% CI)**	**P-value**
Patient gender					0.8543					0.2696
Male	42	12	28.6(15.8,42.7)	28.6(15.8,42.7)		32	8	25.0(11.6,41.0)	25.0(11.6,41.0)	
Female	30	9	30.0(14.8,46.9)	30.0(14.8,46.9)		21	8	38.1(17.8,58.3)	38.1(17.8,58.3)	
Patient age					0.4278					0.6867
<3	13	3	23.1(5.1,48.5)	23.1(5.1,48.5)		9	3	33.3(6.7,64.0)	33.3(6.7,64.0)	
3 to <10	26	10	38.5(20.0,56.7)	38.5(20.0,56.7)		19	7	36.8(15.9,58.1)	36.8(15.9,58.1)	
>=10	33	8	24.2(11.2,39.9)	24.2(11.2,39.9)		25	6	24.0(9.5,42.1)	24.0(9.5,42.1)	
Donor Gender					0.9601					0.9918
Male	38	11	28.9(15.5,43.9)	28.9(15.5,43.9)		33	10	30.3(15.6,46.4)	30.3(15.6,46.4)	
Female	34	10	29.4(15.1,45.2)	29.4(15.1,45.2)		20	6	30.0(11.8,50.7)	30.0(11.8,50.7)	
Donor age					0.6357					0.6582

<40	47	15	31.9(19.1,45.5)	31.9(19.1,45.5)		33	11	33.3(17.9,49.6)	33.3(17.9,49.6)	
>=40	25	6	24.0(9.5,42.1)	24.0(9.5,42.1)		20	5	25.0(8.8,45.4)	25.0(8.8,45.4)	
Leukemia type					0.2328					0.3799
ALL	29	6	20.7(8.2,37.0)	20.7(8.2,37.0)		25	6	24.0(9.5,42.1)	24.0(9.5,42.1)	
AML	38	13	34.2(19.6,49.4)	34.2(19.6,49.4)		25	9	36.0(17.8,54.6)	36.0(17.8,54.6)	
Disease phase					0.3769					0.1733
CR1	25	10	40.0(20.8,58.6)	40.0(20.8,58.6)		25	10	40.0(20.8,58.6)	40.0(20.8,58.6)	
CR2 or more	28	6	21.4(8.5,38.2)	21.4(8.5,38.2)		28	6	21.4(8.5,38.2)	21.4(8.5,38.2)	
Active disease	19	5	26.3(9.2,47.4)	26.3(9.2,47.4)						
MRD at HCT					0.6946					0.6946
Negative	38	12	31.6(17.5,46.6)	31.6(17.5,46.6)		38	12	31.6(17.5,46.6)	31.6(17.5,46.6)	
Positive	9	2	22.2(2.8,53.1)	22.2(2.8,53.1)		9	2	22.2(2.8,53.1)	22.2(2.8,53.1)	
CMV Serostatus					0.9706					0.521
D-/R-	10	3	30.0(6.2,59.3)	30.0(6.2,59.3)		10	3	30.0(6.2,59.3)	30.0(6.2,59.3)	
D-/R+	12	4	33.3(9.4,60.0)	33.3(9.4,60.0)		6	3	50.0(7.7,82.9)	50.0(7.7,82.9)	
D+/R-	10	3	30.0(6.2,59.3)	30.0(6.2,59.3)		7	3	42.9(7.6,75.7)	42.9(7.6,75.7)	
D+/R+	40	11	27.5(14.7,41.9)	27.5(14.7,41.9)		30	7	23.3(10.1,39.7)	23.3(10.1,39.7)	
CD34					0.8425					0.5726
2.95-11.4	24	6	25.0(9.9,43.6)	25.0(9.9,43.6)		18	4	22.2(6.6,43.6)	22.2(6.6,43.6)	
11.5-21.0	24	7	29.2(12.6,48.1)	29.2(12.6,48.1)		14	4	28.6(8.2,53.3)	28.6(8.2,53.3)	
21.5-67.6	24	8	33.3(15.5,52.3)	33.3(15.5,52.3)		21	8	38.1(17.8,58.3)	38.1(17.8,58.3)	
CD45RA					0.1027					0.1032
0.009-0.337	24	5	20.8(7.3,39.0)	20.8(7.3,39.0)		14	2	14.3(2.1,37.6)	14.3(2.1,37.6)	
0.343-0.859	24	5	20.8(7.4,39.0)	20.8(7.4,39.0)		20	5	25.0(8.8,45.4)	25.0(8.8,45.4)	
0.884-4.382	24	11	45.8(25.0,64.5)	45.8(25.0,64.5)		19	9	47.4(23.6,67.9)	47.4(23.6,67.9)	
CD3					0.4781					0.686
16.08-45.0	24	7	29.2(12.6,48.0)	29.2(12.6,48.0)		20	6	30.0(11.8,50.7)	30.0(11.8,50.7)	
45.34-91.1	24	5	20.8(7.3,39.0)	20.8(7.3,39.0)		17	4	23.5(6.9,45.7)	23.5(6.9,45.7)	
91.98-528.5	24	9	37.5(18.5,56.5)	37.5(18.5,56.5)		16	6	37.5(14.7,60.6)	37.5(14.7,60.6)	
NK alloreactivity					**0.004**					**0.0066**
Yes	62	14	22.6(13.1,33.7)	22.6(13.1,33.7)		45	10	22.2(11.4,35.3)	22.2(11.4,35.3)	

No	10	7	70.0(28.2,90.4)	70.0(28.2,90.4)		8	6	75.0(23.2,94.5)	75.0(23.2,94.5)	
HLA MM					0.6029					0.5702
2	1	0	0	0		1	0	0	0	
3	17	6	35.3(13.8,57.8)	35.3(13.8,57.8)		13	5	38.5(13.1,63.9)	38.5(13.1,63.9)	
4	54	15	27.8(16.5,40.2)	27.8(16.5,40.2)		39	11	28.2(15.1,42.9)	28.2(15.1,42.9)	
Donor relationship					0.7909					0.9399
Father	35	10	28.6(14.7,44.1)	28.6(14.7,44.1)		30	9	30.0(14.8,46.9)	30.0(14.8,46.9)	
Mother	31	8	25.8(12.0,42.1)	25.8(12.0,42.1)		18	5	27.8(9.7,49.6)	27.8(9.7,49.6)	
Sibling	4	2	50.0(2.3,88.1)	50.0(2.3,88.1)		3	1	33.3(0.1,83.2)	33.3(0.1,83.2)	
Other	2	1	50.0(0.0,96.0)	50.0(0.0,96.0)		2	1	50.0(0.0,96.0)	50.0(0.0,96.0)	
KIR MM (rec-lig)					**0.0051**					**0.0142**
0	10	7	70.0(28.2,90.4)	70.0(28.2,90.4)		8	6	75.0(23.2,94.5)	75.0(23.2,94.5)	
1	47	13	27.7(15.7,41.0)	27.7(15.7,41.0)		34	9	26.5(13.0,42.1)	26.5(13.0,42.1)	
2	15	1	6.7(0.4,26.9)	6.7(0.4,26.9)		11	1	9.1(0.4,34.7)	9.1(0.4,34.7)	
KIR MM (lig-lig)					0.7967					0.9878
0	37	10	27.0(13.9,42.0)	27.0(13.9,42.0)		26	8	30.8(14.3,49.0)	30.8(14.3,49.0)	
1	30	10	33.3(17.2,50.4)	33.3(17.2,50.4)		23	7	30.4(13.2,49.8)	30.4(13.2,49.8)	
2	5	1	20.0(0.4,62.1)	20.0(0.4,62.1)		4	1	25.0(0.3,71.4)	25.0(0.3,71.4)	
Donor KIR B score					**0.0257**					**0.006**
0	23	6	26.1(10.3,45.2)	26.1(10.3,45.2)		17	5	29.4(10.2,51.9)	29.4(10.2,51.9)	
1	30	5	16.7(5.9,32.1)	16.7(5.9,32.1)		18	1	5.6(0.3,23.1)	5.6(0.3,23.1)	
2	14	8	57.1(26.6,78.9)	57.1(26.6,78.9)		14	8	57.1(26.6,78.9)	57.1(26.6,78.9)	
3	4	2	50.0(2.3,88.1)	50.0(2.3,88.1)		3	2	66.7(0.2,97.3)	66.7(0.2,97.3)	
**Cumulative Incidence of Moderate-Severe cGVHD**										
	**N**	**Events**	**1-year MS cGVHD (95% CI)**	**3-year MS cGVHD (95% CI)**	**P**	**N**	**Events**	**1-year MS cGVHD (95% CI)**	**3-year MS cGVHD (95% CI)**	**P**
Patient gender					0.6527					0.9376
Male	42	7	14.7(5.9,27.4)	17.4(7.5,30.6)		32	6	19.4(7.7,35.1)	19.4(7.7,35.1)	
Female	30	4	13.3(4.1,28.1)	13.3(4.1,28.1)		21	4	19.0(5.7,38.3)	19.0(5.7,38.3)	
Patient age					0.9963					0.8759
<3	13	2	16.7(2.3,42.6)	16.7(2.3,42.6)		9	2	25.0(3.0,57.9)	25.0(3.0,57.9)	
3 to <10	26	4	11.5(2.8,27.2)	15.6(4.7,32.4)		19	3	15.8(3.7,35.6)	15.8(3.7,35.6)	

>=10	33	5	15.2(5.4,29.5)	15.2(5.4,29.5)		25	5	20.0(7.1,37.6)	20.0(7.1,37.6)	
Donor Gender					0.067					**0.0166**
Male	38	3	8.2(2.1,20.0)	8.2(2.1,20.0)		33	3	9.5(2.4,22.8)	9.5(2.4,22.8)	
Female	34	8	20.6(8.9,35.6)	23.5(10.9,38.9)		20	7	35.0(15.1,55.8)	35.0(15.1,55.8)	
Donor age					0.0704					0.065
<40	47	10	19.1(9.4,31.6)	21.4(10.9,34.2)		33	9	27.3(13.4,43.2)	27.3(13.4,43.2)	
>=40	25	1	4.0(0.3,17.4)	4.0(0.3,17.4)		20	1	5.0(0.3,21.1)	5.0(0.3,21.1)	
Leukemia type					0.568					0.5011
ALL	29	3	10.8(2.6,25.5)	10.8(2.6,25.5)		25	3	12.5(3.0,29.2)	12.5(3.0,29.2)	
AML	38	6	13.2(4.7,26.0)	16.1(6.4,29.8)		25	5	20.0(7.1,37.6)	20.0(7.1,37.6)	
Disease phase					0.095					0.1465
CR1	25	7	28.0(12.1,46.4)	28.0(12.1,46.4)		25	7	28.0(12.1,46.4)	28.0(12.1,46.4)	
CR2 or more	28	3	11.1(2.7,26.3)	11.1(2.7,26.3)		28	3	11.1(2.7,26.3)	11.1(2.7,26.3)	
Active disease	19	1	0	6.6(0.3,27.5)						
MRD at HCT					0.6824					0.6824
Negative	38	6	16.2(6.5,29.8)	16.2(6.5,29.8)		38	6	16.2(6.5,29.8)	16.2(6.5,29.8)	
Positive	9	2	22.2(2.8,53.1)	22.2(2.8,53.1)		9	2	22.2(2.8,53.1)	22.2(2.8,53.1)	
CMV Serostatus					0.7974					0.5537
D-/R-	10	2	20.0(2.6,49.0)	20.0(2.6,49.0)		10	2	20.0(2.6,49.0)	20.0(2.6,49.0)	
D-/R+	12	1	8.3(0.4,32.8)	8.3(0.4,32.8)		6	1	16.7(0.5,54.9)	16.7(0.5,54.9)	
D+/R-	10	1	0	11.3(0.5,41.2)		7	0	0	0	
D+/R+	40	7	17.9(7.8,31.5)	17.9(7.8,31.5)		30	7	24.1(10.4,40.9)	24.1(10.4,40.9)	
CD34					0.6868					0.564
2.95-11.4	24	3	12.5(3.0,29.1)	12.5(3.0,29.1)		18	3	16.7(3.9,37.2)	16.7(3.9,37.2)	
11.5-21.0	24	5	16.7(5.0,34.2)	21.2(7.4,39.6)		14	4	28.6(8.2,53.3)	28.6(8.2,53.3)	
21.5-67.6	24	3	13.3(3.2,30.8)	13.3(3.2,30.8)		21	3	15.4(3.6,34.8)	15.4(3.6,34.8)	
CD45RA					0.4943					0.9027
0.009-0.337	24	2	8.3(1.4,23.8)	8.3(1.4,23.8)		14	2	14.3(2.1,37.6)	14.3(2.1,37.6)	
0.343-0.859	24	4	16.9(5.1,34.6)	16.9(5.1,34.6)		20	4	20.4(6.0,40.6)	20.4(6.0,40.6)	
0.884-4.382	24	5	17.4(5.3,35.5)	22.0(7.7,40.9)		19	4	22.2(6.6,43.6)	22.2(6.6,43.6)	
CD3					0.4641					0.4178

16.08-45.0	24	2	8.3(1.4,23.7)	8.3(1.4,23.7)		20	2	10.0(1.6,27.8)	10.0(1.6,27.8)	
45.34-91.1	24	4	16.7(5.0,34.2)	16.7(5.0,34.2)		17	4	23.5(6.9,45.8)	23.5(6.9,45.8)	
91.98-528.5	24	5	17.8(5.3,36.1)	22.4(7.8,41.6)		16	4	27.3(7.8,51.7)	27.3(7.8,51.7)	
NK alloreactivity					0.2165					0.7108
Yes	62	8	13.2(6.1,23.0)	13.2(6.1,23.0)		45	8	18.2(8.4,31.0)	18.2(8.4,31.0)	
No	10	3	20.0(2.6,49.0)	30.0(6.2,59.3)		8	2	25.0(3.0,57.9)	25.0(3.0,57.9)	
HLA MM					0.4279					0.4229
2	1	0	0	0		1	0	0	0	
3	17	1	5.9(0.3,24.2)	5.9(0.3,24.2)		13	1	7.7(0.4,30.3)	7.7(0.4,30.3)	
4	54	10	17.0(8.3,28.3)	18.9(9.6,30.5)		39	9	23.7(11.6,38.2)	23.7(11.6,38.2)	
Donor relationship					0.1328					**0.0347**
Father	35	2	6.0(1.0,17.7)	6.0(1.0,17.7)		30	2	7.0(1.2,20.4)	7.0(1.2,20.4)	
Mother	31	8	22.6(9.7,38.7)	25.8(11.9,42.2)		18	7	38.9(16.8,60.7)	38.9(16.8,60.7)	
Sibling	4	1	25.0(0.3,71.4)	25.0(0.3,71.4)		3	1	33.3(0.1,83.2)	33.3(0.1,83.2)	
Other	2	0	0	0		2	0	0	0	
KIR MM (rec-lig)					0.2866					0.5345
0	10	3	20.0(2.6,49.0)	30.0(6.2,59.3)		8	2	25.0(3.0,57.9)	25.0(3.0,57.9)	
1	47	5	10.9(3.9,21.9)	10.9(3.9,21.9)		34	5	15.2(5.4,29.5)	15.2(5.4,29.5)	
2	15	3	20.0(4.5,43.4)	20.0(4.5,43.4)		11	3	27.3(5.7,55.4)	27.3(5.7,55.4)	
KIR MM (lig-lig)					0.1782					0.0979
0	37	3	5.4(0.9,16.1)	8.4(2.1,20.4)		26	2	7.7(1.3,22.1)	7.7(1.3,22.1)	
1	30	7	24.2(10.4,41.1)	24.2(10.4,41.1)		23	7	31.8(13.7,51.7)	31.8(13.7,51.7)	
2	5	1	20.0(0.4,62.1)	20.0(0.4,62.1)		4	1	25.0(0.3,71.4)	25.0(0.3,71.4)	
Donor KIR B score					0.7864					0.4677
0	23	4	13.0(3.1,30.3)	17.4(5.2,35.5)		17	3	17.6(4.1,39.1)	17.6(4.1,39.1)	
1	30	5	17.5(6.2,33.5)	17.5(6.2,33.5)		18	5	30.1(10.3,52.9)	30.1(10.3,52.9)	
2	14	1	7.1(0.4,28.5)	7.1(0.4,28.5)		14	1	7.1(0.4,28.5)	7.1(0.4,28.5)	
3	4	1	25.0(0.3,71.4)	25.0(0.3,71.4)		3	1	33.3(0.1,83.2)	33.3(0.1,83.2)	

*Two-sided p-values are reported from the logrank (OS, EFS) and Gray's (NRM, CIR, aGVHD, cGVHD) tests

**Occurrence of aGVHD and cGVHD p-values are from the respective two-sided Wald test on the time-varying covariates in Cox model

***Excludes 1 patient with active disease at the time of HCT, as they died prior to engraftment and not at risk of relapse

Abbreviations: CR, Complete Remission; aGVHD; acute graft versus host disease; cGVHD, chronic graft versus host disease; MRD, Minimal Residual Disease; CMV, Cytomegalovirus; R, Recipient; D; Donor, NK, Natural Killer; HLA, Human Leukocyte Antigen; MM, mismatch; KIR, Killer Immunoglobulin Receptor; rec-lig, receptor-ligand; lig-lig, ligand-ligand.

St. Jude Children's Research Hospital

HAPNK1

Initial version, dated: 10/18/2012 (IRB approved: 03/04/2013) Activated: April 10, 2013
Revision 0.1, dated 03/29/2013 (IRB Approved: 04/23/2013) Activated: 05/02/2013
Revision 0.2, dated 05/02/13 (IRB Approved: 05/08/2013) Activated: 05/31/2013
Amendment 1.0, dated 07/03/13 (IRB Approved: 09/19/13) Activated: 09/25/13
Revision 1.1, dated 10/31/13 (IRB Approved: 1/6/14) Activated: January 14, 2014
Revision 1.2, dated 09/23/14 (IRB Approved: 10/23/14) Activated: November 3, 2014
Amendment 2.0, dated 8/17/15 (IRB Approved: 12/7/15) Activated: December 18, 2015
Amendment 3.0, dated 03/16/16 (IRB Approved:07/18/16) Activated: Aug.23, 2016
Amendment 4.0, dated 01/10/17 (IRB approved: 03/07/17) Activated: April 7, 2017
Un-numbered Revision, dated: 11/17/2017 (IRB Approved: 11/17/2017)
Amendment 5.0, dated 03/13/18 (IRB Approved: 5/8/2018) Activated: May 17, 2018
Revision 5.1, dated 06/13/2018 (IRB Approved: 10/11/2018) Activated: 10/31/2018

HAPLOIDENTICAL DONOR HEMATOPOIETIC PROGENITOR CELL AND NATURAL KILLER CELL TRANSPLANTATION WITH A TLI BASED CONDITIONING REGIMEN IN PATIENTS WITH HEMATOLOGIC MALIGNANCIES

IDE #15422

Principal Investigator

Brandon M. Triplett, MD[1]

Co-Investigators

Renee Madden, MD
Julia Hurwitz, PhD[5]
Ewelina Mamcarz, MD[1]
Thomas Merchant, DO, PhD[2]
Amr Qudeimat, MD[1]
Aimee Talleur, MD[1]
Anusha Sunkara, MS[3]
Akshay Sharma, MD[1]
Guolian Kang, PhD [3]
Ashok Srinivasan, MD[1]
Paul Thomas, PhD[6]
William Janssen, PhD[1]
Shane Cross, PharmD[4]
Ben Youngblood, PhD[6]
Paulina Velasquez, MD[1]

Department of Bone Marrow Transplantation and Cellular Therapy[1]
Division of Radiation Oncology, Department of Radiological Sciences[2]
Department of Biostatistics[3]
Department of Pharmaceutical Sciences[4]
Department of Infectious Diseases[5]
Department of Immunology[6]

St. Jude Children's Research Hospital
262 Danny Thomas Place
Memphis, TN 38105
(901) 595-3300

This is a confidential research document. No information may be extracted without permission of the principal investigator.

TABLE OF CONTENTS

Study Summary

1.0 Objectives………………………………………..………………………………….. 1

1.1 Primary Objectives…………………………………………….………………… 1

1.2 Secondary Objectives…………………………………………….………………. 1

1.3 Exploratory Objectives…………………………………………….…………….. 1

2.0 Background and Rationale……………………..……………………………… 1

2.1 Overview……………………………………………………………………………. 1

2.2 Indications for HCT and need for alternative donors ………………………………….. 2

2.3 Haploidentical HCT………………………………………………………………….. 2

2.4 Rationale for immunomagnetic T cell depletion of hematopoietic progenitor cell graft. 4

2.5 Rationale for the reduced intensity regimen…………………………………………… 7

2.6 Rationale for the therapeutic NK cell graft……………………………………………. 8

2.7 Minimal residual disease………………………………………………………………. 9

2.8 Rationale for the present study………………………………………………………… 10

2.9 Experience with GVHD prior to amendment 2.0 ……………………………………… 10

3.0 Protocol Eligibility Criteria……………………………………………………….. 11

3.1 Inclusion criteria for transplant recipients …………………………………………….. 11

3.2 Inclusion criteria for haploidentical donors …………………………………………… 13

3.3 Gender and minorities………………………………………………………………… 13

4.0 Treatment Plan……………………………………………………………………….. 14

4.1 Preparative regimen…………………………………….…………………………….. 14

4.2 Donor selection………………………………………………………………………….. 16

4.3 Donor mobilization and graft collections ……………………………………………. 17

4.4 Graft preparations…………………………………………………...……………… 18

4.5 Additional progenitor cell graft administration …………………………...……….. 20

4.6 Donor lymphocyte infusions………………………………………………………. 20

4.7 Quality assurance of cellular products …………………………………………….. 22

5.0 Medication and Device Information……………………………….………… 23

5.1 Medications……………….…………………………………..………………..… 23

5.2 CliniMACS™ System………………………………..…………………….………… 30
6.0 Required Observations and Evaluations…………………………..…………… 31
6.1 Pre/peri/post-transplant and long-term evaluations ………………………………… 31
6.2 Long-term follow-up evaluations……………………..………………………….. 31
6.3 Evaluation for chimerism and engraftment ………………………………….. 31
6.4 Evaluation for immune reconstitution ………………………………….……… 32
6.5 General viral surveillance……………..………………………………………. 32
6.6 Minimal residual disease evaluations …………………………………………… 32
6.7 Research tests on haploidentical donor …………..……………………………… 33
7.0 Evaluation Criteria………………...………………………………………… 33
7.1 Adverse event monitoring……………………………………………………….. 33
7.2 GVHD diagnosis and grading……………………………………………… 33
7.3 Performance Status……………………………………………………………… 34
7.4 Hematologic recovery…………………………………………………………. 34
7.5 Graft failure…………………………………………………………………….. 35
7.6 Chimerism………………………………………………………………………… 35
8.0 Off-Study Criteria……………………………………………………………… 35
8.1 Recipient criteria……………………………………………………………….. 35
8.2 Additional notes………………………………………………………………….. 35
8.3 Donor criteria…………………………………………………………………….. 36
9.0 Reporting Criteria and Continuing Review…………………………………….. 36
9.1 Reporting Adverse Experiences and Deaths to St. Jude IRB ……………………… 36
9.2 Reporting to St. Jude institutional biosafety committee ……………………………… 38
9.3 Reporting to FDA……………………………………………………………… 38
9.4 Reporting to St. Jude office of regulatory affairs …………………………………….. 39
9.5 Continuing review reports……………………………………………………….. 39
9.6 Data submission to Miltenyi Biotec……………………………………………… 39
9.7 Reporting to the CIBMTR……………………………………………………………… 39
10.0 Statistical Considerations……………………………………….………………... 40
10.1 Statistical design and analysis for primary objective and stopping rules ……..……… 40

10.2 Statistical analysis for secondary objectives 45

10.3 Analysis for exploratory objectives.. 45

11.0 Data Acquisition and Quality Assurance Monitoring 46

11.1 Enrollment on study.. 46

11.2 Data submission 46

11.3 Quality assurance monitoring... 46

12.0 Obtaining Informed Consent... 47

13.0 References... 48

A Appendix A.. 55

B Appendix B.. 56

C Appendix C.. 64

D Appendix D.. 67

E Appendix E.. 68

F Appendix F.. 72

G Appendix G.. 73

STUDY SUMMARY

Protocol Title

Haploidentical Donor Hematopoietic Progenitor Cell and Natural Killer Cell Transplantation with a TLI based Conditioning Regimen in Patients with Hematologic Malignancies

Principal Investigator:

Brandon M. Triplett, MD

Brief Overview ***Study Population:***

Patients with high-risk hematologic malignancies who would likely benefit from allogeneic hematopoietic cell transplantation (HCT). Patients who do not have a suitable HLA matched related or unrelated donor will typically be offered enrollment on one of two alternative donor protocols that will be run in parallel. Those with a suitable HLA matched sibling or unrelated donor identified will be eligible for participation ONLY if the donor is not available in the necessary time.

In general, patients who have a suitable HLA matched haploidentical donor (matched $\geq$ 3 of 6), will be preferentially offered enrollment on HAPNK1. If the patient is medically eligible, but there is no suitable KIR mismatched haploidentical donor available, suitable cord blood units are available, and the patient is eligible for an institutional cord blood protocol, then the patient will be prioritized for enrollment on the current institutional cord blood transplant protocol.

Participant must be less than or equal to 21 years old. Additional eligibility criteria are specified to assure sufficient multi-organ system function.

Intervention, Brief Outline, and Objectives of Treatment Plan:

In this study, participants with high-risk hematologic malignancies undergoing hematopoietic cell transplantation (HCT), who do not have a suitable human leukocyte antigen (HLA) matched related/sibling donor (MSD) or matched unrelated donor (MUD) identified, will receive a haploidentical donor HCT with additional natural killer (NK) cells.

The assessments and follow-up evaluations noted in the protocol follow the St. Jude standard operating procedures (SOP) for all recipients of allogeneic HCT.

The primary objective of the study is to estimate the rate of successful engraftment at day +42 posttransplant in patients undergoing haploidentical HCT plus NK cell transplantation with a TLI based conditioning regimen. Secondary aims will assess overall survival, event-free survival, risk of relapse, graft versus host disease (GVHD), transplant related mortality (TRM), transplant related morbidity, and immune reconstitution.

IND/IDE:

CliniMACS CD56^{+} enrichment -- IDE 15422 IDE Cross-reference:

CliniMACS CD34+ enrichment

CliniMACS CD45RA depletion *Criteria for Evaluation – Safety and Efficacy*

Safety:

The primary measures of safety will be the rate of therapy related death and the rate of severe graft versus host disease.

Ongoing assessment of toxicity will be done using the NCI CTCAE version 3.0

Acute and chronic graft-vs.-host disease will be evaluated using established staging criteria and expert consensus guidelines.

Efficacy:

The primary measure of efficacy will be rate of successful engraftment.
Neutrophil and platelet engraftment will be determined using the parameters put forth by the Center for International Blood and Marrow Registry. Assessments will be made upon review of daily complete blood count and serial chimerism studies.
Bone marrow studies for disease status evaluation will be performed at approximately 21 days, 100 days, and 1 year post-transplant. Testing will include a research evaluation for minimal residual disease.

Immune reconstitution will be determined by standard blood testing of T- and B cell subsets.
Research tests required for participation will examine NK and T-cell function and reconstitution.

Statistical Considerations and Data Analysis

***Study Design*: Phase II**

***Randomization*: No**

***Sample Size:* Up to 75 evaluable patients**

Data Analyses:

Anticipated primary completion date: 07/01/2019

Anticipated study completion date: 07/01/2020

Time frame for primary outcome measure: 42 days post-transplant

Data Management including statistical evaluations

Protocol compliance, data collection including safety data, and reporting will be carried out by the Department of Bone Marrow Transplantation and Cellular Therapy Research Office.

Statistical considerations and ongoing analysis will be conducted by Dr. Guolian Kang and designated associates within the St. Jude Department of Biostatistics.

Human Subjects:

The risks to participants are primarily related to the cellular infusions and the conditioning regimen. The allogeneic stem cells may induce serious and possibly fatal disorders such as GVHD, veno-occlusive disorder and post-transplant lymphoproliferative disease. Because of the required conditioning, recipients are at high-risk for serious and possibly life-threatening infection, bleeding, and anemia. Adverse events will be treated, monitored, and reported appropriately.

Possible benefits of participation include obtaining and/or sustaining disease remission. In addition, there is the possibility of psychological benefit from knowing participation has helped researchers gain more understanding about the efficacy of haploidentical HCT.

Alternatives to participation are identified as chemotherapy and/or irradiation without transplant, research treatment if available, and/or supportive therapy.

The possible benefits, alternatives to participation, and side effects, including that there may be unknown side effects of treatment, are detailed in lay language within the respective informed consent document.

1.0 OBJECTIVES

1.1 Primary objective

1.1.1 To estimate the rate of successful engraftment at day +42 post-transplant in patients who receive haploidentical donor stem cell plus NK cell transplantation with TLI based conditioning regimen for high risk hematologic malignancy.

1.2 Secondary objectives

1.2.1 Estimate the incidence of malignant relapse, event-free survival, and overall survival at one-year post-transplantation.

1.2.2 Estimate incidence and severity of acute and chronic (GVHD).

1.2.3 Estimate the rate of transplant related mortality (TRM) in the first 100 days after transplantation.

1.3 Exploratory objectives

1.3.1 Assess the relationship between pre-transplant minimal residual disease (MRD) with transplant outcomes.

1.3.2 Record immune reconstitution parameters, including chimerism analysis, quantitative lymphocyte subsets, T cell receptor excision circle (TREC) analysis, V-beta spectratyping, and lymphocyte phenotype and function.

1.3.3 Describe the use of CD45RA-depleted DLI for recipients who have severe viral infections, disease recurrence or progression, or poor immune reconstitution. Assess and record efficacy of CD45RA-depleted DLI for these conditions, and all adverse events that are related to CD45RA-depleted DLI.

2.0 BACKGROUND AND RATIONALE

2.1 Overview

Allogeneic HCT is a potential curative therapy for various pediatric hematologic malignancies, however approximately 25-60% of eligible pediatric recipients will not have a human leukocyte antigen (HLA)-matched related/sibling donor (MSD) or a HLA matched unrelated donor (MUD).[1-4] The lack of an adequate MUD for a substantial number of patients, and the lengthy duration of the donor search process in those with high risk of relapse, have prompted the search for alternative donors, including haploidentical family donors and unrelated umbilical cord blood (UCB) grafts.[3,4]

Nearly all patients have a readily available mismatched family member (haploidentical) donor, and haploidentical HCT is an effective therapy for patients with hematologic malignancies.[5,6] In particular, when extensive T-cell depletion was utilized, the presence of KIR mismatch dramatically reduced the risk of relapse in patients who received haploidentical HCT for high-risk leukemia.[7-9] This institution has a track record of success using haploidentical donors for patients with hematologic malignancies,[10] and we hypothesize that in patients who require an alternative donor HCT, those with a KIR mismatched donor are likely to benefit most from haploidentical HCT. Therefore, those

with only KIR matched haploidentical donors, or those without a suitable haploidentical donor, will be prioritized to UCB HCT. However, with the adoptive transfer of large numbers of memory T-cells in this study, NK cell alloreactivity may no longer be required for adequate control of relapse. Therefore, patients without suitable HLA-matched or UCB options, and only KIR matched haploidentical donors, may enroll on this study. Here, we propose a single institution prospective phase II trial to estimate the efficacy of haploidentical HCT with additional NK cells in pediatric patients with hematologic malignancies.

This study will evaluate the safety and efficacy of haploidentical donor allogeneic HCT with additional NK cells for patients with high risk hematologic malignancies, through the estimation of donor engraftment, survival, relapse, acute and chronic GVHD, chimerism, toxicity, disease recurrence, and post-transplant cellular therapies based on chimerism results.

2.2 Indications for HCT and the need for alternative donors

Allogeneic HCT has become a widely accepted curative therapy for many childhood hematologic malignancies that cannot be cured with chemotherapy alone.[11-14] Studies at St. Jude and other institutions have demonstrated that HCT is potentially curative for patients with CML, AML, ALL and MDS.[15-17] Furthermore, patients with non-Hodgkin or Hodgkin lymphoma who recur after an autologous HCT may be successfully treated with allogeneic HCT.18,19

Importantly, the criteria for patients who are suitable for allogeneic HCT (i.e. would be expected to benefit from allogeneic transplantation) change as new outcome data are compiled and published. For example, less than a decade ago, there was a clear advantage for allogeneic HCT for Ph+ ALL in CR1.[13] However, the use of tyrosine kinase inhibitors combined with intensive chemotherapy may have abrogated this advantage.[20,21] It is imperative that the transplant physician be aware of the latest data and consensus recommendations[14,22] when assessing the suitability for allogeneic HCT.

Unfortunately, only approximately 30% of patients with hematologic malignancies that are suitable for allogeneic HCT will have a suitable MSD and approximately another 30% will have an MUD. [1-4] Thus, a significant number of patients will not have an appropriate MSD or MUD donor available in a timely manner, and will require an alternative hematopoietic progenitor cell (HPC) source from haploidentical or unrelated UCB donors. Although, alternative donors have been shown to be suitable to treat children with high-risk hematologic malignancies, they may be associated with increase incidences of graft failure, TRM, GVHD, and delayed immune reconstitution when compared to recipients of MSD or MUD.[23,24] Novel strategies are still needed to optimize outcomes in this group.

2.3 Haploidentical HCT

Haploidentical donors are viable alternative donors since family members are highly motivated, easily accessible, and readily available for most patients. Along with other institutions, St. Jude has shown haploidentical HCT to be an effective therapy for patients with hematologic malignancies.[5,6,25-27] Due to the high potential for GVHD with the degree

of HLA mismatch seen in haploidentical HCT, haploidentical grafts are often extensively T-cell depleted prior to infusion. Early results with T-cell depleted haploidentical HCT were disappointing due very high rates of graft failure and infections.[28] Significant progress was achieved with the use of mega-dose of T-cell depleted HPC after high-intensity conditioning.[26] Aversa and colleagues reported successful long-term engraftment with minimal GVHD in 43 patients with leukemia (including 15 children) using high doses of $CD34^+$ enriched haploidentical cells, purified by positive ($CD34^+$) and negative (CD3-lectin agglutination) selection.[29,30] Utilizing haploidentical grafts and megadose cell therapy ($\geq 1 \times 10^7$ $CD34^+$ cells/kg), Handgretinger and colleagues reported similar results in 23 pediatric patients. Prompt engraftment with rapid immune reconstitution was associated with a low incidence of GVHD, even without the use of pharmacologic GVHD prophylaxis.[31]

Building on the initial studies above, St. Jude has gained considerable expertise with haploidentical HCT in children.[8-10,32-36] Over the past decade (2001-2010), more than 220 mismatched related donor HCT have been performed at St. Jude. Initially, most haploidentical HCT performed in this institution were for patients with relapsed and refractory hematologic malignancies, however as it became a more successful therapeutic maneuver, it has become a primary transplant option for patients who lack a well matched donor.[10]

Recent studies suggest that alloreactive NK cells play a role in graft-vs.-leukemia (GVL) and influence outcomes of patients with hematologic malignancies after haploidentical HCT.[7,3742] In addition to the anti-leukemia effects, NK cells are capable of reducing the incidence of graft rejection and GVHD in animal models; the former is in part based on the mechanism of donor NK cells against patient's T cells, and the latter on donor NK cells against patient's antigen presenting cells.[7,40] Taken together, the presence of alloreactive donor NK cells may have multiple advantageous effects, including reduction in (1) relapse (2) graft rejection (3)
GVHD and (4) viral infections.[43]

The NK cell alloreactivity depends on the balance of signals mediated through activating and inhibitory KIRs on the NK cell. KIRs recognize their ligands, HLA Class I molecules on recipient antigen presenting cells. Some of the inhibitory KIRs recognize specific HLA class I ligands including the well-defined specificity of KIR2DL2/3 for the HLA-Cw group-1 epitope, the specificity of 2DL1 for the Cw group-2 epitope, and the specificity of 3DL1 for the HLA Bw4 epitope. Determination of both donor KIR and recipient HLA typing, allows categorization of KIR match versus mismatch. KIR genes segregate independently of HLA genes, such that matching for HLA does not confirm KIR matching.

In patients who received extensively T-cell depleted haploidentical HCT for high risk leukemia at St. Jude, the presence of KIR mismatch dramatically reduced the risk of relapse.[9] Thus, patients with high risk hematologic malignancies who lack an HLA-matched donor but have a KIR mismatched haploidentical donor available would be expected to benefit the most from the NK cell alloreactivity in haploidentical HCT. Therefore, KIR mismatched donors will be prioritized when available.

2.4 Rationale for immunomagnetic T cell depletion of hematopoietic progenitor cell graft

Improvements in engraftment were achieved when Handgretinger et al demonstrated that HLA barriers of engraftment in recipients of haploidentical grafts maybe overcome by the infusion of a large number of $CD34^+$ cells (>10^7 $CD34^+$ cells/kg).[26] Collection of megadose $CD34^+$ has become feasible with the implementation of treating donors with G-CSF before donation, which has been shown to increase the number $CD34^+$ cell collected.[44]

Donor T cells in the haploidentical graft play a major role in mediating GVHD - as few as 3 x 10^4 Tcells/kg can cause GVHD.[27] However, new methods of TCD have been developed to generate a graft with very low T cell content, such that the risk of GVHD is decreased. T cells can be removed from the HPC graft by direct removal of T cells (negative selection) or selection of $CD34^+$ progenitors (positive selection). The transplantation program at St. Jude has had success using either method of T-cell depletion in haploidentical donor transplantation. In this study, CD34+ selection by CliniMACS will be the initial method used for T-cell depletion of the HPC graft. This was used successfully in INFT2 in which the HPC graft was often collected early and frozen until day 0 followed by collection and fresh infusion of an NK cell graft the following week.

Although positive selection allows for extensive T-cell depletion (TCD), the graft is devoid of other important cell populations such as NK cells and myeloid cells such as dendritic cells and monocytes.[45,46] In addition, this extensive degree of TCD has significant negative effects on the time to donor immune competency. Donor T cells are critical for reconstituting the allogeneic host immune system, and lymphocyte recovery is an important determinate of outcome post-transplant.[47,48] Transplants that employ TCD result in elimination of most memory T cells, leading to protracted immune dysfunction- an effect that becomes more severe with higher intensity conditioning regimens.[32,49] This results in an increased rate of opportunistic infections. Indeed, viral infections are the most common cause of death of children receiving haploidentical transplants.[50] The majority of these infections occur within the first 6 months following transplantation, when T cell immunity is the lowest.[51] Reconstitution of immunity can be partially restored by therapeutic infusions of donor cytotoxic lymphocytes.[47,52-54] However, in addition to requiring significant resources and expertise, the cells must be engineered or selected for specific infections - making this approach impractical for broad application.[55]

CD45, also called the leukocyte common antigen, is expressed on nearly all hematopoietic cells in various isoforms related to stage of development and activation.[56] Among the various isoforms is CD45RA, which selectively identifies naïve T cells.[57] Naïve T cells have fully matured but do not proliferate until they encounter the specific ligand that their receptor recognizes.[58] If such a proliferative signal occurs after antigen recognition, then there is a marked, antigen-specific cell expansion and inflammatory response. While many of these cells will undergo apoptosis after the initial response, others are rescued from immune retraction and will persist as memory T cells, the majority of which then become CD45RA.[59] Having responded to antigen, memory T cells are no longer naïve and are thus capable of more rapidly responding to future infectious challenges.[60] Once generated, memory cells persist in the circulation as a diverse cell pool that is critical for long-term infection control.[61]

A more ideal cell product infusion would offer adoptive transfer of a diverse lymphocyte repertoire capable of effectively rejecting a variety of pathogens, as well as malignant cells. It is hypothesized that the elimination of only CD45RA+ naïve T cells from an infused cell product would not significantly negatively impact memory responses to infection. Among CD4+ T cells, the CD45RA- subset has equivalent "helper" functions as CD45RA+ cells in the generation of alloreactive cytotoxic T cells.[62] Further, it is the CD45RA- memory cells that are responsible for aiding B-cell differentiation and antibody production.

The various strategies thus far reported for selective T cell depletion largely spare CD45RA+ naïve cells, including those that target CD25. Only approximately 5% of naive CD4 cells are
CD25+.[55] There is potential advantage in targeting naïve T cells for lymphocyte depletion. Human studies have shown that sorted donor CD45RA+ naïve T cells are far more alloreactive than all memory subsets tested.[63] In animal models, infusions of fractionated memory T cells do not cause physical or histologic evidence of GVHD, independent of T_{reg} numbers. Naïve T cells, conversely, are potent inducers of GVHD, even despite CD25 depletion before infusion.[55] Moreover, in similar models, lymphocyte infusions specifically depleted of naïve T cells do not cause GVHD.[64] Allografts containing high numbers of CD45RA+ naïve T_{regs} may be associated with lower risk of acute GVHD in haploidentical transplantation using unmanipulated, fully T cell-replete products.[65] The proposed approach will deplete this population - potentially abrogating this effect.

After showing such promise in animal models, selective CD45RA depletion has begun to be studied in human marrow transplantation, including at this institution. CD45RA depletion by magnetic beads is feasible and effective for naïve T-cell depletion. Data provided by Miltenyi has shown this method to be an effective means of depletion, with greater than 3-fold reduction in number of CD45RA+ cells. Experiments were performed in the Human Applications Laboratory to qualify the CD45RA+ depletion procedures. Hematopoietic progenitor cell products were purchased that were obtained from G-CSF mobilized normal donors by apheresis. The HPC,A products were depleted of CD45RA+ cells following a procedure provided by Miltenyi Biotech. Briefly, the cells were incubated with the CD45RA Microbead reagent followed by washing to remove unbound beads. The labeled cells were then applied to the CliniMACS device (Depletion Tubing Set) and the CD45RA+ cells removed using the Depletion 3.1 program. Flow cytometric analysis before and after depletion was performed following procedures provided by Miltenyi Biotech. The results of the experiments are presented in the table below. An example flow cytometric analysis of CD3+ cells is also included.

	Experiment # 1		Experiment #2	
	Pre-depletion (x 10^6)	Post-depletion (x 10^6)	Pre-depletion (x 10^6)	Post-depletion (x 10^6)
Nucleated Cells	29748.0	10270.0	26166.0	10981.0
CD3+	12238.3	4555.8	6353.1	1985.4
CD3+CD45RA+	7877.3	4.1	3520.8	2.2
CD34+	636.6	311.2	693.5	395.3
CD3+ CD45RA+ Log Depletion	3.28		3.21	

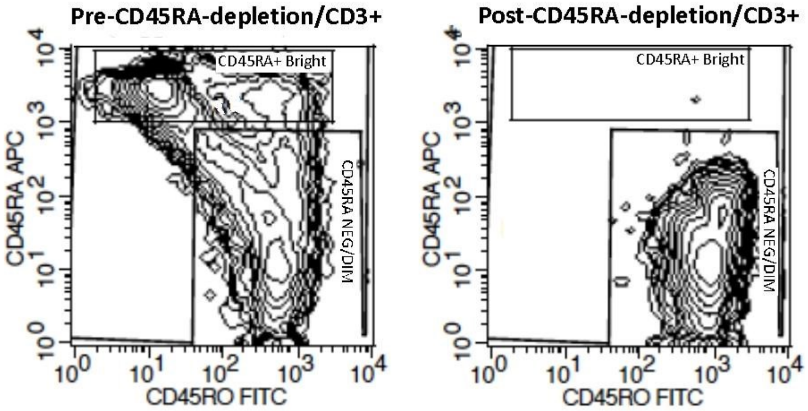

We hypothesize that the addition of CD45RA depleted product to a back bone of CD34 enriched cells will provide a HPC graft with good progenitor cell content (3-5 x 10^6 CD34+ cells/kg) and a useful memory T-cell fraction to the recipients on this trial.

For the 26 patients enrolled on this protocol prior to amendment 2.0, there was no maximum naïve T-cell (CD3+CD45RA+) dose. Although there is no correlation with naïve T-cell dose and the occurrence of GVHD so far in this or any other institutional CD45RA-depleted transplant protocol, rare patients have received naïve T-cell doses in excess of 10^5/kg. In HAPREF, which utilized CD3+ depletion, the conventional T-cell dose cap was raised to 1.5 x 10^5/kg, and the rate of acute GvHD was noted to be increased. Returning the maximum dose down to 1 x 10^5 CD3+ cells/kg in HIFLEX again provided an acceptably low rate of acute GVHD. Because approximately ½ of circulating T cells are naïve (and therefore 1 x 10^5 conventional T cells would contain approximately 0.5 x 10^5 naïve T-cells), in this trial the naïve T-cell (CD3+CD45RA+) dose in the CD45RA-depleted product will be capped at 0.5 x 10^5/kg (0.05 x 10^6) starting with amendment 2.0.

Of the first 26 patients to receive transplantation and complete one year of follow-up on this protocol, 4 have experienced morphologic relapse. Two of the 4 demonstrated haplotype loss.[66] This indicates that the adoptively transferred memory T-cells have anti-leukemia effects. In this setting then, KIR mismatch driven NK cell alloreactivity may no longer be required for disease control as it was in previous studies utilizing extensively T-cell depleted haploidentical donor transplantation.

2.5 Rationale for the reduced intensity conditioning regimen

The immunological effect of graft versus leukemia (GVL) after allogeneic HCT has allowed reduction in the intensity of conditioning as HCT was no longer reliant on the cytotoxicity of high dose of chemotherapy to eradicate disease. Reduced-intensity conditioning (RIC) regimens have the potential to improve the outcomes of allogeneic HCT by decreasing the acute TRM.[67-69] Fludarabine-based RIC regimens are safe and effective alternatives to total body irradiation (TBI) in haploidentical HCT.[32,70,71] In addition, intensification of immunoablative conditioning using ATG, OKT3, or alemtuzumab, results in the decrease incidence of GVHD and graft rejection as well.[71-74] In our first RIC haploidentical HCT protocol REFSCT, we evaluated a RIC regimen for pediatric patients with refractory hematological malignancies or considered at high risk for TRM with a full myeloablative regimen. The RIC regimen consisted of fludarabine (200mg/m^2), thiotepa (10mg/kg), melphalan (120 mg/m^2) and Orthoclone-OKT3 and enrolled 25 patients from 2003-2005. Out of the 25 participants, 22 engrafted with a median of 10 days (range, 7-12) and 3 experienced graft failure. The patients with graft failure, one patient had disease relapse and 2 were salvaged with stem cells infusions from their first transplant donor. The median time to platelet engraftment to 20,000/mm^3 and 50,000/mm^3 was 17 days (range, 12-36) and 17 days (range, 12-76), respectively. The cumulative incidence of overall grade III-IV acute GVHD and chronic GVHD was 8% and 28%, respectively. Of those participants who died, 13 were due to relapsed disease (at a median of 105 days post-HCT). Only 4 (16%) died of TRM, despite most of the patients having received one prior allogeneic HCT - 1 died of GVHD, 1 of infection, 1 of hemorrhage, and 1 of cardiomyopathy. In summary, RIC allows for engraftment with an acceptable rate for graft failure, GVHD, and TRM.

Two subsequent haploidentical HCT studies at St. Jude (HAPREF and HIFLEX) utilized non-TBI preparative regimens with a fludarabine, thiotepa, melphalan backbone. Twentyseven patients with high risk hematologic malignancies were transplanted on one of these two protocols with OKT3 as in vivo T-cell depletion, followed by infusion of a $CD3^+$ TCD graft. The one-year EFS was 51.9% ± 9.6%. These studies have demonstrated the EFS and TRM in TCD RIC HCT from mismatched haploidentical donor compare favorably with reports from MUD.

Importantly however, OKT3 was an important component of the preparative regimen for these transplants, and it is no longer commercially available. Our most recent front-line haploidentical donor HCT trial (HIFLEX) has substituted Campath for OKT3. Early results indicate that survival is similar between patients on HIFLEX treated with OKT3 versus Campath (data not shown). However, requirement for the use of therapeutic DLI has risen substantially. Only 1 of the 11 patients who received OKT3 on HIFLEX required more than 2 DLI in the first 100 days post-HCT. In contrast, 9 of the first 14 patients who received Campath on HIFLEX required more than 2 DLI in the first 100 days post-HCT. This is likely due to Campath's anti-NK cell effects, leading to an increased clinical need for early T-cell add back in the form of DLI. Unfortunately, with OKT3's withdrawal from market, there is no commercially available T-cell specific (NK cell sparing) antibody.

Therefore, in this protocol, we plan to use total lymphoid irradiation (TLI) in the preparative regimen to provide bidirectional tolerance in substitution of OKT3.

TLI was initially developed with curative intent in patients with Hodgkin Lymphoma, and it was its use in these patients that led to the discovery of TLI's alteration of T-cell specific immune responses. Years of pre-clinical research on TLI have shown that the use of TLI promotes engraftment and reduces GvHD.[75] This work was then followed up with therapeutic trials in humans with hematologic malignancy, which showed that conditioning with TLI and ATG alone allowed a high rate of durable donor engraftment, a low rate of GvHD, and preservation of evidence of graft versus malignancy effect, in patients who received HLAmatched related or unrelated donor grafts.[76,77] We have significant institutional experience using TLI-based regimens for allogeneic transplantation (n=29 as of June 2012). Importantly, TLI-based conditioning has allowed successful salvage haploidentical transplantation in patients who failed previous allo-HCT. Nine patients have received TLI-based conditioning with haploidentical donor HCT after experiencing previous allograft failure (3 had primary graft failure, 4 had initial engraftment with acute rejection, and 2 had late graft failure). The same haploidentical donor was utilized in 5 of the 9 salvage HCT, with 4 patients receiving a new (haploidentical) donor. Eight of 9 patients (89%) experienced durable engraftment at a median of 12 days (range 10 – 27 days). The remaining patient had primary graft failure due to progressive disease. This experience indicates that TLI is effective for facilitation of engraftment, even in patients receiving a mismatched haploidentical donor graft and with a history that indicates a very high risk of graft failure. There is additional published experience from Germany in which TLI – given as a single 7Gy fraction – was utilized in 14 adult and pediatric patients for reconditioning after graft failure/rejection.[78] Despite most of the patient having haploidentical donors, engraftment was obtained in all evaluable patients. In addition, TLI was well tolerated in this pediatric population that had recently received another (typically myeloablative) preparative regimen. Finally, 10 patients in Chile received TLI-containing preparative regimen, similar to our proposed regimen, as a part of their haploidentical donor preparative regimen.[79] TLI was given as a single dose at 7Gy. Nine out of 10 patients experienced rapid donor engraftment, with relapse as the cause of the one primary graft failure. Six of 10 patients were alive and disease free at one year, 3 died of progressive disease, and one of infection.

2.6 Rationale for the therapeutic NK cell graft

Relapse, infection, and GvHD remain major concerns after HCT. Donor lymphocyte infusion (DLI) containing all types of lymphocytes (B, T, and NK cells) is a viable option for the prevention and/or treatment of both relapse and infection, but conventional (unfractionated) DLI is limited by the risk of T-cell mediated GVHD.[80] Another therapeutic option is the infusion of large number of donor-derived NK cells.[43,81-83] In recent years, it has become clear that NK-cell alloreactivity can exert significant effects in allogeneic transplantation. NK-cell alloreactive effects can include a reduction in the rate of rejection, a reduction in GvHD, and a reduction in rate of relapse.[7,84] In addition, we and others have shown that patients who have a more robust post-HCT NK-cell recovery demonstrate reduced relapse rates.[85,86] Thus, we will provide an infusion of additional NK

cells from the haploidentical donor as a therapeutic cell product of purified NK cells (TC-NK).

Donors on this study will therefore undergo additional leukapheresis on (or around) patient day +5 to obtain the cells for the TC-NK. (See section 4.4). This will be 5 days after the completion of G-CSF mobilization and hematopoietic progenitor cell collection if a fresh stem cell graft is utilized. Donor WBC count is typically returning towards, but still slightly above, baseline at this timepoint.[87] Importantly, peripheral blood NK-cell quantities are not significantly altered with G-CSF mobilization and leukapheresis collection in healthy donors.[88] In addition, all three patients collected on HAP3R (using this schedule) have met target cell doses for the TC-NK products. The TC-NK will be isolated from the obtained donor mononuclear cells (MNC) in a two-step process. Initially, the MNC will be $CD3^+$ TCD followed by a $CD56^+$ NK enrichment using the CliniMACS immunomagnetic selection program. TC-NK have been generated using this procedure for the following protocols: NKHEM, INFT2, NKAML, and AML08. The average content of the first 50 TC-NK generated are listed in the table below. We are able to generate a therapeutic cell product that is highly purified for NK cells with minimal contamination of T cells. The final product volume will be approximately 10mL.

TC-NK	Average	Standard Deviation	Purity
NK dose (10^6 $CD56^+$ cells/kg)	26.6	± 22.6	96.5%.
T cell ($10^6 CD3^+$cells)	0.46	± 0.69	
T cell dose ($10^6 CD3^+$ cell/kg)	0.03	± 0.03	

To date, the infusion of TC-NK has been safe and tolerable in both the transplant and cell therapy setting. On INFT2, 13 patients received a TC-NK after haploidentical donor HCT and were evaluable for toxicity. No patient developed an acute infusion reaction to the infusion of TC-NK. One patient had NCI Grade 2 generalized edema the day following the TC-NK, however this is a known and common side effect of allogeneic HCT. Fifty-two patients that have received TC-NK in therapeutic cell therapy protocols were evaluable for toxicity at this writing. (25-NKAML, 19-NKHEM, 8-AML08) Some received TC-NK following a five-day clofarabine, etoposide, cyclophosphamide regimen, while others received TC-NK after fludarabine, cyclophosphamide therapy. No patient developed an acute infusion reaction. One patient developed hypertension that required a single dose of anti-hypertensive during the night after the infusion.

2.7 Minimal residual disease

Despite the fact that the vast majority of patients with high-risk leukemia are in clinical remission prior to HCT, relapse remains a primary cause of failure after HCT. Detection of minimal residual disease (MRD) and early intervention may improve the clinical outcome. Detection of leukemic cells that are below the limits of detection by standard morphologic examination allow early interventions when the patients are MRD positive but still in remission. By using flow cytometry and polymerase chain reaction (PCR) amplification of antigen receptor genes in tandem, investigators at our institution have been able to conduct MRD studies in 80 consecutive ALL cases.[89] Results of St. Jude institutional studies have

shown that detection of MRD by immunologic techniques at any point in the treatment course is a powerful predictor of relapse in children with ALL.[89-91] However, other studies suggest that eradication of all acute leukemia cells may not be a prerequisite for cure.[92,93]

Similar to conventional-dose therapy, controversy also exists on the implication of MRD in the setting of HCT. Unlike CML, there is a paucity of data on the natural history of AML and ALL patients who have MRD after HCT, and how pre-transplant MRD levels influence posttransplant outcomes.[94,95] It is unclear whether they are also at greater risk of relapse; and whether further pharmacological or immunologic therapy indeed prolongs survival and increases cure rates. Thus, for the participants who are enrolled in this protocol who are unable to proceed to posttransplant immunomodulatory protocols, we will gather the MRD information together with hematopoietic chimerism in a descriptive manner to study the relationship between MRD and chimerism in this large cohort of patients. The knowledge gained from this study should allow their future application to guide therapeutic interventions.

2.8 Rationale for present study

Many patients with high risk hematologic malignancies that would benefit from allogeneic HCT do not have an HLA-matched donor available in the necessary time. This institution has had success with the use of haploidentical donor HCT for such patients. In particular, when a homogenous patient population was examined, outcomes with haploidentical donor HCT appear to be similar to those of conventional allogeneic HCT.[10] Thus haploidentical HCT should be offered as a viable option to candidates with high-risk hematologic malignancy that lack a suitable HLA-matched donor, and have a suitable haploidentical donor.

2.9 Experience with GVHD prior to Amendment 2.0

The GVHD syndromes experienced by patients on this protocol differ from conventional Tcell-replete transplantation. Prior to amendment 2.0, 26 patients received naïve T-celldepleted HCT (range in survivors 180 – 736 days post-HCT). Seven patients developed grade II-IV acute GVHD (1 - II, 4 - III, 2 - IV). GVHD prophylaxis was sirolimus in the first 9 patients, and MMF for the remaining patients. The median duration of GVHD prophylaxis was 18 (range 0 – 154) days. One patient had upper GI symptoms a few months posttransplant in conjunction with a rash and was diagnosed with grade II acute GVHD. The six patients with severe (III-IV) GVHD presented more atypically. One patient initially developed diarrhea with frank blood. Endoscopy showed severe mucosal injury with a broad differential diagnosis, and her prophylactic MMF was stopped. One week later she had fever, an elevated CRP above 20mg/dL, and a new rash. She responded to infliximab and systemic steroids. A second patient developed hepatitis with an HHV6 viral load over 5 log. One week later she developed a rash and the bilirubin continued to rise, therefore systemic steroids were initiated. The third patient presented with fever, nausea, and a rise in liver transaminases followed by increased bilirubin. Diarrhea and rash followed a few days later with a rise in CRP to 15mg/dL. Systemic steroids and infliximab were initiated, and GVHD prophylaxis was changed from sirolimus to tacrolimus. Due to very aggressive malignancy at diagnosis, steroids were weaned aggressively in these three

patients as tolerated (courses were 65, 56, and 61 days respectively). GVHD prophylaxis was also stopped prior to Day +100 in all. Perhaps due to this aggressive weaning schedule, in these 3 patients, 1 patient developed recurrent acute GVHD and the other two developed chronic GVHD. The fourth patient was plagued by prolonged anorexia, and was never diagnosed with GVHD nor treated with prolonged systemic therapy for GVHD and yet recovered completely, however the consensus of extramural reviewers was that she had grade III acute GVHD. Another patient was diagnosed by the care team and GI consult as CMV enteritis, but external auditors suggested that isolated GI GvHD as a possible etiology, so the coding was changed to acute GvHD. The final patient was diagnosed as toxic epidermal necrolysis (TEN), but consensus of external reviewers felt that acute GvHD was the more likely etiology, therefore the coding was changed to acute GvHD. Of these 7 patients coded as grade II-IV acute GVHD, one died of ARDS and another of progressive disease, the rest remain alive as of December 2015.

Although the rate of moderate to severe acute GVHD was similar to the rate on the previous study HIFLEX, maraviroc was added in Amendment 3.0 in an attempt to reduce the incidence of significant gut and liver inflammation without further inhibiting T-cell activation and proliferation, which had the potential to reduce acute GVHD. However, 4 of 5 patients who received maraviroc developed acute GVHD grade III or IV; this is compared to only 7 patients developing GVHD grade III or IV in the 36 patients transplanted prior to the addition of maraviroc. Therefore, maraviroc use was discontinued. Prior to amendment 5.0, as of date April 4, 2018, preliminary analysis showed a significantly higher rate of GVHD grade III-IV in patients who received maraviroc (4 of 5 = 80%) compared to those patients who did not receive maraviroc (12 of 53 = 25%)(p = 0.02).

Prior to amendment 2.0, 8 of 26 patients (31%) have developed chronic GVHD (5 mild, 3 moderate by NIH criteria). Six of the 8 patients had oral involvement, with all other sites less commonly involved. Three have required systemic immunosuppressive therapy.

3.0 PROTOCOL ELIGIBILITY CRITERIA

3.1 Inclusion criteria for Transplant Recipient

3.1.1 Age less than or equal to 21 years.

3.1.2 Does not have a suitable HLA-matched sibling donor (MSD) or volunteer HLAmatched unrelated donor (MUD) available in the necessary time for stem cell donation, or is not a candidate for MSD or MUD HCT due to refractory disease.

3.1.3 Has a suitable single haplotype matched (≥ 3 of 6) family member donor.

3.1.4 High risk hematologic malignancy.

3.1.4.1 High risk ALL in CR1.

Examples include, but not limited to: t(9;22), hypodiploid, MRD >1% at the end of induction, M2 or greater marrow at the end of induction, Infants with MLL fusion or t(4;11).

3.1.4.2 ALL in High risk CR2.

Examples include, but not limited to t(9;22), BM relapse <36 mo CR1, TALL, very early (< 6mo CR1) isolated CNS relapse.

3.1.4.3 ALL in CR3 or subsequent.

3.1.4.4 AML in high risk CR1 (diagnosis of AML includes myeloid sarcoma).

Examples include but not limited to: preceding MDS, 5q-, -5, -7, FAB M6, FAB M7 not t(1;22), MRD > or = 5% on day 22 (AML08), MRD > 0.1% after two cycles of induction, M3 marrow after once cycle of induction, M2 marrow after two cycles of induction, FLT3-ITD.

3.1.4.5 AML in CR2 or subsequent.

3.1.4.6 AML in relapse with <25% blasts in BM

3.1.4.7 Therapy related AML, with prior malignancy in CR > 12mo

3.1.4.8 MDS, primary or secondary

3.1.4.9 NK cell, biphenotypic, or undifferentiated leukemia in CR1 or subsequent.

3.1.4.10 CML in accelerated phase, or in chronic phase with persistent molecular positivity or intolerance to tyrosine kinase inhibitor.

3.1.4.11 Hodgkin lymphoma in CR2 or subsequent after failure of prior autologous HCT, or unable to mobilize stem cells for autologous HCT.

3.1.4.12 Non-Hodgkin lymphoma in CR2 or subsequent after failure of prior autologous HCT, or unable to mobilize stem cells for autologous HCT.

3.1.4.13 JMML

3.1.4.14 One of the following hematologic malignancies that are refractory (includes chemoresistant relapse or primary induction failure).

- ALL
- AML
- CML (blast crisis)
- Hodgkin or non-Hodgkin lymphoma

3.1.5 If prior CNS leukemia, it must be treated and in CNS CR

3.1.6 Does not have any other active malignancy other than the one for which this HCT is indicated.

3.1.7 No prior allogeneic HCT, and no autologous HCT within the previous 12 months.

3.1.8 Patient must fulfill pre-transplant evaluation:

3.1.8.1 Left ventricular ejection fraction > 40%, or shortening fraction ≥ 25%.

3.1.8.2 Creatinine clearance (CrCl) or glomerular filtration rate (GFR) ≥ 50 ml/min/1.73m^2.

3.1.8.3 Forced vital capacity (FVC) ≥ 50% of predicted value; or pulse oximetry ≥ 92% on room air if patient is unable to perform pulmonary function testing.

3.1.8.4 Karnofsky or Lansky (age-dependent) performance score ≥ 50 (See APPENDIX A).

3.1.8.5 Bilirubin ≤ 3 times the upper limit of normal for age.

3.1.8.6 Alanine aminotransferase (ALT) ≤ 5 times the upper limit of normal for age.

3.1.8.7 Not pregnant. If female with child bearing potential, must be confirmed by negative serum or urine pregnancy test within 14 days prior to enrollment.

3.1.8.8 Not breast feeding

3.1.8.9 Does not have current uncontrolled bacterial, fungal, or viral infection.

3.2 Inclusion criteria for Haploidentical Donor

3.2.1 At least single haplotype matched (≥ 3 of 6) family member

3.2.2 At least 18 years of age.

3.2.3 HIV negative.

3.2.4 Not pregnant as confirmed by negative serum or urine pregnancy test within 14 days prior to enrollment (if female).

3.2.5 Not breast feeding.

3.2.6 Regarding eligibility, is identified as either:

3.2.6.1 Completed the process of donor eligibility determination as outlined in 21 CFR 1271 and agency guidance; <u>OR</u>

3.2.6.2 Does not meet 21 CFR 1271 eligibility requirements, but has a declaration of urgent medical need completed by the principal investigator or physician sub-investigator per 21 CFR 1271.

3.3 Gender and minorities

According to institutional and NIH policy, this study will accession patients regardless of gender and ethnic background. Institutional experience confirms broad representation in this regard. However, pregnant and lactating females are excluded from participation as the short and long-term effects of the preparative agents, study infusion (recipients) as well as the long-term effects of mobilization and apheresis procedure (donors) on a fetus and a nursing child through breast milk are not entirely known at this time.

4.0 TREATMENT PLAN

4.1 Preparative regimen

DAY	MEDICATION	DOSE	DOSE #
-9	Total Lymphoid Irradiation (TLI)	2Gy per fraction x 2 fractions	1,2 of 4
-8	Total Lymphoid Irradiation (TLI)	2Gy per fraction x 1 fraction	3 of 4
	Fludarabine	30 mg/m2 intravenous once daily	1 of 5
-7	Total Lymphoid Irradiation (TLI)	2Gy per fraction x 1 fraction	4 of 4
	Fludarabine	30 mg/m^2 intravenous once daily	2 of 5
-6	Cyclophosphamide	60 mg/kg intravenous once daily	1 of 1
	Fludarabine	30 mg/m^2 intravenous once daily	3 of 5
-5	Fludarabine	30 mg/m^2 intravenous once daily	4 of 5
-4	Fludarabine	30 mg/m^2 intravenous once daily	5 of 5
-3	Thiotepa	5 mg/kg intravenous twice daily	1,2 of 2
-2	Melphalan	70 mg/m^2 intravenous once daily	1 of 2
-1	Melphalan	70 mg/m^2 intravenous once daily	2 of 2
0	HPC,A Infusion(CD34+ selected)		
+1	HPC,A infusion (CD45RA depleted)		
+2-5	--		
+6	TC-NK Infusion		
+7	G-CSF	5mcg/kg subcutaneous or intravenous daily until ANC >2000 for 2 consecutive days, and then as clinically indicated	
+13	Mycophenylate mofetil	15 mg/kg intravenously three times daily.	

Cellular infusion procedures and monitoring:

For the proper infusion procedures and monitoring of the HPC product please refer to BMT&CT SOP 40.02 "Hematopoietic Progenitor Cell Infusion – FRESH (Allogeneic): IV Push and IV Drip" or SOP 40.03 "Hematopoietic Progenitor Cell Infusion – FROZEN: IV Push". For the TC-NK infusion, please see BMT&CT SOP 40.04 "Therapeutic Cell Infusion". Importantly, for TC-NK infusion, steroid pre-medications should not be given unless specified by an attending transplant physician. The TC-NK product will be infused by slow IV push over 3 to 15 minutes. Please note that all relevant SOPs can be found on the BMT&CT Clinical Transplant Program intranet page: http://home.web.stjude.org/bone_marrow/clinicalHome.shtml

Importantly, during the cellular infusions, monitoring of vital signs, breath sounds, heart rate, pulse oximetry, and I/O will be done per the established transplant nursing procedure, as well as appropriate Department of BMT&CT SOPs, then documented on the Cellular Product Infusion Record. If a reaction is suspected at any time during the infusion, the nurse will 1. Stop the infusion, 2. Notify the Attending Transplant Physician immediately, 3. NOT discard the product until physician orders are given. Proper documentation (symptoms of patient, vital signs, actions taken, outcome, and follow-up) will be completed in the Cellular Product Infusion Record.

General comments:

The Hematopoietic Progenitor Cell, Apheresis (HPC,A) infusion may be delayed by approximately 24 hours in order to accommodate progenitor cell collection with the donor, the Blood Donor Center and/or HAL as well as the research participant clinical condition.

The administration of the TC-NK infusion may also be adjusted in order to accommodate NK-cell collection with the donor, the Blood Donor Center and/or HAL as well as the research participant clinical condition. Depending on date of NK-cell collection, it will be acceptable for the TC-NK infusion to occur between and including days +5 and +9 post progenitor cell infusion. In the event that the date of NK-cell infusion is adjusted, the mycophenylate mofetil (MMF) would typically be similarly adjusted such that dosing is initiated seven days post NK-cell infusion.

The term “every” used in tables is an approximate term meaning that these medications noted will be administered approximately “every” 12 hours. The drug administration timing in the case of “every 12 hours” may be modified by approximately +/- 4 hours or as clinically indicated such as in the case of surgical procedures or to accommodate other necessary medication, blood product delivery, or procedures (such as a needed CT scan). The term “day” does not refer to an absolute calendar day. It refers to a general 24-hour period. Dosing for the medications cyclophosphamide, fludarabine, thiotepa, and melphalan may be modified for research recipients based upon actual body weight or adjusted ideal body weight when clinically indicated. Mesna will be administered for prevention of hemorrhagic cystitis from the medication cyclophosphamide. In general, mesna is administered at 15 mg/kg/dose prior to cyclophosphamide and at approximately 3, 6, and 9 hours after the cyclophosphamide infusion, to give a 1:1 ratio of mesna:cyclophosphamide. Mesna dose and administration schedule may vary based on physician recommendation.

Criteria for medication calculations based on body weight/body surface area and other medication related information can be found in the St. Jude Formulary http://www.crlonline.com/crlsql/servlet/crlonline or the St. Jude Dept of Pharmaceutical Sciences intranet website http://home.web.stjude.org/pharmaceutical_ser/drugInfo.shtml. Medication doses may be rounded to the nearest integer or to the nearest appropriate quantity when clinically or pharmaceutically indicated as per the MD and PharmD.

TLI will be given at 800cGy total dose in 4 fractions over 2 or 3 days. Fractions are given at a minimum of 6 hours apart from beam on to beam on. In order to facilitate

scheduling, the general procedure for administering TLI has been defined here and in the above table. It should be noted that TLI can be administered at any point during the preparative regimen prior to the progenitor cell infusion, and over as many days as needed to further accommodate scheduling issues in collaboration with the Division of Radiation Oncology and will not be considered a variance in protocol if the total dose of 800 cGy is met.

G-CSF will generally start on Day +7, but initial dose may be moved to an earlier or later day as to not interfere with NK cell infusion. G-CSF may also be dose adjusted or stopped early if rapid engraftment is considered to be potentially harmful to the patient (such as with engraftment syndrome) in the opinion of the PI or transplant care provider.

MMF for GVHD prophylaxis: MMF will start one week after the NK cell infusion. In the event NK cells cannot be given, MMF may start Day 0, or as soon as it is known that the NK cell graft is unavailable (if later than Day 0). MMF is typically given IV initially, but may be converted to oral dosing when the patient is tolerating oral medications. In the absence of GVHD, MMF will be discontinued approximately Day +60. However, this regimen may be modified as clinically indicated in the presence of other factors including but not limited to persistent neutropenia, mixed or decreasing donor chimerism, graft rejection/failure or persistent/recurrent malignancy. In the event the participant develops GVHD, treatment will be according to the SOPs of the St. Jude Department of BMT&CT.

4.2 Donor Selection

If more than one family member donor is acceptable, then donor selection will be based on the preference of the primary transplant attending. Factors in selection will include donorrecipient matching of CMV serology, donor-recipient red blood cell compatibility, degree of HLA matching, size of the potential donor, previous use as a donor, presence of donorspecific antibody, and overall health of the potential donor.

Donor eligibility for cell collection will be determined through the guidelines outlined in 21 CFR 1271 and the Guidance for Industry: Eligibility Determination for Donors of Human Cells, Tissues, and Cellular and Tissue-Based Products (HCT/Ps). Potential donors will undergo an initial screening process that will include at least a complete physical exam, history and testing for relevant communicable diseases. Physical exams to evaluate donor candidacy will be conducted by a non-Department of BMTCT physician (St. Jude or non-St. Jude). For subsequent therapeutic cell collection procedures, if a complete screening procedure has been performed within the previous 6 months, an abbreviated donor screening procedure may be used for these repeat donations. The abbreviated screening procedure must determine and document any changes in the donor’s medical history since the previous donation that would make the donor ineligible, including changes in relevant social behavior.

If a donor is determined to be ineligible, the donor is not automatically excluded. Part 21 CFR 1271.65 (b)(1)(i) allows use of ineligible donors who are first or second degree blood relatives. In this situation, the physician will document the necessity of using the ineligible donor by providing a statement of "Urgent Medical Need" as explained in the

21 CFR 1271.3 (u). The cell therapy products will be labeled as required in 21 CFR 1271.65 (b)(2). Recipients or their legal guardians will be informed of the use of an ineligible donor.

Please see Departmental SOP 30.05 "Determination of Eligibility and Suitability for Stem Cell and Therapeutic Cell Allogeneic and Autologous Donors" for additional information.

4.3 Donor Mobilization and Graft Collections

A G-CSF mobilized peripheral blood stem cell (PBSC) product (identified as HPC,A) is the preferred progenitor cell graft source. Our desired target goal will be 3-5 x 10^6 $CD34^+$ cells/kg. This number of cells will be necessary to provide an adequate graft, following the various ex vivo manipulations, for prompt reconstitution. Two days of collection are typically needed to achieve this goal. However, on rare occasions, additional days may be necessary. Donors will undergo a standard hematopoietic stem cell mobilization regimen consisting of 6 days of G-CSF given subcutaneously at 10 micrograms/kilogram. The graft will be collected by leukapheresis on days 5 and 6. The HPC product will typically be collected and infused fresh, however there may be patients or logistical situations that require the HPC product to be collected early, processed, and stored frozen. The NK-cell product will always be collected, processed, and infused fresh.

The decision to use a fresh versus frozen HPC will be made by the PI and/or primary transplant attending based on patient and donor factors, as well as potential scheduling conflicts.

)ONOR MOBILIZATION TIME LINE (if fresh HPC product)		
**DAYS**	**MEDICATION**	**APHERESIS**
Day -5	G-CSF 10 mcg/kg/day SC*	
Day -4	G-CSF 10 mcg/kg/day SC*	
Day -3	G-CSF 10 mcg/kg/day SC*	
Day -2	G-CSF 10 mcg/kg/day SC*	
Day -1	G-CSF 10 mcg/kg/day SC*	Apheresis for HPC graft
Day 0	G-CSF 10 mcg/kg/day SC*	Apheresis for HPC graft
Day +1-4		Rest day
Day +5		Apheresis for TC-NK

* G-CSF may be reduced if the donor's WBC is >75.0 x 10^6/mL

The dose of G-CSF may require modification based on the complete blood counts (CBC). If the donor's white blood count (WBC) is >75.0 x 10^6/ml the dose of cytokine administered will be reduced. The guidelines for dose modification can be found in the St. Jude Children's Research Hospital Department of BMT and CT SOP 30.06.00 "The practice for the evaluation, preparation and care of allogeneic and autologous donors

mobilized with growth factor." Ongoing updates of this document can be located at the following St. Jude intranet website: http://home.web.stjude.org/bone_marrow/

The daily leukapheresed volumes for PBSC collection is generally 3–4 total blood volumes based on $CD34^+$ cell counts. Two additional days of leukapheresis may be performed at the physician's discretion (no more than 4 total) to reach the cell dose target, however, this is expected to be rare.

The TC-NK will be collected by the same leukapheresis procedures as the HPC product. However the donor will not undergo a mobilization procedure for this product collection. NK cell product collection will be limited to one day. Collection will typically occur on day +5, but may occur anywhere from day +4 to day+8 depending on patient and donor factors.

Leukapheresis may be terminated early upon request of donor, or when deemed medically necessary per the judgment of the treating sub-investigator physician or principal investigator. All PBSC products will be collected as per Foundation for the Accreditation of Cellular Therapy (FACT) guidelines. Donors will be monitored during the period of the mobilization and leukapheresis procedure with appropriate laboratory evaluation (Appendix D).

If we are unable to collect the minimum dose of 2 x 10^6 $CD34^+$ cells/kg of recipient weight from the first donor, and the recipient has not yet initiated the preparative regimen, then an alternative family member may be used if he/she fulfills all the donor criteria described in section 3.2. If the donor is unwilling or unable to complete the mobilization process or leukapheresis procedure, a BM product may be used. The BM product will be processed using the same cell selection methodology on the CliniMACS device.

4.4 Graft Preparation

Graft evaluation and preparation will take place in the Human Applications Laboratory (HAL) in the Department of Therapeutics Production and Quality (TPQ) using established SOP.

The initial HPC product(s) will be TCD using the investigational CliniMACS device and CD34 Microbead reagent as directed by the manufacturer (Miltenyi Biotech). See section 5.2 for additional CliniMACS device information. Briefly, hematopoietic progenitor cells collected by apheresis (HPC,A) from the mobilized donor will be initially assessed in the HAL and stored overnight at 4°C. The next morning, the product will be washed to remove platelets and adjusted to an appropriate cell concentration for incubation with the CliniMACS CD34 Microbead reagent in the manufacturer provided media. The cells will be washed to remove unbound microbeads. These cells will be applied to the CliniMACS device and the enrichment will be performed using the program "CD34 Enrichment 2.1" as described by the manufacturer.

After enrichment is complete, the cells will be washed and resuspended in an infusion grade solution. The graft product will be enumerated and assessed for viable $CD34^+$ cell and $CD3^+$ T-cell content by flow cytometry. The processed HPC,A product will be infused fresh or frozen for future use after completion of release testing and evaluation.

Cryopreservation will be performed per SOPs of the Human Applications Laboratory. Target cell doses are listed in the following table:

HPC Graft	Target Dose	Minimum Dose	Maximum Dose
$CD34^+$ cells/kg	$\geq 2 \times 10^6$	2×10^6	50×10^6
$CD3^+$ cells/kg	$\leq 0.05 \times 10^6$	0.001×10^6	0.1×10^6

If the target dose is not achieved in a single collection, up to two additional collections will be performed for CD34+ enrichment (see Section 4.3). Once the target dose is obtained for the CD34+ enriched product, one additional day of apheresis will be performed. This HPC product will be processed for CD45RA+ depletion using the investigational CliniMACS device as directed by the manufacturer (Miltenyi Biotech). See section 5.2 for additional CliniMACS device information. Briefly, therapeutic cells collected by apheresis (HPC,A) from the mobilized donor will be initially assessed in the HAL and stored overnight at 4ºC. The next morning, the product will be washed to remove platelets and adjusted to an appropriate cell concentration for incubation with the CliniMACS CD45RA Microbead reagent in the manufacturer provided media. The cells will be washed to remove unbound microbeads. These cells will be applied to the CliniMACS device and the depletion will be performed using the "Depletion 3.1" software as described by the manufacturer. There is no target CD34+ dose for the CD45RA depleted product. Given that the previously infused CD34+ enriched product(s) will provide a minimum dose of 2×10^6/kg, once the CD45RA depleted product is infused, the patient is likely to have received $3\text{-}5 \times 10^6$ CD34+ cells/kg.

There is no CD3+ cell target dosing on the CD45RA+ depleted product, the goal of this depletion is a $\geq 2 \log_{10}$ depletion of CD45RA+ cells, however a depletion that does not achieve this target level may still be released to the patient as long as the maximum CD3+CD45RA+ dose is not exceeded. The maximum dose of CD3+CD45RA+ cells will be limited to no greater than 0.05×10^6/kg.

HPC Graft	Target Dose	Minimum Dose	Maximum Dose
$CD34^+$ cells/kg	$3\text{-}5 \times 10^6$(combined)	2×10^6(combined)	50×10^6(combined)
CD45RA+	$\geq 2 \log_{10}$ depletion (CD45RA+ depleted graft only)		
$CD3^+$CD45RA+ cells/kg	$\leq 0.05 \times 10^6$	N/A	0.05×10^6

In rare instances, the progenitor cell dose from the first day collection may be so high that it is feasible to split that product in two aliquots such that one aliquot may undergo CD34 enrichment, and the second aliquot may undergo CD45RA-depletion, thereby deriving both progenitor cell grafts from a single collection. This is allowed at the discretion of the PI, and both products may be infused on the same day when derived from a single collection.

The therapeutic NK-cell product (TC-NK) will be processed using the investigational CliniMACS device as directed by the manufacturer (Miltenyi Biotech). See section 5.2 for additional CliniMACS device information. Briefly, therapeutic cells collected by apheresis (TC,A) from the non-mobilized donor will be initially assessed in the HAL and stored overnight at 4ºC. The next morning, the product will be washed to remove

platelets and adjusted to an appropriate cell concentration for incubation with the CliniMACS CD3 Microbead reagent in the manufacturer provided media. The cells will be washed to remove unbound microbeads. These cells will be applied to the CliniMACS device and the depletion will be performed using the "Depletion 2.1" software as described by the manufacturer. In the second purification step, the CD3+ depleted product will be enriched for CD56+ cells using the CliniMACS immunomagnetic enrichment program 1.1 with the CliniMACS CD56 Microbead reagent.

For this protocol, our goal will be to infuse immediately after processing (Day +6) all the NK cells collected to give >2x10^6 CD56+ cells/kg of recipient body weight, but allowing for a CD3+ CD56- cell dose of no greater than 0.05x10^6/kg. The target doses for the NK-cell product are as follows:

TC-NK	Target Dose	Minimum Dose	Maximum Dose
$CD56^+$ cells/kg	> 2 x 10^6	0.1 x 10^6	400 x 10^6
$CD3^+$ cells/kg	≤0.05 x 10^6	N/A	0.05 x 10^6

4.5 Additional Progenitor Cell Graft Administration

Infusion of an additional HPC graft from the original or an alternative haploidentical donor may be performed for participants when clinically indicated for graft failure, poor immune reconstitution, or poor hematopoietic recovery. The use of and content of a conditioning regimen is left to the discretion of the PI and/or primary transplant attending such that the most appropriate therapy is chosen for the clinical situation.

The HPC graft will typically be obtained by apheresis (HPC,A) and be infused fresh. The target dose for this additional CD 34^+ infusion is ≥5 x 10^6 cells/kg. If the participant has quiescent or active BOOP, acute Grade III-IV GVHD, or any other reason that a severely T-cell depleted graft may be indicated, then a graft from the donor processed on the CliniMACS™ device using either $CD34^+$ selection (using established SOPs) or CD3+ depletion methodology may be utilized. The boost target dose for these patients is ≥10 x 10^6 $CD34^+$cells/kg with a $CD3^+$ cell/kg dose of ≤ 0.5 x 10^5 $CD3^+$ cells/kg.

4.6 Donor Lymphocyte Infusions

Donor lymphocyte infusions (DLI) may be given with or without preceding lymphodepleting chemotherapy. Recipients who are further out from transplant and with substantial lymphocyte engraftment may quickly tolerize the newly infused donor lymphocytes. In such patients, lymphodepleting chemotherapy may be needed for effective DLI activity. Regimen will vary based on patient condition, underlying disease, etc. Regimen will be determined by attending physician and/or PI, but in general would be low dose, expected to have minimal direct toxicity, and have limited or very transient effects on ANC. Regimens for lymphodepleting chemotherapy will be defined in a separate document (non-protocol treatment plan-NPTP) and will require additional consent to the NPTP.

4.6.1 Conventional Donor Lymphocyte Infusions

DLI may be administered from the original donor for decreased donor chimerism, serious viral reactivation or infection, or any evidence of disease.

- Decreased donor chimerism is defined as any single chimerism test that is not 100% donor
- Serious viral reactivation is defined as any virus detected in the blood by PCR, with a 1 log increase in viral load despite antiviral medication.
- Serious viral infection is defined as any infectious disease from an identified virus that has progressed in severity despite antiviral therapy (if available) or shows no improvement despite 1 week of antiviral therapy (if available)
- Any evidence of disease is defined as any flow cytometry, PCR, NGS (such as RNAseq), cytogenetic, or any other validated molecular testing that detects the presence of the original hematologic malignancy within the limits of that particular test.

The DLI may be collected as a whole blood unit donation or by leukapheresis. If the DLI is collected by standard phlebotomy, the volume to be collected would be approximately 300 ml whole blood. If the DLI is collected by leukapheresis, the amount to be processed would be approximately 2 total blood volumes.

Prior to administration of DLI, the immunosuppression should be withdrawn and the recipient should have no active GVHD. The initial dose will typically be 2.5 x 10^4 $CD3^+$/kg. Subsequent doses will be administered at approximately 2 to 4-week intervals with escalating doses of T cells if no moderate or severe GVHD occurs with the prior DLIs. The typical initial dose escalation for patients on this protocol is presented in the following table:

**CONVENTIONAL DLI DOSE AND SCHEDULE**		
**DLI**	**Dose(10^4 $CD3^+$/kg)**	**Comments**
Initial Dose	2.5	Approximately 2-4 week interval<br>If no moderate or severe GVHD
Dose #2	5	
Dose #3	10	

Although this algorithm will be appropriate for a majority, the treating transplant attending physician may alter the dose and/or interval of DLI based on response to previous DLI, the severity of the clinical situation, and the condition of the patient. There is a low threshold for providing an initial dose of 2.5 x 10^4 $CD3^+$/kg. DLI at this dose level have not been associated with GVHD. Therefore, DLI may be initiated for any chimerism less than 100% in a patient who is off immune suppression. The risk of GvHD increases with increasing doses. In the last 5 years, no patient has developed moderate or severe GVHD after haploidentical DLI dosed at 5 x 10^4 $CD3^+$/kg, though mild GVHD has occasionally occurred. Doses of 10 x 10^4 $CD3^+$/kg should be considered to carry a risk of severe GVHD. Therefore dose escalation to this level must incorporate the appropriate risk/benefit analysis by the treating transplant physician.

4.6.2 CD45RA-depleted donor lymphocyte infusions

Low dose conventional DLI has been found to be very effective for treatment of low level mixed chimerism, but it's efficacy in serious viral infections and recurrent disease (even at MRD levels) is poor. Although a median of 10^8 memory T-cells are adoptively transferred at the time of transplant on this study, and rapid donor immune reconstitution has been previously demonstrated, some patients may have recurrence or persistence of lymphopenia due to infections or post-transplant immunosuppressive drugs (such as corticosteroids). Therefore selected patients may benefit from additional adoptive transfer of donor memory cell populations in the post-transplant setting. This would include: 1. Patients with viral infections that are not responding to antiviral therapy or historically have a poor response to antiviral therapy; 2. Patients who have evidence of relapse, particularly molecular or MRD levels of relapse; 3. Patients who are severely lymphopenic, such as ALC <500/uL and/or T-cell count <300/uL. These patients will be allowed to receive CD45RA-depleted DLI in the following doses:

CD45RA-DEPLETED DLI DOSE AND SCHEDULE		
**DLI**	**Dose(10^6 $CD3^+$/kg)**	**Comments**
Initial Dose	0.1	Approximately 2-4 week interval<br>If no moderate or severe GVHD
Dose #2	1	
Dose #3	10	

The DLI may be collected as a whole blood unit donation or by leukapheresis. If the DLI is collected by standard phlebotomy, the volume to be collected would be approximately 300 ml whole blood. If the DLI is collected by leukapheresis, the amount to be processed would be approximately 2 total blood volumes. The collected donor lymphocytes would then undergo CD45RA depletion as decribed for the HPC,A graft (section 4.4).

The release criteria of the CD45RA-depleted DLI will include at least a ≥ 2 $\log_{10}$ depletion of CD45RA+ cells. The maximum dose of CD3+CD45RA+ cells will be limited to no greater than 0.05×10^6/kg for any CD45RA-depleted DLI.

Conventional DLI and CD45RA-depleted DLI cannot be given within 14 days of each other.

4.7 Quality Assurance of Cellular Products

Quality assurance for cell products is overseen by the TPQ Quality Assurance division, which authorizes release of all products. Only trained stem cell processors will process the cell products. A labeling and product tracking system is in place to ensure that the correct cells are infused into the research participant.

Assays of cell numbers and immunophenotyping will be performed both before cell processing and at critical stages of the process. These values will be recorded according to SOP of the HAL. All products will be tested for viability and sterility (culture and

Gram stain). Culture results are not available before infusion of cell products. If the gram stain is positive, the research participant/parent and/or guardian will be informed of this event and of the risks of proceeding prior to infusion. Positive results will be investigated as per the variance procedures of the HAL. The IRB and FDA will be notified, if at any time after infusion, cell product was determined to be contaminated.

5.0 MEDICATION AND DEVICE INFORMATION

5.1 Medications

Cyclophosphamide (Cytoxan)	
Source & Pharmacology	Cyclophosphamide is a nitrogen mustard derivative. It acts as an alkylating agent that causes cross-linking of DNA strands by binding with nucleic acids and other intracellular structures, thus interfering with the normal function of DNA. It is cell cycle, phase non-specific. Cyclophosphamide is well absorbed from the GI tract with a bioavailability of >75%. It is a prodrug that requires activation. It is metabolized by mixed function oxidases in the liver to 4-hydroxycyclophosphamide, which is in equilibrium with aldophosfamide. Aldofosfamide spontaneously splits into nitrogen mustard, which is considered to be the major active metabolite, and acrolein. In addition, 4hydroxycy-clophosphamide may be enzymatically metabolized to 4ketocyclophosphamide and aldophosfamide may be enzymatically metabolized to carboxyphosphamide that is generally considered inactive. Cyclophosphamide and its metabolites are excreted mainly in the urine. Dose adjustments should be made in patients with a creatinine clearance of <50 ml/min.
Formulation and Stability	Cyclophosphamide is available in vials containing 100, 200, 500, 1000 and 2000mg of lyophilized drug and 75 mg mannitol per 100 mg of cyclophosphamide. Both forms of the drug can be stored at room temperature. The vials are reconstituted with 5, 10, 25, 50 or 100 ml of sterile water for injection, respectively, to yield a final concentration of 20 mg/ml. Reconstituted solutions may be further diluted in either 5% dextrose or 0.9% NaCl containing solutions. Diluted solutions are physically stable for 24 hours at room temperature and 6 days if refrigerated, but contain no preservative, so it is recommended that they be used within 24 hours of preparation.

Supplier	Commercially available
Toxicities	Dose limiting toxicities of cyclophosphamide includes BM suppression and cardiac toxicity. Cardiac toxicity is typically manifested as congestive heart failure, cardiac necrosis or hemorrhagic myocarditis and can be fatal. Hemorrhagic cystitis may occur and necessitates withholding therapy. The incidence of hemorrhagic cystitis is related to cyclophosphamide dose and duration of therapy. Forced fluid intake and/or the administration of mesna decreases the incidence and severity of hemorrhagic cystitis. Other toxicities reported commonly include nausea and vomiting (may be mild to severe depending on dosage), diarrhea, anorexia, alopecia, immunosuppression and sterility. Pulmonary fibrosis, SIADH, anaphylaxis and secondary neoplasms have been reported rarely.
Route	Intravenous infusion

Fludarabine (Fludara)	
Source & Pharmacology	Fludarabine phosphate is a synthetic purine nucleoside analog. It acts by inhibiting DNA polymerase, ribonucleotide reductase and DNA primase by competing with the physiologic substrate, deoxyadenosine triphosphate, resulting in inhibition of DNA synthesis. In addition, fludarabine can be incorporated into growing DNA chains as a false base, thus interfering with chain elongation and halting DNA synthesis. Fludarabine is rapidly dephosphorylated in the blood and transported intracellularly via a carrier-mediated process. It is then phosphorylated intracellularly by deoxycytidine kinase to the active triphosphate form. Approximately 23% of the dose is excreted as the active metabolite in the urine (with dosages of 18-25 mg/m^2/day for 5 days). Renal clearance appears to become more important at higher doses, with approximately 41-60% of the dose being excreted as the active metabolite in the urine with dosages of 80-260 mg/m^2.
Formulation and Stability	Fludarabine is supplied in single-dose vials containing 50 mg fludarabine as a white lyophilized powder and 50 mg of mannitol. The intact vials should be stored under refrigeration. Each vial can be reconstituted by adding 2 ml of sterile water for injection resulting in a final concentration of 25 mg/ml. Because the reconstituted solution contains no antimicrobial preservative, the manufacturer recommends that it should be used within 8 hours of preparation. The solution should be further diluted in 5% dextrose or 0.9% NaCl prior to administration.
Supplier	Commercially available.

Toxicities	The major dose-limiting toxicity of fludarabine is myelosuppression. Nausea and vomiting are usually mild. Side effects reported commonly include anorexia, fever and chills, alopecia and rash. Neurotoxicity can be manifested by somnolence, fatigue, peripheral neuropathy, mental status changes, cortical blindness and coma and is more common at high doses. Neurotoxicity is usually delayed, occurring 21-60 days after the completion of a course of therapy and may be irreversible. Side effects reported less commonly include diarrhea, stomatitis, increased liver function tests, liver failure, chest pain, arrhythmias and seizures. Pulmonary toxicity includes allergic pneumonitis characterized by cough, dyspnea, hypoxia and pulmonary infiltrates. Drug induced pneumonitis is a delayed effect, occurring 3-28 days after the administration of the third or later course of therapy. Administration of corticosteroids usually results in resolution of these symptoms.
Route	Intravenous

G-CSF (Filgrastim, Neupogen)	
Source & Pharmacology	G-CSF (granulocytic colony stimulating factor), is a biosynthetic hematopoietic agent that is made using recombinant DNA technology in cultures of *Escherichia coli.* G-CSF stimulates production, maturation and activation of neutrophils. In addition, endogenous G-CSF enhances certain functions of mature neutrophils, including phagocytosis, chemotaxis and antibody--dependent cellular cytotoxicity.
Formulation and Stability	G-CSF is supplied in vials containing 300 mcg and 480 mcg of G-CSF at a concentration of 300mcg/ml. The intact vials should be stored under refrigeration. The vials can be left out of refrigeration for 24 hours, but should be discarded if left at room temperature for longer periods of time. G-CSF can be drawn up into tuberculin syringes for administration and stored under refrigeration for up to 7 days prior to usage. G-CSF can be further diluted for intravenous infusion in 5% dextrose. Do not dilute in saline---precipitate may form. If the final concentration of this product is <15 mcg/ml, it is recommended that albumin be added to a final concentration of 2mg/ml (0.2%) to minimize adsorption of the drug to infusion containers and equipment.
Supplier	Commercially available.

Toxicities	G-CSF causes marked leukocytosis. Adverse reactions reported commonly include bone pain, thrombocytopenia, diarrhea, nausea, rash, alopecia, fever, anorexia and pain or bruising at the injection site. Allergic reactions, MI, atrial fibrillation, and splenomegaly have been reported rarely. G-CSF is contraindicated in research participants with allergy to *E. coli* derived products.
Route	Intravenous or subcutaneous.
Melphalan (L-phenylalanine mustard, phenylalanine mustard, L-PAM, L-sarcolysin, Alkeran[â])	
Source & Pharmacology	Melphalan, a derivative of nitrogen mustard, is a bifunctional alkylating agent. Its chemical name is 4-[bis(2-chloroethyl)amino]-L-phenylalanine, and it has a molecular weight of 305.20. Melphalan is active against tumor cells that are actively dividing or at rest. Its cytotoxicity is thought to be due to inter-strand cross-linking with DNA, probably by binding at the N7 position of guanine. Melphalan is highly protein bound and does not penetrate well into the cerebral spinal fluid. Elimination half-life after intravenous administration in adults is approximately 75 minutes. Elimination appears to be primarily by chemical hydrolysis, but caution should be used in patients with renal impairment. Plasma concentrations of melphalan after oral administration are highly variable, possibly due to incomplete absorption, variable "first pass" hepatic metabolism or rapid
	hydrolysis. Area under the plasma concentration-time curves for orally administered melphalan is approximately 60% of intravenously administered melphalan in adult studies.
Formulation and Stability	Available as 2 mg tablets for oral administration. This medication is stable at room temperature until expiration date on the packaging. Intravenous formulation is supplied as 50 mg freeze dried glass vial. Each 50 mg vial is supplied in a carton containing a 10 ml vial of sterile diluent. Lyophilized melphalan should be stored at controlled room temperature and protected from light. Each vial is marked with its expiration date. The melphalan for injection must be reconstituted immediately prior to infusion by rapidly adding the contents of the diluent vial (10 ml) to the freeze dried powder with a 20 gauge or larger sterile needle and immediately shaking vigorously until a clear solution is obtained. This results in a 5 mg/ml solution. The dose should then be diluted in 0.9% NaCl for injection to a final concentration of not greater than 0.45 mg/ml. The resulting admixture should be infused over a minimum of 15 minutes. The infusion should be completed within 60 minutes of reconstitution. Do not refrigerate the reconstituted melphalan.
Supplier	Commercially available

Toxicities	Melphalan is cytotoxic and caution should be used in handling and preparing the solution or administering the tablets. Use of gloves is recommended, and if contact with skin or mucosa occurs, immediately wash thoroughly. Second cancers such as acute non-lymphocytic leukemia, myeloproliferative syndrome, and carcinoma have been reported in patients taking melphalan alone or in combination with other chemotherapy or radiation. Melphalan causes suppression of ovarian function in premenopausal women, with a significant number of patients having amenorrhea. Testicular suppression (reversible and irreversible) has been reported. The most common adverse reaction is myelosuppression. Irreversible BM failure has been reported. Gastrointestinal side effects reported include nausea/vomiting, diarrhea and oral mucosa ulceration. Hepatic toxicity has occurred, including veno-occlusive disease. Acute hypersensitivity reactions occur in about 2.4% of patients, and can include anaphylaxis. Hypersensitivity reactions were characterized by urticaria, pruritus, and edema. Some patients exhibited tachycardia, bronchospasm, dyspnea and hypotension that responded to antihistamines and corticosteroids. Other side effects that have been reported include skin ulceration or necrosis at injection site, vasculitis, alopecia, hemolytic anemia, pulmonary fibrosis, and interstitial pneumonitis.
Route	Intravenous

Mesna (Mesnex)	
Source & Pharmacology	Mesna is a synthetic sulfhydryl (thiol) compound. Mesna contains free sulfhydryl groups that interact chemically with urotoxic metabolites of
	oxaza-phosphorine derivatives such as cyclophosphamide and ifosfamide. Oral bioavailability is 50%. Upon injection into the blood, mesna is oxidized to mesna disulfide, a totally inert compound. Following glomerular filtration, mesna disulfide is rapidly reduced in the renal tubules back to mesna, the active form of the drug. Mesna and mesna disulfide are excreted primarily via the urine.
Formulation and Stability	Mesna is available in 2 ml, 4 ml and 100 ml amps containing 100 mg/ml of mesna solution. The intact vials can be stored at room temperature. Mesna may be further diluted in 5% dextrose or 0.9% NaCl containing solutions. Diluted solutions are physically and chemically stable for at least 24 hours under refrigeration.
Supplier	Commercially available

Toxicities	Mesna is generally well tolerated. Nausea and vomiting, headache, diarrhea, rash, transient hypotension and allergic reactions have been reported. Patients may complain of a bitter taste in their mouth during administration. Mesna may cause false positive urine dipstick readings for ketones.
Dosage and Administration	Mesna is generally dosed at approximately 25% of the cyclophosphamide dose. It is generally given intravenously prior to and again at 3, 6 and 9 hours following each dose of cyclophosphamide.
Route	Intravenous
Thiotepa (Thioplex by Immunex) (TESPA, TSPA)	
Source & Pharmacology	Thiotepa is a cell-cycle nonspecific polyfunctional alkylating agent. It reacts with DNA phosphate groups to produce cross-linking of DNA strands leading to inhibition of DNA, RNA and protein synthesis. Thiotepa is extensively metabolized in the liver to metabolites that retain activity, primarily triethylene-phosphoramide (TEPA). The main route of elimination is via the urine, mainly as metabolites; the elimination halflife of the thiotepa is 2.5 hours, and that of TEPA is 17.6 hours.
Formulation and Stability	Thiotepa is supplied in single-use vials containing 15 mg of lyophilized thiotepa, 80 mg NaCl and 50 mg NaHCO3. The intact vials should be stored under refrigeration and protected from light. Each vial should be reconstituted with 1.5 ml of sterile water for injection to yield a concentration of 10 mg/ml. Further dilution with sterile water for injection to a concentration of 1 mg/ml yields an isotonic solution; if larger volumes are desired for intracavitary, intravenous infusion, or perfusion therapy, this solution may then be diluted with 5% dextrose or 0.9% NaCl containing solutions. The 10 mg/ml reconstituted solution is chemically stable when stored in the refrigerator for up to 5 days, however, it is recommended that solutions be prepared just prior to administration since they do not contain a preservative. Reconstituted solutions should be clear to slightly opaque: the solutions may be filtered<br>through a 0.22 micron filter to eliminate haze.
Supplier	Commercially available; manufactured by Immunex
Toxicities	Dose limiting toxicity is myelosuppression. The leukocyte nadir may occur at any time from 10 to 30 days. Other toxicities include pain at the injection site, nausea and vomiting, anorexia, mucositis, dizziness, headache, amenorrhea, interference with spermatogenesis, and depigmentation with topical use. Allergic reactions, including skin rash and hives, have been reported rarely. Rare cases of apnea, hemorrhagic cystitis, and renal failure have occurred. Thiotepa is mutagenic, carcinogenic, and teratogenic in animals. Pregnancy category D.
Route	Intravenous infusion.

Mycophenolate mofetil (MMF, CellCept[])	
Source & Pharmacology	Mycophenolate mofetil is hydrolyzed to mycophenolic acid (MPA), an immunosuppressive agent. MPA inhibits T- and B-cell proliferation, Tcell synthesis, and antibody secretion by potent, noncompetitive reversible inhibition of inosine monophosphate dehydrogenase (IMPDH) in the purine biosynthesis pathway. Inhibition of IMPDH results in a depletion of guanosine triphosphate and deoxyguanosine triphosphate, important intermediates in the synthesis of lymphocyte DNA, RNA, proteins and glycoproteins. Oral formulations of mycophenolate mofetil are rapidly and extensively absorbed when given on an empty stomach. Food and aluminum- and magnesium-containing antacids decrease absorption of mycophenolate mofetil. MMF is rapidly hydrolyzed to the active metabolite (MPA) after oral or intravenous administration. Free MPA is conjugated in the liver by glucuronyl transferase to inactive mycophenolic acid glucuronide (MPAG) which is excreted in the urine and feces. Time to peak plasma concentration is 0.8 – 1.3 hours, and the mean elimination half-life is 17.9 hours. Enterohepatic recirculation of MPA contributes to plasma concentrations. Administration of cholestyramine interrupts the enterohepatic recirculation and can decrease bioavailability by as much as 40%. Patients with renal insufficiency have increased plasma concentrations of MPA and MPAG. Acyclovir and ganciclovir may compete with MPAG for renal tubular secretion, resulting in increased plasma concentrations of both drugs.
Formulation and Stability	Mycophenolate mofetil is commercially available as 250 mg capsules, 500 mg tablets, 200 mg/ml powder for oral suspension, and 500 mg vials of powder for injection.
Supplier	Commercially available
Toxicities	Adverse events seen in patients taking mycophenolate mofetil include hypertension, hypotension, peripheral edema, leukopenia, anemia, thrombocytopenia, hypochromic anemia, leukocytosis, headache, insomnia, dizziness, tremor, anxiety, paresthesia, hyperglycemia, hypercholesterolemia, hypokalemia, hyperkalemia, hypophosphatemia, diarrhea, constipation, nausea, vomiting, anorexia, abdominal pain, dyspepsia, urinary burning or frequency, renal tubular necrosis, hematuria, increase serum creatinine and BUN, a variety of infections due to immunosuppression, rash, acne, ocular changes (cataracts, blepharitis, keratitis, glaucoma, and macular abnormalities) occasional leg cramps or pain, bone pain, myalgias, and hand cramps. Intravenous infusions have been reported to cause thrombosis and phlebitis. There have been occasional reports of gastrointestinal hemorrhage. High dose therapy with mycophenolate in adults with psoriasis has been associated with the following neoplasms: adenocarcinoma of the breast and colon, basal cell carcinoma, carcinoma of the gallbladder, histiocytic lymphoma, glioblastoma multiforme, and squamous cell carcinoma of the epiglottis.
Route	Intravenous or oral

5.2 CliniMACS™ System

The mechanism of action of the CliniMACS Cell Selection System is based on magneticactivated cell sorting (MACS). The CliniMACS device is a powerful tool for the isolation of many cell types from heterogeneous cell mixtures, (e.g. apheresis products). These can then be separated in a magnetic field using an immunomagnetic label specific for the cell type of interest, such as $CD3^+$ human T cells.

The cells to be isolated are specifically labeled with super-paramagnetic particles by an antibody directed toward a cell surface antigen. After magnetic labeling, the cells are separated using a high-gradient magnetic separation column as described below. The magnetically labeled cells are retained in the magnetized column while the unlabeled cells flow through the column for collection. The retained cells are eluted by removing the magnetic field from the column, washing the cells out and collecting them in a separate container from the unlabeled cells.

The super-paramagnetic particles are small in size (about 50 nm in diameter) and are composed of iron oxide/hydroxide and dextran conjugated to monoclonal antibodies. These magnetic particles form a stable colloidal solution and do not precipitate or aggregate in magnetic fields. The antibody conjugated beads used in this system are highly specific (e.g. CD3+ cells via OKT3 conjugated beads). High-gradient MACS technology has been shown to achieve rapid and highly specific depletion or enrichments of large numbers of target cells from BM, cord blood, and normal peripheral blood mononuclear cells.

The CliniMACS device incorporates a strong permanent magnet and a separation column with a ferromagnetic matrix to separate the cells labeled with the magnetic particles. The high-gradient system allows the application of strong magnetic forces and a rapid demagnetization. Small ferromagnetic structures, such as the column matrix, placed in a magnetic field concentrate this homogenous field and thereby produce high magnetic gradients. In their immediate proximity, the ferromagnetic structures generate magnetic forces 10,000-fold greater than in the absence of those structures enabling the retention of magnetically labeled cells. After removing the column from the magnet, the rapid demagnetization of the column matrix allows the release of retained cells.

The CliniMACS device is comprised of a computer controlled instrument incorporating a strong permanent magnet, a closed-system sterile tubing set containing columns with a coated ferromagnetic matrix and a paramagnetic, cell specific, labeling reagent. The instrument will separate the cells in a fully automated process yielding a cell population highly depleted of $CD3^+$ cells. The CliniMACS device is not licensed by the FDA and therefore is investigational.

The CliniMACS device has separate programs that allow cell selection procedures optimized for either depletion (e.g. $CD3^+$ or $CD45RA^+$) or selection of a target cell population (e.g. $CD34^+$ or $CD56^+$ cells). The basic mechanism is the same for either application; target cells are "tagged" with super-paramagnetic particles and eventually separated from the unlabeled cells using the CliniMACS device as described above. The desired target cells can either be infused or discarded appropriately.

6.0 REQUIRED OBSERVATIONS AND EVALUATIONS

6.1 Pre/peri/post-transplant evaluations

All pre/peri/posttransplant and long-term follow-up evaluations for these participants will be carried out as outlined in Appendix D, and guided by the Standard Operating Procedures (SOPs) of the St. Jude Children's Research Hospital, Department of BMTCT, for recipients of allogeneic stem cell transplantation. Copies of these SOPs and ongoing updates can be found at the following site: http://home.web.stjude.org/bone_marrow/clinicalHome.shtml.

Furthermore, to accommodate the research studies, flexibility in the date is allowed without a deviation from protocol. The degree of flexibility in the timing is also provided in Appendix
E.

6.2 Long-term follow-up evaluations

In general, recipients of allogeneic HCT at St. Jude are seen at least annually until 10 years posttransplant in the Department of BMTCT outpatient clinic. For the purpose of this study, research participants will be followed to year 1 post-transplantation. All study participants will be eligible for enrollment in the institutional long-term follow-up protocol for children and young adults who have received stem cell transplantation at St. Jude Children's Research Hospital (BMTFU protocol).

6.3 Evaluation for Chimerism and Engraftment

Evaluation for chimerism and engraftment will be performed on bone marrow or peripheral blood samples according to the timelines noted in Appendix E. Bone marrow chimerism studies will be conducted on or about the following time points: day +21, day +100, and year one posttransplant. However, for research participants who have less than 100% donor chimerism at or about day +21 posttransplant, a repeat bone marrow study (to include chimerism) may be performed approximately one week after the initial procedure.

The time to neutrophil and platelet engraftment will be recorded. Neutrophil engraftment will be defined as the first of 3 consecutive days of an absolute neutrophil count (ANC) greater than or equal to 500/mm^3. Time to platelet engraftment will be designated as the time to platelet count exceeding 20,000/mm^3 and 50,000/mm^3 without a platelet transfusion in the preceding seven days.

Chimerism studies using peripheral blood will be obtained on an approximate weekly basis according to the evaluation schedule noted in Appendix E. Additional bone marrow and/or peripheral blood chimerism studies may be performed throughout the course of this study when clinically indicated. Chimerism studies derived from bone marrow may be used in lieu of a specified peripheral blood sample if a bone marrow sample is available. In the event of graft failure/rejection, subsequent chimerism studies may be held as they would not be clinically indicated at that time.

If there is an initial decrease in donor chimerism to less than 90% at any time on peripheral blood studies, a bone marrow examination will subsequently be performed

within approximately two weeks to confirm this initial decline. In addition, chimerism analysis may be performed in subsets of lymphocytes, granulocytes, and monocytes for research participants with increasing host chimerism until the research participant attains 100% donor chimerism. Chimerism studies will be performed in the St. Jude Department of Pathology using standard DNA techniques [i.e. VNTR (variable number tandem repeat)] and/or FISH (fluorescent in-situ hybridization) analysis. Chimerism studies will be reported in the database as donor percentages.

6.4 Evaluation for immune reconstitution

Research participants will have immune reconstitution studies of lymphocyte subsets (i.e. cells, B cells, and NK cells). These studies may be performed more often than what is outlined in Appendix E depending on the clinical status of the research participant.

Immune analysis as described (Mnemonic listed in 6.4.1 through 6.4.4) will be performed according to the schedule outlined in Appendix D until the immune parameters recover to normal level or donor pattern:

6.4.1 Lymphocyte subsets study: Flow cytometry enumeration.

6.4.2 VBETA/TREC Research: Thymic output and T-cell repertoire.

6.4.3 Lymphocyte Phenotypes Research: T-cell and NK-cell number and function.

6.4.4 Quantitative immunoglobulins: IgG, IgM, and IgA levels.

6.4.5 IR-Phenotype: Immune reconstitution of memory and naïve T cells will be investigated in depth in the Youngblood Lab. This may include phenotypic subset characterization, functional correlates, and analysis of the epigenetic signatures of these populations.

6.4.6 T-Function: T-cell function will be investigated in depth in the Thomas Lab. This may include antigen-specific T-lymphocyte response to viral infections, such as herpes viruses (CMV, HSV and VZV) and respiratory viruses (influenza and RSV).

6.5 General Viral Surveillance

Serial PCR testing for CMV, adenovirus, and EBV will be done weekly to day 100 and then on an as needed basis. In research participants with progressive or active infection, samples may be obtained more frequently (St. Jude test mnemonic BMTPCR).

6.6 Minimal residual disease evaluation

Minimal residual disease (MRD) assays in peripheral blood and/or BM by immunologic and molecular methods will be performed for those research participants who have had this test performed during prior therapy for their disease at St. Jude only or those for who samples of diseased marrow were available to identify a leukemic marker for MRD testing. BM (3ml) and/or peripheral blood (5-10 ml) will be obtained from the participants at pre-transplant evaluation, at or about day +21, +100 and at the annual evaluation. MRD assays may be performed more frequently in participants with

increasing host chimerism. We will apply immunologic and molecular methods as previously described.[54-55] Tests will be performed in the appropriate St. Jude laboratories.

6.7 Research tests on haploidentical donor (optional)

Donors will be offered the option for participation in research studies of immune reconstitution. These tests will be obtained after consent and preferably prior to growth factor administration. Lymphocyte subset analysis of the donor appears to allow for the prediction of the reconstitution of the lymphocyte subsets in the research participant after transplantation. Data in larger donor/research participant pairs will help to verify these observations. A list of these optional research studies are noted in Appendix D and detailed below:

6.7.1 Lymphocyte subsets study

Flow cytometry enumeration.

6.7.2 VBETA/TREC Research

Thymic output and T-cell repertoire.

6.7.3 Lymphocyte Phenotypes Research

Lymphocyte number and function.

6.7.4 IR-PHENOTYPE, and T-FUNCTION: In depth characterization of donor lymphocytes

7.0 EVALUATION CRITERIA

7.1 Adverse event monitoring

Adverse event (AE) monitoring for on-study research participants will be assessed using the NCI Common terminology Criteria for Adverse Events Version 3.0. The specific criteria for adverse event monitoring are noted Section 9.0 and in Appendix D.

7.2 GVHD diagnosis and grading

Acute and chronic GVHD will be evaluated, staged, and graded using the criteria found in Appendix B and C respectively.

Appendix B contains the COG stem cell committee consensus guidelines for establishing organ stage and overall grade of acute GVHD. In the initial 26 patients treated on this protocol prior to amendment 2.0, there were 3 patients initially diagnosed with hyperimmune response syndrome, or similar. This was due to the development of GVHD-like symptoms in some patients that were atypical of acute GVHD syndromes seen in conventional T-cellreplete transplantation. Other patients developed symptoms that were somewhat atypical, but also had a high degree of overlap with acute GVHD. Comprehensive review of the patients transplanted on HAPNK1 prior to amendment 2 was performed by institutional monitors, at least 3 external transplant physicians, and the PI. All patients previously noted to have hyperimmune response syndrome (or similar) were re-coded to acute GVHD. One additional patient was determined to have

significant GVHD by audit consensus, giving 4 of the first 26 patients with acute GVHD ≥ grade III. Although we and others[96] have noted that GVHD syndromes appear to be biologically different in Naïve T-cell depleted transplantation such as this, with amendment 2.0 any GVHD-like syndrome will be coded as GVHD until such time that a more specific syndrome is fully characterized. The COG stem cell committee consensus guidelines for establishing organ stage and overall grade of acute GVHD has been adopted as the standard GVHD diagnostic guidelines for the Department of BMTCT, and will be applied to patients on this protocol (see Appendix B). In addition, acute GVHD will be assessed at least once a week for the first 100 days per BMTCT SOP 20.01.

Other disorders that involve donor immune dysregulation, that are distinct from GVHD are known to occur in transplant recipients. Interstitial pneumonitis syndrome (IPS) and bronchiolitis obliterans organizing pneumonia (BOOP) are two relatively common examples. These disorders are often associated with acute GVHD, but are not considered to be acute GVHD, and are often treated with steroids. These and similar disorders will be captured as
AE's and will not be considered to be acute GVHD. The risk of non-GVHD disorders of immune dysregulation in naïve T-cell depleted transplantation is unknown. In the 26 patients transplanted on this study prior to amendment 2, no patient has been diagnosed with IPS or BOOP. However, 3 patients have been diagnosed with toxic epidermal necrolysis (TEN) (n=2) or Steven-Johnson syndrome (SJS) (n=1). TEN and SJS are considered to be variants of a disease continuum that is often triggered by viral infection or drug exposure, and is mediated by cytotoxic T cells.[97] All three patients experienced resolution of their TEN/SJS with brief immunosuppressive therapy.[98] It can be difficult due to overlapping symptoms, but given that therapeutic recommendations are different, it is important to distinguish such diseases from acute GVHD if possible. Any toxicity that fits criteria for a well described disorder of immune dysregulation (such as the examples above) will be recorded no matter the CTC CAE grade. This allows a full accounting of the incidence of these disorders in naïve T-cell depleted transplantation.

Appendix C contains a summary of the NIH consensus development project on criteria for clinical trials in chronic GVHD.[99] This table will be used for staging/grading of chronic
GVHD.

7.3 Performance status

Performance status will be assessed by Karnofsky/Lansky Performance Scores found in Appendix A.

7.4 Hematologic recovery

Posttransplant hematologic recovery will be determined using the engraftment criteria as follows:

7.4.1 Neutrophil engraftment will be defined as the first of 3 consecutive tests performed on different days of an ANC ≥ 500/mm^3 with evidence of donor cell engraftment.

7.4.2 Platelet engraftment will be defined as the first of 3 consecutive tests performed on different days of a platelet count ≥ 20,000/mm^3 with no platelet transfusions in the preceding 7 days.

7.5 Graft failure

Primary graft failure will be defined as an ANC never meeting or exceeding 500/mm^3 for 3 consecutive tests performed on different days and no evidence of donor chimerism (<5%) before day +42 post-HCT.

Secondary graft failure will be defined as a decline in ANC to<500/mm^3 with a decline in donor chimerism to <5% in research participants with prior engraftment.

7.6 Chimerism

Mixed hematopoietic chimerism will be defined as between 10% and 95% donor chimerism in the absence of immunosuppressive therapy.

8.0 OFF-STUDY CRITERIA

8.1 Recipient criteria

Recipient research participants will remain on study until one of the following occurs:

8.1.1 Withdrawal from protocol.

8.1.2 Death.

8.1.3 Donor unable to provide the HSC required for intended recipient to undergo the primary HCT procedure (refer to donor off-study criteria).

8.1.4 Unable to be contacted and/or effectively monitored by the Principal Investigator (PI) and/or designees for follow-up (lost to follow-up).

8.1.5 One year after HSC infusion (i.e. has completed the year +1 post-primary transplant evaluation) and enrollment onto the BMTFU protocol.

8.1.6 Development of a significant change in health status at any point of therapy which would render receipt of the transplantation procedure or continuation in the study medically unsafe or not in the participant's best interest.

8.1.7 Requires additional chemotherapy for morphologically confirmed disease relapse (generally >5%).

Epigenetic or targeted therapy and low dose lymphodepleting chemotherapy with DLI, even if molecularly detectible disease is present, would not trigger this off-study criteria.

8.2 Additional notes

Recipient research participants enrolled on this study will remain on study for monitoring if one of the following occurs:

8.2.1 Experiences graft failure/rejection.

8.2.2 Noncompliance with protocol medications/administrations and/or required follow-up evaluations.

8.2.3 Positive pregnancy test post-HSC infusion.

8.2.4 Recipient requires an additional HSC infusion, and unable to receive these cells due to donor issues.

8.3 Donor Criteria

Donor research participants will remain on study until one of the following occurs:

8.3.1 Withdrawal from protocol. Donor research participants may withdraw their consent to participate at any time. Physician may withdraw donor at any time that continuation in the study is deemed medically unsafe or no longer in the donor's best interest.

8.3.2 Day of transplant recipient or transplant donor death, whichever occurs first.

8.3.3 Development of a change in health status, including positive pregnancy test or a clinically significant risk for or positive testing for communicable disease, which in the opinion of the PI, would render the donor medically ineligible to serve (or continue to serve) as a therapeutic cell donor.

8.3.4 Unable to be contacted and/or effectively monitored by PI and/or designees for follow-up as judged by the PI (including non-compliance and lost to follow-up).

8.3.5 Once the PI has determined that the transplant recipient does/will not require an additional infusion(s) of the donor's cells for the purpose of this protocol, the donor will be taken off study seven days post final cell collection procedure.

9.0 REPORTING CRITERIA AND CONTINUING REVIEW REPORTS

9.1 Reporting Adverse Experiences and Deaths to St. Jude IRB

The principal investigator is responsible for promptly reporting to the IRB any adverse events that are unexpected/unanticipated, serious, and that may represent potential harm or increased risk to research participants. When an unexpected death occurs, the PI should report it to the Director of Human Subject's Protection immediately, by phone: (901) 5954359, cell: (901) 336-2894, fax: (901) 595-4361, or e-mail: hsp-1@stjude.org. A reportable event entry into TRACKS should follow within 48 hours of notification of the event.

Serious, unexpected, and related or possibly related events must be reported within 10 business days of notification of the event. At the same time, the investigator will notify the study sponsor and/or the FDA, as appropriate. All other SAEs, including expected death, and all captured AEs will be reported to the IRB at the time of the continuing reviews, with the following exceptions:

- Any grade III-IV infusion reactions will be reported as soon as possible but every effort should be made to assure reporting is no more than within 10 business days of the event.
- Any episodes of overall grade III or IV acute GVHD in participants will be reported to the IRB as soon as possible but no more than within 10 business days of the PI's confirmation of the diagnosis/grade of the event.

For this research study, recipient participants will be followed for all NCI Grade III-V adverse events from the start of conditioning and throughout the first year post HCT, regardless of their relationship to the treatment given. In addition, clinically significant NCI Grade I-II adverse events that are judged to be related/possibly related may be collected per the discretion and judgment of the PI. Examples of "clinically significant Grade I-II adverse events could include, but are not limited to: events meeting criteria for SAE, infections requiring oral systemic therapy, VOD or hemorrhagic cystitis. GVHD events will be recorded on an ongoing basis regardless of stage or grade using the criteria defined in Appendix B, and will not be graded according to NCI criteria.

With regard to the haploidentical donor participants, they will be followed for all SAEs and any clinically significant AEs, per the judgment of the PI, that are deemed related to the mobilization and/or apheresis procedure from the time of apheresis of NK cells, on through the initiation of mobilization growth factors, until 7-days post last day of the final apheresis procedure. If the transplant recipient requires a second HSC infusion, meaning that the donor is required to undergo the mobilization and apheresis procedure again, collection of this donor safety data will restart upon the initiation of the subsequent mobilization procedure and continue until 7-days post the last day of this apheresis procedure. Timelines for reporting of these donor events to the institutional and federal governing agencies will be according to the same timelines utilized for the recipients. A listing of the captured donor safety data will be provided in a separate table from the transplant recipients within each respective continuing review report.

The following definitions apply with respect to reporting adverse experiences:

Serious adverse event – any adverse event temporally associated with the participant's participation in research that meets any of the following criteria:

- results in death;
- is life-threatening (places the participant at immediate risk of death from the event as it occurred);
- requires inpatient hospitalization or prolongation of existing hospitalization
- results in a persistent or significant disability/incapacity;
- results in a congenital anomaly/birth defect; or
- any other adverse event that, based upon appropriate medical judgment, may jeopardize the participant's health and may require medical or surgical intervention to prevent one of the above outcomes.

Unexpected adverse event – any adverse event meeting any of the following criteria:

- an event for which the specificity or severity is not consistent with the protocol related documents, including the applicable investigator brochure, IRB approved consent form, IND/IDE application or any of the product labeling or package inserts;
- an event for which the observed rate of occurrence is significantly increased above what is expected or credible baseline rate for comparison;
- an event for which the occurrence is not consistent with the expected natural progression of any underlying disease, disorder, or condition of the participant(s)

experiencing the adverse event and the participant's predisposing risk factor profile for the adverse event.

The principal investigator is responsible for reviewing the aggregate toxicity reports and reporting to the IRB if the frequency or severity of serious toxicities exceed those expected as defined in the protocol or based on clinical experience or the published literature. Any proposed changes in the consent form or research procedures resulting from the report are to be prepared by the study team and submitted with the report to the IRB for approval.

9.2 Reporting to St. Jude Institutional Biosafety Committee

Continuing review reports will be sent to the Institutional Biosafety Committee (IBC) on at least an annual basis using the most current version of the continuing review form found on the IBC website. The safety reports, sent to the IRB for both the donors and stem cell recipients, will be simultaneously forwarded to the IBC. Therefore, reporting for safety events to this committee will be according to the same timelines as reporting to the IRB. This includes notification of achievement of MTD (if/when applicable). As per the direction of the IBC, only those protocol revisions and amendments directly related to the CliniMACS processing and related reagent(s) will require review and consideration by the IBC. Other revisions/amendments will be noted in the IBC continuing review report.

9.3 Reporting to FDA (21CFR§312.32 Safety reports)

The FDA will be notified in writing (IDE safety report) of any serious and unexpected AE associated with an investigational treatment or device; or any results from laboratory animal tests that suggest a significant risk for human subjects including reports of mutagenicity, teratogenicity, or carcinogenicity.

Each notification to the FDA should be made as soon as possible and no later than 15 calendar days after the sponsor's initial receipt of the information. The FDA may require additional data to be submitted. In each written IND safety report, the sponsor shall identify all safety reports previously filed with the IND concerning a similar adverse experience, and shall analyze the significance of the adverse experience in light of the previous, similar reports where applicable.

The sponsor shall also notify the FDA by telephone or by facsimile transmission of any unexpected fatal or life-threatening experience associated with the use of the investigational device as soon as possible but no later than 7 calendar days after the sponsor's initial receipt of the information. Any grade III-IV infusion reactions will be reported as soon as possible but every effort should be made to assure reporting is no more than within 7 business days of the event. Follow-up information to a safety report must be submitted as soon as the relevant information is available.

If the results of further investigation show an AE that was not initially determined to be reportable should later be deemed reportable, the sponsor shall inform the FDA of the event in a written safety report as soon as possible, but no later than 15 calendar days after the determination is made. Results of the investigation of other safety information

shall be submitted, as appropriate, in an information amendment or annual report. Continuing review reports, which will include the up-to-date clinical and safety data, will be submitted to the FDA at least annually.

9.4 Reporting to St. Jude Office of Regulatory Affairs

Copies of all correspondence to the St. Jude IRB, including SAE reports are provided to the St. Jude Regulatory Affairs Office. All FDA related correspondence and reporting will be conducted through the Regulatory Affairs Office. Adverse event reporting and annual reporting will be in accord with the FDA Title 21 CFR312.32 and Title 21 CFR312.33, respectively. The Regulatory Affairs Office can be reached at 901-595-2347 (secondary contact: St. Jude Vice President of Clinical Trials Administration 901-595-2876).

9.5 Continuing review reports

Continuing review reports of protocol progress and summaries of adverse events will be filed with the St. Jude IRB, and IBC at least annually.

9.6 Data **submission to Miltenyi Biotec**

Clinical and safety related data will be provided to Miltenyi Biotec, the manufacturer of the CliniMACS system. Data will include but is not limited to the transplant research participant's age and diagnosis, donor product(s) related information including donor type, the stem cell mobilization, selection, and infusion procedure. Outcome data including lymphohematopoietic reconstitution, immunological response, disease response and transplant complications will be shared with Miltenyi Biotec. Representatives from Miltenyi Biotec will be able to review the participant's (donor and transplant research recipient) laboratory and medical record for data verification purposes. Copies of all reports to the institutional and governing regulatory bodies will also be accessible upon request. In the event that the protocol is placed on a clinical hold by the PI or governing regulatory authorities, representatives from Miltenyi Biotec will be notified as soon as possible.

9.7 Reporting to the Center for International Blood and Marrow Transplant Research

The Transplant Program at St. Jude is required by the federal government to report transplant information to the Center for International Blood and Marrow Transplant Research (CIBMTR). The CIBMTR is a research partnership of the International Bone Marrow Transplant Registry, the National Marrow Donor Program (NMDP), and the Foundation for the Accreditation of Cellular Therapy (FACT). This organization is responsible for the collection and maintenance of a standardized data warehouse registry of autologous and all allogeneic (related and unrelated donor) transplants performed in the United States.

The Office of General Counsel, U.S. Department of Health and Human Services, had deemed the CIBMTR not a covered entity under the Privacy Rule (45 CFR 164.512), 45 CFR Parts 160 and 164, and the Health Insurance Portability and Accountability Act (HIPPA) of 1996. For this reason, the submission and disclosure of certain protected

health information (PHI), including that required for CIBMTR, is allowable without the individual's authorization (i.e. consent is waived) when such disclosure is made to public health authorities authorized by law for the purpose of preventing or controlling disease, injury, or disability.

Data resulting from this transplant procedure will be sent for general registry purposes to comply with the federal government requirements. This information for both donor and recipient is submitted using a unique participant identification number. The information submitted for haploidentical recipients is less extensive than recipients of other donor products. For this reason, variables submitted may include but are not limited to the transplant recipient's date of birth, country/state of current residence, diagnosis, basic lympho-hematopoietic reconstitution (e.g. date of ANC and platelet engraftment), post-HCT disease status, and basic AEs (e.g. GVHD- yes or no), survival status, date/cause of death.

10.0 STATISTICAL CONSIDERATIONS

10.1 Statistical Design and Analysis for the Primary Objectives and Stopping Rules

This study is designed as a phase II study. The primary objective of this study is to evaluate the rate of successful engraftment at day +42. Successful engraftment for the purposes of this objective will be patients who do not experience graft failure as defined in section 7.5.

As detailed in section 2, the use of alternative donors such as haploidentical donors and unrelated umbilical cord blood (UCB) are frequently needed and largely successful. However, the use of these alternative donors is typically complicated with additional problems, particularly poor hematopoietic recovery and graft failure.[100] Handgretinger et al found that improved engraftment was obtained in haploidentical donor transplantation with the use of a megadose (high donor CD34+ cell) T-cell depleted graft and administration of OKT3 instead of ATG.[26] This combination has since been the foundation of haploidentical donor transplantation at this institution. However, OKT3 is no longer clinically available. This protocol includes a novel preparative regimen using TLI instead of anti-T-cell antibody to facilitate engraftment. Therefore, the primary objective of this study is to assess donor engraftment, ensuring that this novel regimen facilitates donor engraftment at an acceptable rate.

The majority of published experience with alternative (HLA-mismatched) donor transplantation in children is with umbilical cord blood grafts. The COBALT study included 191 children with hematologic malignancies who received UCB transplantation with TBI based (1350Gy) myeloablative conditioning.[101] The cumulative incidence of neutrophil engraftment by day 42 was 80%. The New York Blood Center published outcomes on 1061 patients (78% pediatric) with hematologic malignancies who received myeloablative UCB transplantation with units from their bank.[102] The cumulative incidence of neutrophil engraftment was 74% by day 77. The CIBMTR published a comparison of UCB transplantation with HLA-matched unrelated donor transplantation, in which 503 children with acute leukemia received umbilical cord blood

transplantation.[103] They confirmed that although the leukemia-free survival was similar, the rate of neutrophil engraftment (and TRM) were significantly worse with HLA-mismatched UCB grafts than with HLA-matched bone marrow grafts. For UCB recipients, the best engraftment was obtained in the rare 6/6 matched UCB recipients with 85% of the 35 patients achieving neutrophil recovery at day +42. In addition, recipients who received a single antigen mismatch UCB unit with an appropriately high cell dose (n=154) had a neutrophil engraftment rate of 80% at day +42. Recipients of lower cell dose or two antigen mismatch units fared worse.

Given the data from the paragraph above, we consider a rate of successful engraftment of less than 80% by day +42 to be unacceptable for alternative donor transplantation. The goal of our study is to develop a novel conditioning regimen to facilitate successful engraftment of haploidentical donor grafts at a rate of ≥91%. We do not anticipate censoring during the 42 day time period and we can approximate the rate of successful engraftment at day +42 using a Binomial distribution. (However, patients lost for follow up will be counted as a failure to engraft in order to keep the validity of Binomial distribution approximation). Therefore, in this study, we propose to test the null hypothesis H_0: P≤0.80 versus H_1: P>0.80, where P is the proportion of research participants successfully engrafted at day +42 posttransplant. With type I error of 10% and type II error of 20%, Simon's two stage optimum design powered at alternative successful engraftment rate P_1=0.91 requires 21 evaluable patients at the first stage and 49 evaluable patients in total[76]. The stopping rules are provided in Table 1, with the understanding that stopping the trial early would be suggestive of the proposed transplant strategy not being an effective treatment option for this group of patients. The interpretation is that if we observe 17 or fewer participants successfully engrafted at day +42 in the first 21 participants, then we would stop the trial for lack of efficacy. However, if we observe 18 or more patients engrafted successfully in the first 21 participants at day +42 in stage one, then 28 more patients will be enrolled in stage two. If we observe 43 or more participants successfully engrafted at day +42 upon completion of the trial, then we conclude that the true rate of successful engraftment is at least 80% and our novel regimen will be proposed for further development and phase III clinical trial.

Table 1: Stopping rules for lack of efficacy based on the Simon's 2-stage optimum design (unacceptable low rate of successful engraftment at day +42)

Accept H_0 if the number of research participants engrafted					
P_0	P_1	(≤ r_1/n_1)	(≤ r/n)	EN(P_0)	PET(P_0)
0.80	0.91	17/21	42/49	31	0.63

Note: r_1 and r denote the number of patients successfully engrafted at day +42; EN(P_0) denotes the expected sample size under P_0; PET(P_0) denotes the probability of early termination at stage I under P_0.

All participants who receive the prescribed transplant will be evaluable for the primary outcome (engraftment at day +42). In addition, any patient who starts the conditioning regimen but stops prior to receiving the graft will count as a failure, if the reason for stopping therapy is toxicity from the conditioning regimen. Any patient who dies from

toxicity after engraftment but before day +42 will count as a successful engraftment. Any patient who dies prior to engraftment will count as a graft failure. Any patient who rejects the initial graft but is successfully engrafted with another graft will still count as a graft failure.

Participants who enroll but do not initiate the treatment (receive conditioning and infusions) due to withdrawal of participant, health status change to make the treatment not to the participant's best medical interest, withdrawal of donor participant, etc. will be replaced.

After the study is finished, for the first primary objective, the rate of engraftment at day +42 and its 95% Blyth-Still-Casella confidence interval will be estimated based on the binomial approximation.

As of 1/31/17, 42 patients have received transplantation on this study, 39 had successful engraftment, 1 patient died prior to engraftment, and 2 patients experienced graft failure but were successfully grafted with a progenitor cell boost from the same donor. This indicates that the primary objective is likely to be satisfied (42 successful engraftment) prior to completion of original protocol enrollment goal of 49 recipients. To fulfill a critical patient need, Amendment 4.0 expands the intended enrollment beyond the primary objective enrollment goal. Because this protocol provides specialized T-cell depletion and graft manipulation techniques, a therapy that is only available under the protocol IDE, future patients would not be able to potentially benefit from this therapy once the protocol completes. Preliminary analysis (data censored 10/31/16) show the 1 year EFS (events: death, relapse) in patients with non-refractory (e.g. <5% blasts) hematologic malignancy receiving first allogeneic HCT from a TCD haploidentical donor to be 86.6% (SE:8.2%) on this study compared to 55.6% (SE:8.1%) on the previous two trials (HAPREF, HIFLEX). Therefore, future patients could benefit from this therapy with the expanded enrollment goal of 75 evaluable patients. With past accrual at 12 to 15 patients per year, the study should be extended approximately 2 years. This provides the time necessary to develop and implement a replacement therapeutic protocol that builds upon the progress of this study.

Secondary benefits to the expanded enrollment on this study include increased precision of the clinical outcome estimates for the primary and secondary objectives, and the ability to compare these outcomes based on whether the donor is KIR matched or mismatched. Tertiary benefits to the expanded enrollment include increased depth of the biologic characterization of immune reconstitution, particularly of donor memory T-cells. Enhanced understanding of memory T-cell function has the potential to lead to the development of new successful therapeutics.

In addition to the stopping rules based on successful engraftment, we will closely monitor the trial for early excessive toxicities in terms of severe acute GvHD (aGvHD), and therapy related death. Therapy related death is any death in remission and related to protocol therapy. Therapy related death will be monitored for 100 days from the date of transplantation for application of the stopping rules. Most acute GVHD is expected to

occur in the first 100 days, however acute GVHD may occur after Day 100, and the GVHD syndromes in naïve T-cell depleted transplantation may differ biologically from GVHD in conventional T-cell replete settings. Therefore any acute GVHD ≥ grade III will count against the stopping rule, even if it occurs after 100 days. Toxicities secondary to non-protocol therapy for posttransplant persistent or recurrent disease will not count towards the toxicity stopping rules. Toxicities that are secondary to post-transplant therapy that is defined in the protocol (such as escalating DLI as described in section 4.6) will count towards the stopping rules. The safety endpoints will be monitored independently and a consideration to stopping the trial would be given if there is evidence suggesting that the rate of grades III-IV aGVHD is greater than 20%, or the rate of transplant related death is greater than 10%. Then, the planned interim evaluation time points and stopping rules, using group sequential design based on Pocock stopping boundaries after considering five interim analyses (p-value threshold = 0.0158) to ensure an overall type one error of 0.05. [104-106], for each of the two endpoints are provided in

Tables 2 and 3.

After this amendment 4, if the stopping rule for monitoring the engraftment is not met in the first 49 evaluable participants enrolled, then after 49 evaluable participants enrolled on the study, we will not monitor the engraftment and will estimate the engraftment rate using all evaluable participants enrolled on the study after the study is finished. However, we will continue to closely monitor excess toxicity of grade III-IV aGVHD and TRM within the first 100 days. We will conduct three more interim analyses when 60, 70 and 75 evaluable patients are enrolled on the study for monitoring these toxicities using the new monitoring rule (Tables 2-3) based on a binomial distribution and a p-value threshold of 0.05.

Table 2. Stopping Rules for Toxicities Based on Grades III-IV aGVHD

# of Research Participants Enrolled ≤ 11	# Grades III-IV aGVHD Observed [illegible]
	6
21	9
31	12
41	15
49	17
60*	18
70*	21
75*	22

*Based on a binomial distribution with a p-value threshold of 0.05 added on amendment 4.0.

Table 3. Stopping Rules for Toxicities Based on Therapy Related Death within the first 100 days Post-transplant

# of Research Participants Enrolled<br>≤<br>11	# Therapy Related Death Observed<br>□
	5
21	6
31	8
41	10
49	11
60*	11
70*	12
75*	13

*Based on a binomial distribution with a p-value threshold of 0.05 added on amendment 4.0.

Based on the above Table 2, if six grades III-IV aGVHD occur in the first eleven evaluable research participants treated, then a consideration will be given to temporarily stopping the trial and amending the study to include additional or alternative pharmacologic GVHD prophylaxis. Similarly, from Table 4 if we observe five deaths related to therapy within the first 100 days after transplantation in the first eleven evaluable research participants treated, then a consideration will be given to temporarily stopping the trial and amending the study appropriately. Likewise, if eighteen or more the grade 3-4 aGvHD within first 100 days post transplantation occur in the first 60 evaluable research participants treated, a consideration will be given to temporarily stopping the trial and amending the study to include additional or alternative pharmacologic GVHD prophylaxis. Similarly, if eleven or more TRM within first 100 days post transplantation occur in the first 60 evaluable research participants treated, then a consideration will be given to temporarily stopping the trial and amending the study appropriately.

Starting with Amendment 3.0, patients with a KIR matched haploidentical donor are eligible for this therapy, whereas before 3.0, patients could only receive transplantation from a KIR mismatched haploidentical donor. While the rate of donor engraftment, rate of acute GvHD, and rate of therapy related death are unlikely to be related to KIR matching, with this new population being added with amendment 3.0, the stopping rules for efficacy (Table 1) and toxicity (Table 2 and 3) will be applied to all recipients, to KIR matched donor recipients independently, and to KIR mismatched donor recipients independently.

Prior to amendment 5.0, as of date April 4, 2018, preliminary analysis showed a significantly higher rate of GVHD grade III-IV in patients who received maraviroc (4 of

5 = 80%) compared to those patients who did not receive maraviroc (12 of 53 = 25%) (p = 0.02). Because the use of maraviroc in these 5 patients treated on amendment 3.0 was a substantive change in therapy that affected the risk of GVHD in those recipients, those 5 patients will be excluded from analysis of the primary objective and the stopping rules above. For the secondary and exploratory objectives as detailed below, the groups (with and without maraviroc) will be analyzed separately and together.

10.2 Statistical Analysis for Secondary Objectives

10.2.1 Estimate the incidence of malignant relapse, EFS and OS at one-year posttransplantation

The estimate of cumulative incidence of relapse will be estimated using Kalbfleisch-Prentice method. Death is the competing risk event. The analysis will be implemented using SAS macro (bmacro252-Excel2007\cin) available in the St. Jude Department of Biostatistics. The Kaplan-Meier estimate of OS and EFS with relapse, death due to any cause and graft failure as events along with their standard errors will be calculated using the SAS macro (bmacro251-Excel2007\kme) available in the Department of Biostatistics at St. Jude, where OS = min (date of last follow-up, date of death) – date of HCT and all participants surviving after 1 year post-transplant will be considered as censored, and EFS = *min* (date of last follow-up, date of relapse, date of graft failure, date of death due to any cause) – date of transplant, and all participants surviving at the time of analysis without events will be censored. The analysis for this objective will be performed when the last evaluable participant has been followed for one-year post transplant.

10.2.2 Estimate the incidence and severity of acute and chronic GVHD

The cumulative incidence of acute and chronic GVHD will be estimated using Kalbfleisch-Prentice method. Death is the competing risk event. The SAS macro (bmacro252-Excel2007\cin) available in the Department of Biostatistics at St. Jude will be used for such analysis. The severity of acute GVHD and chronic GVHD will be described. The analysis for this objective will be performed when the last evaluable participant has been followed for 100 days post transplant.

10.2.3 Estimate the rate of transplant related mortality in the first 100 days after transplantation

The cumulative incidence of transplant related mortality will be estimated using the same method as used in evaluating Objective 10.2.2. Deaths before day 100 because of other reasons are the competing risk events. The analysis for this objective will be performed when the last evaluable participant has been followed for 100 days post transplant.

10.3 Analysis for Exploratory Objectives

The final results of these exploratory objectives are expected to be available when the last evaluable participant has been followed for one-year post transplant.

10.3.1 Assess the relationship between pre-transplant MRD with transplant outcomes.

The relationship of pre-transplant MRD and transplant outcomes will be examined through Cox proportional hazard model or generalized linear model. The model can also be used to adjust for other confounding factors such as patient's age at transplant.

10.3.2 Record immune reconstitution parameters, including chimerism analysis, quantitative lymphocyte subsets, T-cell receptor excision circle (TREC) analysis,

V-beta spectratyping, and phenotype and functional analysis of reconstituted lymphocyte subsets.

All immune reconstitution measures will be descriptively analyzed.

10.3.3 The use of CD45RA-depleted DLI for recipeints who have severe viral infections, disease recurrence or progression, or poor immune reconstitution will be recorded, including dose given, date given, and indication for use. To assess efficacy, the resolution of viral infection, resolution of molecular or frank disease, or the correction of severe lymphopenia will be noted. All adverse events that are at least possibly related to CD45RA-depleted DLI and any episode of GVHD will be recorded.

11.0 DATA ACQUISITION AND QUALITY ASSURANCE MONITORING

11.1 Enrollment on study

A member of the study team will confirm potential participant eligibility as defined in Section 3.1-3.2, complete and sign the 'Participant Eligibility Checklist'. The study team will enter the eligibility checklist information into the Patient Protocol Manager (PPM) system. Eligibility will be reviewed, and a research participant-specific consent form and assent document (where applicable) will be generated. The complete signed consent/assent form(s) must be faxed to the CPDMO at 595-6265 to complete the enrollment process.

The CPDMO is staffed 7:30 am-5:00 pm CST, Monday through Friday. A staff member is on call Saturday, Sunday, and holidays from 8:00 am to 5:00 pm. Enrollments may be requested during weekends or holidays by calling the CPDMO "On Call" cell phone (901-413-8591) or referencing the "On Call Schedule" on the intranet).

11.2 Data submission

The St. Jude Cancer Center Clinical Research Assistants and Associates assigned to the Department of BMTCT will assure protocol compliance, and conduct all clinical and

safety data collection. Data will be entered into an institutional database. The PI will be responsible for review of case report forms for accuracy and completeness prior to entry into the secure departmental database.

11.3 Quality assurance monitoring

This protocol will be monitored for safety and data as per the St. Jude Data and Safety Monitoring Plan for Clinical Trials approved by the NCI in 2010. The Central Protocol and Data Monitoring Office (CPDMO) will verify 100% of the informed consent documentation on all participants and verify 100% of St. Jude participants' eligibility status. The study team will meet at appropriate intervals to review case histories and data quality summaries on all participants. The St. Jude Clinical Research Monitor will assess protocol and regulatory compliance as well as the accuracy and completeness of all data points for the first two participants enrolled, then 15% of study enrollees (and their corresponding donors) every six months. The protocol will be tracked continuously for the accrual of donors and recipients. All AE and SAE reports will be reviewed by the study Principal Investigator for type, grade, attribution, duration, timeliness and appropriateness on all study participants and their corresponding donors. All SAE reports will be reviewed by the monitor every 6 months.
Protocol compliance monitoring will include participant status, eligibility, the informed consent process, demographics, staging, study objectives, subgroup assignment, treatments, evaluations, responses, participant protocol status, off-study, and off-therapy criteria. The Monitor will generate a formal report which is shared with the Principal Investigator (PI), study team and the Internal Monitoring Committee (IMC). Monitoring may be conducted more frequently if deemed necessary by the CPDMO or the IMC. Continuing reviews by the
IRB and CT-SRC will occur at least annually. In addition, SAE reports in TRACKS (Total
Research and Knowledge System) are reviewed in a timely manner by the IRB. The Regulatory Affairs Office will assist the PI in reporting to the FDA and other external oversight agencies, as necessary.

This plan is supplemented by a focused audit plan as submitted to the FDA on 9/21/15. Please see appendix G for details.

12.0 OBTAINING INFORMED CONSENT

The ongoing informed consent process will be carried out per the policies and procedures put forth in the St. Jude Investigator's Handbook for Clinical Research (http://home.web.stjude.org/ clinical research/administration/doc/handbook.pdf). The PI or physician sub-investigator will conduct the signature authorization portion of the consent process. Authorization for the recipient procedure will be conducted in the presence of an independent witness such as the St. Jude Ombudsperson/Research Participant Advocate or designee, a professional staff member from the Department of Nursing, or Social Work.

13.0 REFERENCES

1. Westoff CF: Fertility in the United States. Science 234:554-9, 1986
2. Kernan NA, Bartsch G, Ash RC, et al: Analysis of 462 transplantations from unrelated donors facilitated by the National Marrow Donor Program. N Engl J Med 328:593602, 1993
3. Barker JN, Wagner JE: Umbilical-cord blood transplantation for the treatment of cancer. Nat Rev Cancer 3:526-32, 2003
4. Beatty PG, Clift RA, Mickelson EM, et al: Marrow transplantation from related donors other than HLA-identical siblings. N Engl J Med 313:765-71, 1985
5. Aversa F, Terenzi A, Tabilio A, et al: Full haplotype-mismatched hematopoietic stem-cell transplantation: a phase II study in patients with acute leukemia at high risk of relapse. J Clin Oncol 23:3447-54, 2005
6. Ciceri F, Labopin M, Aversa F, et al: A survey of fully haploidentical hematopoietic stem cell transplantation in adults with high-risk acute leukemia: a risk factor analysis of outcomes for patients in remission at transplantation. Blood 112:3574-81, 2008
7. Ruggeri L, Capanni M, Urbani E, et al: Effectiveness of donor natural killer cell alloreactivity in mismatched hematopoietic transplants. Science 295:2097-100, 2002
8. Leung W, Iyengar R, Triplett B, et al: Comparison of killer Ig-like receptor genotyping and phenotyping for selection of allogeneic blood stem cell donors. J Immunol 174:6540-5, 2005
9. Leung W, Iyengar R, Turner V, et al: Determinants of antileukemia effects of allogeneic NK cells. J Immunol 172:644-50, 2004
10. Leung W, Campana D, Yang J, et al: High success rate of hematopoietic cell transplantation regardless of donor source in children with very high-risk leukemia. Blood 118:223-30, 2011
11. Thomas E, Storb R, Clift RA, et al: Bone-marrow transplantation (first of two parts). N Engl J Med 292:832-43, 1975
12. Thomas ED, Buckner CD, Banaji M, et al: One hundred patients with acute leukemia treated by chemotherapy, total body irradiation, and allogeneic marrow transplantation.
    Blood 49:511-33, 1977
13. Hahn T, Wall D, Camitta B, et al: The role of cytotoxic therapy with hematopoietic stem cell transplantation in the therapy of acute lymphoblastic leukemia in children: an evidence-based review. Biol Blood Marrow Transplant 11:823-61, 2005
14. Oliansky DM, Rizzo JD, Aplan PD, et al: The role of cytotoxic therapy with hematopoietic stem cell transplantation in the therapy of acute myeloid leukemia in children: an evidence-based review. Biol Blood Marrow Transplant 13:1-25, 2007
15. Appelbaum FR, Fisher LD, Thomas ED: Chemotherapy v marrow transplantation for adults with acute nonlymphocytic leukemia: a five-year follow-up. Blood 72:179-84, 1988
16. Butturini A, Gale RP: Chemotherapy versus transplantation: II. Acute lymphoblastic leukemia. Haematologica 74:337-9, 1989

17. Barrett AJ, Horowitz MM, Gale RP, et al: Marrow transplantation for acute lymphoblastic leukemia: factors affecting relapse and survival. Blood 74:862-71, 1989
18. Sarina B, Castagna L, Farina L, et al: Allogeneic transplantation improves the overall and progression-free survival of Hodgkin lymphoma patients relapsing after autologous transplantation: a retrospective study based on the time of HLA typing and donor availability. Blood 115:3671-7, 2010
19. Gross TG, Hale GA, He W, et al: Hematopoietic stem cell transplantation for refractory or recurrent non-Hodgkin lymphoma in children and adolescents. Biol Blood Marrow Transplant 16:223-30, 2010
20. Schultz KR, Bowman WP, Aledo A, et al: Improved early event-free survival with imatinib in Philadelphia chromosome-positive acute lymphoblastic leukemia: a children's oncology group study. J Clin Oncol 27:5175-81, 2009
21. Biondi A, Schrappe M, De Lorenzo P, et al: Imatinib after induction for treatment of children and adolescents with Philadelphia-chromosome-positive acute lymphoblastic leukaemia (EsPhALL): a randomised, open-label, intergroup study. Lancet Oncol 13:936-45
22. Oliansky DM, Camitta B, Gaynon P, et al: Role of cytotoxic therapy with hematopoietic stem cell transplantation in the treatment of pediatric acute lymphoblastic leukemia: update of the 2005 evidence-based review. Biol Blood Marrow Transplant 18:505-22 23. Henslee-Downey PJ, Abhyankar SH, Parrish RS, et al: Use of partially mismatched related donors extends access to allogeneic marrow transplant. Blood 89:3864-72, 1997
24. Rocha V, Kabbara N, Ionescu I, et al: Pediatric related and unrelated cord blood transplantation for malignant diseases. Bone Marrow Transplant 44:653-9, 2009
25. Aversa F: Haploidentical haematopoietic stem cell transplantation for acute leukaemia in adults: experience in Europe and the United States. Bone Marrow Transplant 41:473-81, 2008
26. Handgretinger R, Klingebiel T, Lang P, et al: Megadose transplantation of purified peripheral blood CD34(+) progenitor cells from HLA-mismatched parental donors in children. Bone Marrow Transplant 27:777-83, 2001
27. Lang P, Handgretinger R: Haploidentical SCT in children: an update and future perspectives. Bone Marrow Transplant 42 Suppl 2:S54-9, 2008
28. Goldman FD, Rumelhart SL, DeAlacron P, et al: Poor outcome in children with refractory/relapsed leukemia undergoing bone marrow transplantation with mismatched family member donors. Bone Marrow Transplant 25:943-8, 2000
29. Aversa F, Terenzi A, Felicini R, et al: Mismatched T cell-depleted hematopoietic stem cell transplantation for children with high-risk acute leukemia. Bone Marrow Transplant 22 Suppl 5:S29-32, 1998
30. Aversa F, Tabilio A, Velardi A, et al: Treatment of high-risk acute leukemia with T-cell-depleted stem cells from related donors with one fully mismatched HLA haplotype. N Engl J Med 339:1186-93, 1998
31. Handgretinger R, Schumm M, Lang P, et al: Transplantation of megadoses of purified haploidentical stem cells. Ann N Y Acad Sci 872:351-61; discussion 361-2, 1999

32. Chen X, Hale GA, Barfield R, et al: Rapid immune reconstitution after a reducedintensity conditioning regimen and a CD3-depleted haploidentical stem cell graft for paediatric refractory haematological malignancies. Br J Haematol 135:524-32, 2006
33. Woodard P, Cunningham JM, Benaim E, et al: Effective donor lymphohematopoietic reconstitution after haploidentical CD34+-selected hematopoietic stem cell transplantation in children with refractory severe aplastic anemia. Bone Marrow Transplant 33:411-8, 2004
34. Handgretinger R, Klingebiel T, Lang P, et al: Megadose transplantation of highly purified haploidentical stem cells: current results and future prospects. Pediatr Transplant 7 Suppl 3:51-5, 2003
35. Handgretinger R, Chen X, Pfeiffer M, et al: Feasibility and outcome of reducedintensity conditioning in haploidentical transplantation. Ann N Y Acad Sci 1106:279-89, 2007 36. Hale G, Chen X, Benaim E, et al: Haploidentical transplantation for children with refractory haematologic malignancies. Bone Marrow Transplant 37:S250, 2006
37. Karre K, Ljunggren HG, Piontek G, et al: Selective rejection of H-2-deficient lymphoma variants suggests alternative immune defence strategy. Nature 319:675-8, 1986
38. Long EO: Regulation of immune responses through inhibitory receptors. Annu Rev Immunol 17:875-904, 1999
39. Moretta A, Pende D, Locatelli F, et al: Activating and inhibitory killer immunoglobulin-like receptors (KIR) in haploidentical haemopoietic stem cell transplantation to cure high-risk leukaemias. Clin Exp Immunol 157:325-31, 2009
40. Ruggeri L, Mancusi A, Burchielli E, et al: NK cell alloreactivity and allogeneic hematopoietic stem cell transplantation. Blood Cells Mol Dis 40:84-90, 2008
41. Vilches C, Parham P: KIR: diverse, rapidly evolving receptors of innate and adaptive immunity. Annu Rev Immunol 20:217-51, 2002
42. Ruggeri L, Capanni M, Casucci M, et al: Role of natural killer cell alloreactivity in HLA-mismatched hematopoietic stem cell transplantation. Blood 94:333-9, 1999
43. Moretta A, Bottino C, Mingari MC, et al: What is a natural killer cell? Nat Immunol 3:6-8, 2002
44. Ji SQ, Chen HR, Wang HX, et al: G-CSF-primed haploidentical marrow transplantation without ex vivo T cell depletion: an excellent alternative for high-risk leukemia. Bone Marrow Transplant 30:861-6, 2002
45. Godder KT, Hazlett LJ, Abhyankar SH, et al: Partially mismatched related-donor bone marrow transplantation for pediatric patients with acute leukemia: younger donors and absence of peripheral blasts improve outcome. J Clin Oncol 18:1856-66, 2000
46. Champlin RE, Passweg JR, Zhang MJ, et al: T-cell depletion of bone marrow transplants for leukemia from donors other than HLA-identical siblings: advantage of T-cell antibodies with narrow specificities. Blood 95:3996-4003, 2000
47. Amrolia PJ, Muccioli-Casadei G, Huls H, et al: Adoptive immunotherapy with allodepleted donor T-cells improves immune reconstitution after haploidentical stem cell transplantation. Blood 108:1797-808, 2006

48. Ciurea SO, Mulanovich V, Jiang Y, et al: Lymphocyte recovery predicts outcomes in cord blood and T cell-depleted haploidentical stem cell transplantation. Biol Blood Marrow Transplant 17:1169-75, 2011
49. Novitzky N, Davison GM: Immune reconstitution following hematopoietic stemcell transplantation. Cytotherapy 3:211-20, 2001
50. Maltezou HC, Kafetzis DA, Abisaid D, et al: Viral infections in children undergoing hematopoietic stem cell transplant. Pediatr Infect Dis J 19:307-12, 2000
51. Andre-Schmutz I, Dal Cortivo L, Fischer A, et al: Improving immune reconstitution while preventing GvHD in allogeneic stem cell transplantation. Cytotherapy 7:102-8, 2005
52. Walter EA, Greenberg PD, Gilbert MJ, et al: Reconstitution of cellular immunity against cytomegalovirus in recipients of allogeneic bone marrow by transfer of T-cell clones from the donor. N Engl J Med 333:1038-44, 1995
53. Leen AM, Christin A, Myers GD, et al: Cytotoxic T lymphocyte therapy with donor T cells prevents and treats adenovirus and Epstein-Barr virus infections after haploidentical and matched unrelated stem cell transplantation. Blood 114:4283-92, 2009
54. Perruccio K, Tosti A, Burchielli E, et al: Transferring functional immune responses to pathogens after haploidentical hematopoietic transplantation. Blood 106:4397-406, 2005
55. Anderson BE, McNiff J, Yan J, et al: Memory CD4+ T cells do not induce graftversus-host disease. J Clin Invest 112:101-8, 2003
56. Dahlke MH, Larsen SR, Rasko JE, et al: The biology of CD45 and its use as a therapeutic target. Leuk Lymphoma 45:229-36, 2004
57. Dutton RW, Bradley LM, Swain SL: T cell memory. Annu Rev Immunol 16:20123, 1998
58. Chao N: Memory T cells. Biol Blood Marrow Transplant 14:17-9, 2008
59. Faint JM, Annels NE, Curnow SJ, et al: Memory T cells constitute a subset of the human CD8+CD45RA+ pool with distinct phenotypic and migratory characteristics. J Immunol 167:212-20, 2001
60. Ochsenbein AF, Pinschewer DD, Sierro S, et al: Protective long-term antibody memory by antigen-driven and T help-dependent differentiation of long-lived memory B cells to short-lived plasma cells independent of secondary lymphoid organs. Proc Natl Acad Sci U S A 97:13263-8, 2000
61. Jameson SC, Masopust D: Diversity in T cell memory: an embarrassment of riches. Immunity 31:859-71, 2009
62. Yamashita N, Bullington R, Clement LT: Equivalent helper functions of human "naive" and "memory" CD4+ T cells for the generation of alloreactive cytotoxic T lymphocytes. J Clin Immunol 10:237-46, 1990
63. Distler E, Bloetz A, Albrecht J, et al: Alloreactive and leukemia-reactive T cells preferentially derive from naive precursors in healthy donors: implications for immunotherapy with memory T cells. Haematologica, 2011
64. Chen BJ, Cui X, Sempowski GD, et al: Transfer of allogeneic CD62L- memory T cells without graft-versus-host disease. Blood 103:1534-41, 2004

65. Lu SY, Liu KY, Liu DH, et al: High frequencies of CD62L naive regulatory T cells in allografts are associated with a low risk of acute graft-versus-host disease following unmanipulated allogeneic haematopoietic stem cell transplantation. Clin Exp Immunol 165:26477, 2011
66. Crucitti L, Crocchiolo R, Toffalori C, et al: Incidence, risk factors and clinical outcome of leukemia relapses with loss of the mismatched HLA after partially incompatible hematopoietic stem cell transplantation. Leukemia 29:1143-52, 2015
67. Chen YB, Spitzer TR: Current status of reduced-intensity allogeneic stem cell transplantation using alternative donors. Leukemia 22:31-41, 2008
68. Aversa F, Terenzi A, Felicini R, et al: Haploidentical stem cell transplantation for acute leukemia. Int J Hematol 76 Suppl 1:165-8, 2002
69. Aversa F, Velardi A, Tabilio A, et al: Haploidentical stem cell transplantation in leukemia. Blood Rev 15:111-9, 2001
70. Benaim E, Hale G, Horwitz E, et al: Reduced-intensity conditioning (RIC) in haploidentical transplantation. Blood 104:590a, 2004
71. Ortin M, Raj R, Kinning E, et al: Partially matched related donor peripheral blood progenitor cell transplantation in paediatric patients adding fludarabine and anti-lymphocyte gamma-globulin. Bone Marrow Transplant 30:359-66, 2002
72. O'Donnell PV, Luznik L, Jones RJ, et al: Nonmyeloablative bone marrow transplantation from partially HLA-mismatched related donors using posttransplantation cyclophosphamide. Biol Blood Marrow Transplant 8:377-86, 2002
73. Rizzieri DA, Koh LP, Long GD, et al: Partially matched, nonmyeloablative allogeneic transplantation: clinical outcomes and immune reconstitution. J Clin Oncol 25:690-7, 2007
74. Ogawa H, Ikegame K, Yoshihara S, et al: Unmanipulated HLA 2-3 antigen-mismatched (haploidentical) stem cell transplantation using nonmyeloablative conditioning. Biol Blood Marrow Transplant 12:1073-84, 2006
75. Kohrt H, Lowsky R: Total lymphoid irradiation for graft-versus-host disease protection. Curr Opin Oncol 21 Suppl 1:S23-6, 2009
76. Kohrt HE, Turnbull BB, Heydari K, et al: TLI and ATG conditioning with low risk of graft-versus-host disease retains antitumor reactions after allogeneic hematopoietic cell transplantation from related and unrelated donors. Blood 114:1099-109, 2009
77. Lowsky R, Takahashi T, Liu YP, et al: Protective conditioning for acute graftversus-host disease. N Engl J Med 353:1321-31, 2005
78. Heinzelmann F, Lang PJ, Ottinger H, et al: Immunosuppressive total lymphoid irradiation-based reconditioning regimens enable engraftment after graft rejection or graft failure in patients treated with allogeneic hematopoietic stem cell transplantation. Int J Radiat Oncol Biol Phys 70:523-8, 2008
79. Palma J, Salas L, Carrion F, et al: Haploidentical stem cell transplantation for children with high-risk leukemia. Pediatr Blood Cancer

80. Lewalle P, Triffet A, Delforge A, et al: Donor lymphocyte infusions in adult haploidentical transplant: a dose finding study. Bone Marrow Transplant 31:39-44, 2003
81. Morton AJ, Gooley T, Hansen JA, et al: Association between pretransplant interferon-alpha and outcome after unrelated donor marrow transplantation for chronic myelogenous leukemia in chronic phase. Blood 92:394-401, 1998
82. Rizzieri DA, Storms R, Chen DF, et al: Natural killer cell-enriched donor lymphocyte infusions from A 3-6/6 HLA matched family member following nonmyeloablative allogeneic stem cell transplantation. Biol Blood Marrow Transplant 16:1107-14
83. Miller JS, Soignier Y, Panoskaltsis-Mortari A, et al: Successful adoptive transfer and in vivo expansion of human haploidentical NK cells in patients with cancer. Blood 105:3051-7, 2005
84. Karre K: Immunology. A perfect mismatch. Science 295:2029-31, 2002
85. Triplett BM, Horwitz EM, Iyengar R, et al: Effects of activating NK cell receptor expression and NK cell reconstitution on the outcomes of unrelated donor hematopoietic cell transplantation for hematologic malignancies. Leukemia 23:1278-87, 2009
86. Savani BN, Mielke S, Adams S, et al: Rapid natural killer cell recovery determines outcome after T-cell-depleted HLA-identical stem cell transplantation in patients with myeloid leukemias but not with acute lymphoblastic leukemia. Leukemia 21:2145-52, 2007
87. Stroncek DF, Clay ME, Smith J, et al: Changes in blood counts after the administration of granulocyte-colony-stimulating factor and the collection of peripheral blood stem cells from healthy donors. Transfusion 36:596-600, 1996
88. Trzonkowski P, Zaucha JM, Mysliwska J, et al: Differences in kinetics of donor lymphoid cells in response to G-CSF administration may affect the incidence and severity of acute GvHD in respective HLA-identical sibling recipients. Med Oncol 21:81-94, 2004
89. Coustan-Smith E, Behm FG, Sanchez J, et al: Immunological detection of minimal residual disease in children with acute lymphoblastic leukaemia. Lancet 351:550-4, 1998
90. Coustan-Smith E, Sancho J, Behm FG, et al: Prognostic importance of measuring early clearance of leukemic cells by flow cytometry in childhood acute lymphoblastic leukemia.
Blood 100:52-8, 2002
91. Coustan-Smith E, Sancho J, Hancock ML, et al: Clinical importance of minimal residual disease in childhood acute lymphoblastic leukemia. Blood 96:2691-6, 2000
92. Nucifora G, Larson RA, Rowley JD: Persistence of the 8;21 translocation in patients with acute myeloid leukemia type M2 in long-term remission. Blood 82:712-5, 1993
93. Roberts WM, Estrov Z, Ouspenskaia MV, et al: Measurement of residual leukemia during remission in childhood acute lymphoblastic leukemia. N Engl J Med 336:31723, 1997

94. Knechtli CJ, Goulden NJ, Hancock JP, et al: Minimal residual disease status as a predictor of relapse after allogeneic bone marrow transplantation for children with acute lymphoblastic leukaemia. Br J Haematol 102:860-71, 1998
95. Langebrake C, Creutzig U, Dworzak M, et al: Residual disease monitoring in childhood acute myeloid leukemia by multiparameter flow cytometry: the MRD-AML-BFM Study Group. J Clin Oncol 24:3686-92, 2006
96. Bleakley M, Heimfeld S, Loeb KR, et al: Outcomes of acute leukemia patients transplanted with naive T cell-depleted stem cell grafts. J Clin Invest, 2015
97. Nassif A, Bensussan A, Boumsell L, et al: Toxic epidermal necrolysis: effector cells are drug-specific cytotoxic T cells. J Allergy Clin Immunol 114:1209-15, 2004
98. Scott-Lang V, Tidman M, McKay D: Toxic epidermal necrolysis in a child successfully treated with infliximab. Pediatr Dermatol 31:532-4, 2014
99. Filipovich AH, Weisdorf D, Pavletic S, et al: National Institutes of Health consensus development project on criteria for clinical trials in chronic graft-versus-host disease: I. Diagnosis and staging working group report. Biol Blood Marrow Transplant 11:945-56, 2005
100. Ballen KK, Koreth J, Chen YB, et al: Selection of optimal alternative graft source: mismatched unrelated donor, umbilical cord blood, or haploidentical transplant. Blood 119:1972-80
101. Kurtzberg J, Prasad VK, Carter SL, et al: Results of the Cord Blood Transplantation Study (COBLT): clinical outcomes of unrelated donor umbilical cord blood transplantation in pediatric patients with hematologic malignancies. Blood 112:4318-27, 2008
102. Barker JN, Scaradavou A, Stevens CE: Combined effect of total nucleated cell dose and HLA match on transplantation outcome in 1061 cord blood recipients with hematologic malignancies. Blood 115:1843-9, 2010
103. Eapen M, Rubinstein P, Zhang MJ, et al: Outcomes of transplantation of unrelated donor umbilical cord blood and bone marrow in children with acute leukaemia: a comparison study. Lancet 369:1947-54, 2007
104. Blyth CR, Still HA: Binomial Confidence-Intervals. Journal of the American Statistical Association 78:108-116, 1983
105. Blyth CR: Approximate Binomial Confidence-Limits. Journal of the American Statistical Association 81:843-855, 1986
106. Casella G, R.L. B: Statistical Inference. Belmont, CA, Wadsworth&Brooks/Cole, 1990

APPENDIX A:

KARNOFSKY PERFORMANCE STATUS SCALE<br>≥ 16 YEARS OLD	
**Score**	**General Description**
100	Normal. No complaints. No evidence of disease.
90	Able to carry on normal activity. Minor signs or symptoms of disease.
80	Normal activity with effort. Some signs or symptoms of disease.
70	Care of self. Unable to carry out normal activity or to do active work.
60	Requires occasional assistance, but is able to care for most of his needs.
50	Requires considerable assistance and frequent medical care.
40	Disabled. Requires special care and assistance.
30	Severely disabled. Hospitalization is indicated although death is not imminent.
20	Hospitalization necessary, very sick, active support treatment necessary.
10	Moribund. Fatal processes progressing rapidly.
0	Dead.

LANSKY PERFORMANCE STATUS SCALE<br>< 16 YEARS OLD	
**Score**	**General Description**
100	Fully active, normal
90	Minor restrictions in physically strenuous activity
80	Active, but tires more quickly
70	Both greater restriction of and less time spent in play activity
60	Up and around, but minimal active play; keeps busy with quieter activities
50	Gets dressed but lies around much of the day, no active play but able to participate in all quiet play and activities
40	Mostly in bed; participates in quiet activities
30	In bed; needs assistance even for quiet play
20	Often sleeping; play entirely limited to very passive activities
10	No play; does not get out of bed
0	Unresponsive

APPENDIX B

COG STEM CELL COMMITTEE CONSENSUS GUIDELINES FOR ESTABLISHING ORGAN STAGE AND OVERALL GRADE OF ACUTE GRAFT VERSUS HOST DISEASE (GVHD)

Table 1 outlines standard criteria for GVHD organ staging. However, confounding clinical syndromes (such as non-GVHD causes of hyperbilirubinemia) may make staging GVHD in a given organ difficult. In addition, timing of organ specific symptoms affects whether that symptom is more or less likely to be true GVHD. Please refer to ***Tables 2 and 3*** to assist you in deciding whether to attribute these clinical findings to GVHD, especially in situations where a biopsy is not possible. For additional help, please see the text which follows the tables. ***Table 4*** reviews the approach to assessing GVHD as acute, chronic, or the overlap between the two.

Finally, ***engraftment syndrome*** will be reported separately from the GVHD scoring presented below.

Engraftment Syndrome

A clinical syndrome of fever, rash, respiratory distress, and diarrhea has been described, just prior to engraftment in patients undergoing unrelated cord blood and mismatched transplantation. If, in the judgment of the treating physician, a patient experiences this syndrome, details of the event will be recorded in the medical record.

Modified Glucksberg Staging Criteria for Acute Graft versus Host Disease

Table 1: Organ Staging (See tables and text below for details)

Stage	Skin	Liver (bilirubin)	Gut (stool output/day)
**0**	No GVHD rash	< 2 mg/dL	Adult: < 500 mL/day Child: < 10 mL/kg/day
**1**	Maculopapular rash < 25% BSA	2-3 mg/dL	Adult: 500-999 mL/day Child: 10-19.9 mL/kg/day. ***Or persistent nausea, vomiting, or anorexia, with a positive upper GI biopsy.***
**2**	Maculopapular rash 25-50% BSA	3.1-6 mg/dL	Adult: 1000-1500 mL/day Child: 20-30 mL/kg/day
**3**	Maculopapular rash > 50% BSA	6.1-15 mg/dL	Adult: > 1500 mL/day Child: > 30 mL/kg/day
**4**	Generalized erythroderma plus bullous formation and desquamation > 5% BSA	>15 mg/dL	Severe abdominal pain with or without ileus, or grossly bloody stool (regardless of stool volume).

For GI staging: The “adult” stool output values should be used for patients > 50 kg in weight. Use 3 day averages for GI staging based on stool output. If stool and urine are mixed, stool output is presumed to be 50% of total stool/urine mix (see 3.2 below).

For Stage 4 GI: the term “severe abdominal pain” will be defined as:

a) Pain control requiring institution of opioid use, or an increase in on-going opioid use, PLUS
b) Pain that significantly impacts performance status, as determined by the treating MD.

If colon or rectal biopsy is +, but stool output is < 500 mL/day (< 10 mL/kg/day), then consider as GI stage 0.

There is no modification of liver staging for other causes of hyperbilirubinemia.

Overall Clinical Grade (based on the highest stage obtained):

Grade 0: No stage 1-4 of any organ
Grade I: Stage 1-2 skin and no liver or gut involvement
Grade II: Stage 3 skin, or Stage I liver involvement, or Stage l GI
Grade III: Stage 0-3 skin, with Stage 2-3 liver, or Stage 2-3 GI
Grade IV: Stage 4 skin, liver or GI involvement

Table 2 Evaluating Liver GVHD in the Absence of Biopsy Confirmation (See Table 3.0 below)

Establishing liver GVHD with no skin or GI GVHD

**No Skin/GI GVHD Day 0-35**	Assume no liver GVHD, unless proven by biopsy	
**No Skin/GI GVHD Day 36-100**	If NO other etiology identified, NO improvement with stopping hepatotoxic medications/TPN: **Stage as liver GVHD**	If other etiology identified or improves with stopping hepatotoxic drugs/TPN: **Do not stage as liver GVHD**

Establishing liver GVHD with skin or GI GVHD and other cause of hyperbilirubinemia

**Skin and/or GI GVHD present**	Worsening bilirubin level (includes worsening just prior to onset of skin or GI tract GVHD) OR stable elevated bilirubin despite resolution of non-GVHD cause of increased bilirubin: **Stage as liver GVHD**	Stable or improving bilirubin after diagnosis of skin or GI GVHD, irrespective of treatment: **Do not stage as liver GVHD**

Changing liver GVHD stage with other cause of hyperbilirubinemia

**Skin and GI GVHD stable, improving, or absent**	Liver GVHD staging is carried forward without increase in stage until other disease process resolves (e.g., if TTP is diagnosed in the presence of stage 2 liver GVHD, the liver GVHD stage 2 is carried forward despite rising bilirubin level until TTP is resolved. If there is no liver GVHD – stage 0 – and new onset TTP, the stage 0 is carried forward until TTP is resolved).
**Skin and/or GI GVHD worsening**	**Liver GVHD is staged according to the Glucksberg criteria. The elevated bill is attributed to GVHD alone**.<br><br>Thus, when skin or GI GVHD is worsening, there is no downgrading of liver GVHD staging for other causes of hyperbilirubinemia. (e.g., if TTP is diagnosed in the presence of stage 2 liver GVHD and worsening skin or GI GVHD, the liver is staged according to the actual bilirubin level even if some of the rise in bilirubin is attributed to TIP).<br><br>Similarly, even if there is no liver GVHD at onset of a new process, (such as TPN cholestasis), but skin or GI GVHD worsen during that process, then liver GVHD is diagnosed and staged according to the height of the bilirubin.<br><br>**There is one exception to this**: the diagnosis of TTP, with high LDH and **unconjugated** bilirubin precludes the diagnosis and staging of new liver GVHD in the absence of a confirmatory liver biopsy.

Table 3 Evaluating GI GVHD in the Absence of Biopsy Confirmation
(See Table 4.0 below)

Establishing GI GVHD with new onset diarrhea and no skin or liver GVHD

**No skin/liver GVHD**<br>**Day 0 through engraftment**	Assume no GI GVHD, unless proven by biopsy	
**No skin/liver GVHD engraftment through Day 100**	NO other etiology of diarrhea identified:<br>**Stage as GI GVHD**	Any other etiology of diarrhea identified:<br>**Do not stage as GI GVHD**

Establishing GI GVHD with pre-existing diarrhea and skin or liver GVHD

**Skin and/or liver GVHD present**	Worsening diarrhea (includes worsening just prior to onset of skin or liver GVHD) OR persistent diarrhea despite resolution of non-GVHD cause:<br>**Stage as GI GVHD**	Improving diarrhea after the diagnosis of skin or liver GVHD (irrespective of treatment) OR persistent diarrhea without resolution of underlying non-GVHD cause:<br>**Do not stage as GI GVHD**

Differentiating Acute GVHD, Chronic GVHD, and Overlap Syndrome:
There is often confusion differentiating acute from chronic GVHD, especially in the setting of reduced intensity transplants, DLI and new prophylactic treatments. The NIH Working Group recently published new classifications for GVHD:

Table 4 Acute GVHD, Chronic GVHD, and Overlap Syndrome

**Category**	**Time of Symptoms after HCT or DLI**	**Presence of Acute GVHD features**	**Presence of Chronic GVHD features**
**Acute GVHD**			
Classic acute GVHD	<100 d	Yes	No
Persistent, recurrent, or late-onset acute	>100 d	Yes	No
GVHD**Chronic GVHD**			
Classic chronic GVHD	No time limit	No	Yes
Overlap syndrome	No time limit	Yes	Yes

- Scoring of acute GVHD may need to occur past day 100. In particular, patients should continue to be scored for acute GVHD when classic acute GVHD symptoms (maculopapular rash, nausea, vomiting, anorexia, profuse diarrhea - particularly if bloody and ileus) persist past day 100 or if identical symptoms previously scored as acute GVHD resolve and then recur within 30 days during immunosuppression taper but past day 100.

- Those patients being scored as having acute GVHD should NOT have diagnostic or distinctive signs of chronic GVHD.

- **Patients with both acute and chronic symptoms should be diagnosed as having Overlap Syndrome and scored according to their chronic GVHD score**.

Further Explanation of Criteria presented in Tables 2 and 3

1.0 Assessment of Skin GVHD

1.1 Presence or Absence of Skin GVHD: Skin GVHD will be considered present if a rash characteristic of acute GVHD develops after allogeneic marrow transplantation involving more than 25% of the body surface not clearly attributable to causes such as drug administration or infection. The extent of the body surface area involved can be estimated by the "Rule of Nines". In estimating the extent of skin GVHD, the area involved is calculated for individual anatomic areas, such as the arm or leg, and then the total is derived from a simple summation. Areas that are non-blanching should not be considered involved regardless of the overlying color of the rash (red, brown, etc.).

Limited distribution erythema (with the exception of palms and soles) in the absence of associated rash elsewhere on the body will not be considered GVHD.

2.0 Assessment of Liver GVHD

2.1 Assessing for the Presence or Absence of Liver GVHD

A. Hyperbilirubinemia (total bilirubin $\geq$ 2.0 mg/dL) in the **absence** of other signs of acute GVHD in the skin or GI tract:

i) Day 0-35: If hyperbilirubinemia alone is present with no other signs of acute GVHD in other organ systems, acute GVHD will not be diagnosed based solely on laboratory abnormalities.

Acute GVHD will be diagnosed if findings on histopathology studies of liver from a biopsy or autopsy are confirmatory.

ii) Day 35-100: If hyperbilirubinemia (must be conjugated bilirubin) is not improving or is exacerbated (especially if serum alkaline phosphatase is increased), in the absence of acute GVHD in other organ systems, no other etiologies are identified, and does not improve with discontinuation of hepatotoxic drugs, acute GVHD will be diagnosed. However, it is distinctly unusual to develop ascites or a coagulopathy in the early stages of acute GVHD of the liver alone. In the absence of histopathology studies of liver from a biopsy or autopsy specimen, ascites or a coagulopathy secondary to liver dysfunction will be considered to indicate the presence of another disease process (e.g., veno-occlusive disease). Recommended non-invasive studies to define an etiology for hyperbilirubinemia are:

a. Imaging of liver (CT or ultrasound)
b. Hepatitis screen (only if ALT is elevated)
c. PT
d. Blood cultures
e. Review of medication list for potentially hepatotoxic drugs
f. Review of risk factors for viral liver infection (HSV, CMV, VZV, adenovirus, EBV, HBV, and HCV)
g. Hemolysis screen

B. Pre-existing hyperbilirubinemia clearly attributed to an etiology other than acute GVHD in the presence of signs of acute GVHD in other organ systems.

i) If pre-existing non-GVHD liver disease (documented clinically, by lab assessment, or by imaging studies) is stable or improving at the onset of signs of

acute GVHD in other organs, then acute GVHD of the liver will not be considered to be present unless proven by liver biopsy or autopsy.

ii) If hyperbilirubinemia worsens several days before or at the time of onset of signs of acute GVHD in other organ systems, GVHD will be considered to be present unless histopathology studies of liver are available and negative on a biopsy during that time interval or autopsy results exclude GVHD.

iii) If hyperbilirubinemia persists and is not improving after resolution of a pre-existing non-GVHD liver disease process (e.g., localized infection of liver, systemic sepsis, biliary tract obstruction) when signs of acute GVHD are present in other organ systems or no other intervening cause has been diagnosed, then acute GVHD will be considered to be present in the absence of a new, clearly identifiable cause of nonGVHD liver disease or unless a liver biopsy or autopsy specimen is negative.

Prior acute GVHD in liver with new onset of a disease process that exacerbates preexisting or recently resolved hyperbilirubinemia:

i) If an etiology other than acute GVHD is clearly identified as causing or exacerbating hyperbilirubinemia and acute liver GVHD has been diagnosed and has been stable, improving, or resolved, then the liver will not be restaged for acute GVHD until the resolution or stabilizing of the concurrent disease process (i.e., the liver stage prior to the onset of the new disease process will be carried forward until the new disease process resolves). Example: Acute GVHD of the liver and gut is diagnosed on day 20. Treatment of acute GVHD results in falling bilirubin levels to liver stage 1. Sepsis or TTP develops with transient worsening of the hyperbilirubinemia. The liver stage is not increased, despite a higher bilirubin level, because the cause of worsening hyperbilirubinemia is attributed to sepsis or TTP.

ii) If an etiology other than acute GVHD is clearly identified as causing or exacerbating hyperbilirubinemia in the presence of already worsening acute liver GVHD **<u>or</u>** GVHD of the skin or GI tract is simultaneously worsening, then the liver GVHD will be staged according to the actual bilirubin level, even though another cause of hyperbilirubinemia is present.

3.0 <u>Assessment of GVHD of the Gastrointestinal Tract</u>

3.1 Assessing for the Presence or Absence of GVHD of the Gastrointestinal Tract

A. Diarrhea ($\geq$500 mL/day in adults or > 10 mL/kg in pediatric patients) in the absence of other signs of acute GVHD in other organ systems

i) Day 0-engraftment: If diarrhea alone is present without other signs of acute GVHD in other organ systems, acute GVHD will not be considered present. Diarrhea will be attributed to acute GVHD if histopathology studies of gastrointestinal tract from a biopsy or autopsy are diagnostic.

ii) Engraftment-day 100: If diarrhea persists and is not improving, is exacerbated, or develops de novo in the absence of acute GVHD in other organ systems, histopathology studies of gut biopsies or from autopsy specimens are not available, and no other etiologies are clearly identified, acute GVHD will be considered to be the cause. A stool specimen should be examined to rule out infectious causes (e.g., rotavirus, adenovirus, and C. difficile toxin). It is recommended, if at all possible, that biopsies be obtained for diagnostic purposes.

B. Pre-existing diarrhea clearly attributed to an etiology other than acute GVHD in the presence of signs of acute GVHD in other organ systems:

i) If pre-existing diarrhea caused by a process other than GVHD has been documented clinically or by lab assessment and is stable or improving at the onset of signs of acute GVHD in the skin or liver, then acute GVHD of the intestine will not be considered to be present in the absence of biopsy confirmation or autopsy report.

ii) If diarrhea or gastrointestinal symptoms are already present, but worsen significantly at the time of onset of signs of acute GVHD in the skin or liver, GVHD will be considered present, unless biopsy or autopsy are negative.

iii) If diarrhea persists after resolution of a pre-existing disease process with signs of acute GVHD present in other organ systems, GVHD will be considered present, unless biopsy or autopsy are negative.

C. Prior or present acute GVHD in other organ systems with new onset of diarrhea:

If diarrhea is clearly attributable to an etiology other than acute GVHD (e.g., infection) and a history of acute GVHD exists or acute GVHD is present in other organ systems and is stable, then the gastrointestinal tract will not be evaluable for acute GVHD until the resolution or stabilizing of the other disease process (e.g., infection) in the absence of biopsy or autopsy confirmation.

D. Persistent anorexia, nausea or vomiting in the absence of signs of acute GVHD in other organ systems:

Persistent anorexia, nausea or vomiting in the absence of other known causes of these symptoms will be considered stage I acute GVHD if confirmed by endoscopic biopsy.

If a biopsy is not possible (e.g. secondary to thrombocytopenia) but the clinical findings are compatible with acute GVHD, then the patient will be treated and recorded as having acute GVHD.

3.2 Staging of the Gastrointestinal Tract for the Severity of Acute GVHD

The severity of gastrointestinal tract GVHD will be staged according to modified Glucksberg criteria. To minimize errors caused by large day-to-day variation, diarrhea volume is measured as an average over 3 days and reported as the volume in milliliters per day. When urinary mixing is noted the stool volume will be considered half of the total volume unless nursing staff is able to give a better estimate from direct observation. Abdominal cramps are considered significant for staging if the severity results in a clinical intervention (e.g. analgesia, fasting, etc.). Blood in the stools is considered significant if the blood is visible or hematochezia/melena is present and not clearly attributed to a cause other than GVHD (e.g., epistaxis/hemorrhoids).

APPENDIX C
CRITERIA FOR GRADING CHRONIC GVHD GRADE

	**Score 0**	**Score 1**	**Score 2**	**Score 3**
**Performance Score:**<br>________<br>**KPS ECOG LPS**	☐Asymptomatic and fully active (ECOG 0: KPS or LPS 100%)	☐ Symptomatic, fully ambulatory, restricted only in physically strenuous activity (ECOG 1, KPS or LPS 80-90%)	☐Symtomatic, ambulatory, capable of self-care, >50% of waking hours out of bed (ECOG 2, KPS or LPS 60-70%)	☐Symptomatic limited self-care, >50% of waking hours in bed (ECOG 3-4, KPS or LPS <60%)
**SKIN**<br>*Clinical features:*<br>☐Maculopapular rash<br>☐Lichen planus-like features<br>☐Papuloquamous lesions or ichthyosis<br>☐Hyperpigmentation<br>☐Hypopigmentaion<br>☐Keratosis pilaris<br>☐Erythema<br>☐Erythroderma<br>☐Poikiloderma<br>☐Sclerotic features<br>☐Pruritus<br>☐Hair involvement<br>☐Nail involvement<br>**% BSA Involved ______**	☐ No symptoms	☐<18% BSA with disease signs but **NO**sclerotic features	☐ 19-50% BSA **OR** involvement with superficial sclerotic features "not hidebound" (able to pinch)	☐>50% BSA **OR**deep sclerotic features "hidebound" (unable to pinch) **OR** impaired mobility, ulceration or severe pruritus
**MOUTH**	☐No symptoms	☐ Mild symptoms with disease signs but **NOT**limiting oral intake significantly	☐ Moderate symptoms with disease signs **WITH**partial limitation of oral intake	☐ Severe symptoms with disease signs on examination **WITH** major limitation of oral intake
**EYES**<br><br>☐**Mean tear test (mm):**<br>☐**>10**<br>☐**6-10**<br>☐**>5**<br>☐**Not done**	☐No symptoms	☐ Mild dry eye symptoms not affecting ADL (requiring eyedrops) ≤3 x per day) **OR** asymptomatic signs of keratoconjunctivitissiccca	☐ Moderate dry eye symptoms partially affecting ADL (requiring drops≥3x per day or punctual plugs), **WITHOUT**vision impairment	☐ Severe dry eye symptoms significantly affecting ADL (special eyeware to relieve pain) **OR**unable to work because of ocular symptoms**OR** loss of vision cause by keratoconjunctivitissicca
**GI Tract**	☐No symptoms	☐ Symptoms such as dysphagia, anorexia, nausea, vomiting, abdominal pain or diarrhea without significant weight loss (<5%)	☐ Symptoms associated with mild to moderate weight loss) 5-15%)	☐ Symptoms associated with significant weight loss > 15%, requires nutritional supplement for most calorie needs **OR**esophageal dilation
**LIVER**	☐ Normal LFT	☐ Elevated Bilirubin, AP*, AST or ALT <2 x ULN	☐ Bilirubin >3 mg/dl or Bilirubin, enzymes 2-3 x ULN	☐ Bilirubin or enzymes > 5 x ULN

APPENDIX C (continued)

CRITERIA FOR GRADING CHRONIC GVHD GRADE

	SCORE 0	SCORE 1	SCORE 2	SCORE 3
**Lungs†**<br><br>FEV1 ___________<br><br>DLCO __________	☐ No symptoms<br><br>☐ FEV1 >80% **OR** LFS=2	☐ Mild symptoms (shortness of breath after climbing one flight of steps)<br><br>☐ FEV1 60-79% **OR** LFS 3-5	☐ Moderate symptoms (shortness of breath after walking on flat ground)<br><br>☐ FEV1 40-59% **OR** LFS 6-9	Severe symptoms (shortness of breath at rest; requiring 0_2<br><br>☐ FEV1 ≥39% **OR** LFS 10-12
**JOINTS AND FASCIA**	☐ No symptoms	☐ Mild tightness of arms or legs, normal or mild decreased range of motion (ROM) **AND** not affecting ADL	☐ Tightness of arms or legs **OR** joint contractures, erythema thought due to fasciitis, moderate decrease ROM **AND** mild to moderate limitation of ADL	☐Contractures WITH significant decrease or ROM AND significant limitation of ADL (unable to tie shoes, button shirts, dress self etc.)
**GENITAL TRACT**	☐ No symptoms	☐ Symptomatic with mild signs on exam **AND** no effect on coitus and minimal discomfort with gynecologic exam	☐ Symptomatic with moderate signs on exam **AND** with mild dyspareunia or discomfort with gynecologic exam	☐ Symptomatic **WITH** advance signs (stricture, labial agglutination or severe ulcerations **AND** severe pain with coitus or inability it insert vaginal speculum

Other indicators, clinical manifestations or complications related to chronic GVHD (check all that apply and assign a score to its severity (0-3) based on its functional impact where applicable (none = 0, mild = 1, moderate =2, severe = 3.

Esophageal stricture or web ____ Pericarial Effusion ____ Pleural Effusion(s) ____

Ascites (serositis) ____ Nephrotic syndrome ____ Peripheral Neuropathy ____

Myasthenia Gravis ____ Cardiomyopathy ____ Eosinophilia > 500/µl ____

Polymyositis ____ Cardiac conduction defects ____ Coronary artery involvement ____

Platelets <100,000/µl ____ Progressive Onset ____

Other: Specify: ____

Organ scoring of chronic GVHD.

* AP may be elevated in growing children, and not reflective of liver dysfunction.

† Pulmonary scoring should be performed using both the symptom and pulmonary function testing (PFT) scale whenever possible. When discrepancy exists between pulmonary symptoms or PFT scores, the higher value should be used for final scoring. Scoring using the Lung Function Score (LFS**)** **i**s preferred, but if DLCO is not available, grading using FEV1 should be used. The LFS is a global assessment of lung function after the diagnosis of bronchiolitis obliterans has already been established. The percent predicted FEV1 and DLCO (adjusted for hematocrit but not alveolar volume) should be converted to a numeric score as follows: >80% = 1; 70-79% = 2; 60-69% = 3; 50-59% = 4; 40-49% = 5; <40% = 6. The LFS = FEV1 score + DLCO score, with a possible range of 2-12. GVHD indicates graft versus host disease, ECOG, Eastern Cooperative Oncology Group, KPS, Karnofsky Performance Status; LPS, Lansky Performance Status; BSA, body surface area; ADL, activities of daily living; LFTs, liver function tests; AP, alkaline phosphatase; ALT, alanine aminotransferase; AST, aspartate aminotransferase; ULN, upper limit of normal.

APPENDIX C (continued)

CRITERIA FOR GRADING CHRONIC GVHD GRADE

GLOBAL GRADING OF CHRONIC GVHD[84]

Final Grade	Number of Organs/Sites*	Maximum Organ Score**	Lung Score
Mild	1 - 2	1	0
Moderate	3 or more	1	1
	At least 1	2	
Severe	At least 1	3	2 - 3

*Determined by adding the total number of organs receiving score > 0 using Figure 1, Appendix B.

**Defined as the maximum score given to any organ system amongst all organs scored using Figure 1, Appendix B.

APPENDIX D

CRITERIA FOR ADVERSE EVENT (AE) EVALUATION AND REPORTING

The St. Jude Department of BMTCT Clinical Research Office standard operating procedure for the documenting and reporting of adverse (SOP 10 Documenting and Reporting of Adverse Events) will provide guidance on the evaluation, collection and reporting of adverse events for this clinical trial. The current version of this document, as well as ongoing updates, can be located at the following website: http://home.stjude.org/bmt/Pages/policies-research.aspx

APPENDIX E

Recommended testing and evaluation schedule

STANDARD OF CARE STUDIES	SAMPLE	VOLUME	PRE	MONTH 1	MONTH 2	MONTH 3	MONTH 6	MONTH 12
**Pregnancy Test**	PB	2 ml	X	As clinically indicated				
**Physical Exam**	N/A	N/A	X	Weekly			X	X
**CBC with diff.**	PB	0.5-2 ml	X	Daily until engrafted, then weekly			X	X
**Chemistry**	PB	0.25-2ml	X	Weekly			X	X
**Viral surveillance (BMTPCR)**	PB	4 ml	X	Weekly			As clinically indicated	
**Chimerism**	PB	1-2 ml		Weekly upon engraftment			X	X
	BM	2 ml		X		X		X
**Disease Status Evaluation**	N/A	N/A	X	X		X		X
**MRD Bone Marrow**	BM	3 ml	X	X		X		X
**Lymphocyte Subset Study**	PB	2.5-4 ml	X	X	X	X	X	X
**Quantitative Immunoglobulins**	PB	2 ml	X			X		X

- The information derived from or noted on the physical examinations, standard tests, and other assessments that comprise standard of care for recipients are not required to be transcribed onto case report forms and/or entered into the database. In reference to section 6.1 Evaluations, the above-indicated follow-up regimen for these evaluations is guided by the SOPs of the Department of BMTCT, for recipients of allogeneic stem cell transplantation. As these evaluations are considered standard clinical care

(nonresearch), variations in frequency (more or less frequent) of these evaluations can occur due to the participant's current clinical condition and will not be noted as protocol deviations.

- Disease status evaluations/BM testing results obtained prior to enrollment may be used for the baseline/pre-infusion assessments.
- Additional chimerism testing may be required as clinically indicated and described in Section 6.3.
- Lymphocyte subset studies may be omitted without variance when the absolute lymphocyte count (ALC) is zero.
- In the event of graft failure/rejection, the post failure/rejection bone marrow, chimerism and several applicable immune studies would not be clinically indicated and these studies may be held.

APPENDIX E (continued)

Immune reconstitution testing and evaluation schedule for RECIPIENT

RESEARCH STUDIES	SAMPLE	VOLUME	PRE	MONTH 1	MONTH 2	MONTH 3	MONTH 6	MONTH 12
**VBETA/TREC RESEARCH**	PB	17 mL	X			X	X	X
**LYMPHOCYTE PHENOTYPES RESEARCH**	PB	17 mL	X	X	X	X	X	X
**IR-PHENOTYPE (Youngblood Lab)**	PB	5 mL	X	X	X	X	X	X
**T-FUNCTION (Thomas Lab)**	PB	5 mL	X	X	X	X	X	X
	BM	1 mL	X	X		X		

- VBETA/TREC Research and Lymphocyte Phenotypes Research results will be maintained in the Immune Monitoring Core Lab.
- For RESEARCH studies, the posted volumes are the minimum volumes required to perform the respective protocol evaluations.
- The PRE-transplant bone marrow for T-FUNCTION are optional tests that may be omitted at the discretion of the PI or co-investigator.

APPENDIX E (continued) Research testing for DONOR

Prior to initial stem cell collection procedure:

OPTIONAL research immune studies testing schedule

Evaluation	*Volume Requirement*
Flow cytometry enumeration	Lymphocyte Subset Study = 4 mL
Thymic output and T cell repertoire	VBETA/TREC Research = 17 mL
Donor baseline lymphocyte number and function	Lymphocyte Phenotypes Research = 17 mL
Donor baseline immune function (Youngblood Lab)	IR-PHENOTYPE = 5mL
Donor baseline immune function (Thomas Lab)	T-FUNCTION = 5mL

- All donor research testing to be collected prior to growth factor administration. These optional research tests may be collected at separate times.
- Research testing results will be maintained in a secured database in the respective co-investigator laboratory database.

APPENDIX E (continued)

Research Study Evaluation Target Windows

Several laboratory tests can only be processed on weekdays; therefore, if the scheduled evaluation falls on a weekend, or during a holiday period, an adjustment in the follow-up visit is expected and would not be noted as a protocol variation. Additionally, in order to accommodate such logistical constraints, evaluation/collection dates of all protocol assessments (required and optional research), may be performed within a reasonable window of the intended date following the guidelines provided in the table below:

**If the Planned Evaluation Time Point is:**	**Window**
Weekly	± 3 Days
Month 1	Week 2 to Week 6
Month 2	Week 7 to Week 11
Month 3	Week 12 to Month 4
Month 6	Month 5 to Month 7
Month 9	Month 8 to Month 10
Month 12	Month 10 to Month 14

APPENDIX F

The St. Jude Department of BMTCT Clinical SOPs for standard of care for all allogeneic stem cell infusion recipients and stem cell donors will provide guidance on the evaluation, ongoing clinical care and follow up for this clinical trial. The current versions of these SOPs, as well as ongoing updates, of these documents can be located at the following website: http://home.web.stjude.org /bone_marrow/clinicalHome.shtml.

APPENDIX G

HAPNK1 Audit Plan with Amendment 2.0

I. All new enrollments will monitored and audited by a designated experienced monitor from our CPDMO (i.e., Protocol Office). Following our institutional process, each new participant data will be reviewed as follows:

1. On the same day of enrollment the CPMDO staff will review the completed eligibility checklist and informed consent document for completeness and accuracy (100% of enrollments).

2. In support of compliance with Eligibility and Informed Consent document and process there will be verification of supporting source documents early post enrollment, within 5 days (100% of enrollments).

3. For the initial six subjects enrolled on Amendment 2.0 (or later protocol versions until 6 new participants are enrolled post activation of Amendment 2.0) the CPMDO monitor will evaluate the following components in each patient within 10 working days of the participant reaching the day +100 milestone:

   a. compliance with treatment including protocol directed GVHD prophylaxis
   b. adverse events and grade 3-4 toxicities including those contributing to the protocol stopping rules
   c. assessment and coding of acute GVHD
   d. evaluation of and reporting of deaths on study (including treatment related mortality, TRM)
   e. status of participant at day +100
   f. documentation of engraftment and,
   g. review of source data elements related to assessments for GHVD (for example, pathology biopsy results).

4. In each case the monitor will compare patient data in the protocol database with source documents. The monitor will utilize the defined criteria as indicated in the HAPNK1 latest protocol version for consent, eligibility, and toxicity assessments and grading, including the newly incorporated COG GVHD Guidelines.

II. A patient specific audit summary will be generated with findings from each of the components. Results of the patient-specific audit will be provided to the study Principal Investigator (PI) for comment and independently to the VP Clinical Trials Administration. In case of discrepancies related to under reporting of GVHD and TRM, those cases will be interpreted as assigned by the monitor in the context of operationalizing the stopping rules, and will remain as such (i.e. coded as GVHD) until the opinion of an external medical reviewer has been obtained.

III. A final consensus audit summary on the first six enrollments on Amendment 2.0 will be generated and provided to the PI, to the St. Jude Clinical Director. the VP Clinical Trials, St. Jude IRB, as well as to the FDA

IV. On a monthly basis the study PI, the study CRA, and the Director of Clinical Trial Operations in the BMT service will meet to review ongoing data collection for each enrollment for accuracy, completeness, and timeliness.

V. If the focused audits of the first six subjects enrolled on Amendment 2.0 reveal no major compliance concerns, the study will continue with an every six month review cycle as required by our Institutional Data Safety Monitoring Plan (with a modification that every other new enrollment will have data audits). In addition to the review elements noted above other components that occur after day +100 (chronic GVHD, other AEs, deaths, status, regulatory documents, etc.) will be reviewed. An overall study monitoring report will generated every six months, and will be reviewed independently at such time by the Institutional Monitoring Committee.
